# Functional Connectivity in Antipsychotic-Treated and Antipsychotic-Naive Patients With First-Episode Psychosis and Low Risk of Self-harm or Aggression: A Secondary Analysis of a Randomized Clinical Trial

**DOI:** 10.1001/jamapsychiatry.2021.1422

**Published:** 2021-06-23

**Authors:** Sidhant Chopra, Shona M. Francey, Brian O’Donoghue, Kristina Sabaroedin, Aurina Arnatkeviciute, Vanessa Cropley, Barnaby Nelson, Jessica Graham, Lara Baldwin, Steven Tahtalian, Hok Pan Yuen, Kelly Allott, Mario Alvarez-Jimenez, Susy Harrigan, Christos Pantelis, Stephen J. Wood, Patrick McGorry, Alex Fornito

**Affiliations:** 1 Turner Institute for Brain and Mental Health, Monash University School of Psychological Sciences, Clayton, Victoria, Australia; 2 Monash Biomedical Imaging, Monash University, Clayton, Victoria, Australia; 3 Orygen, Parkville, Victoria, Australia; 4 Centre for Youth Mental Health, The University of Melbourne, Melbourne, Victoria, Australia; 5 Melbourne Neuropsychiatry Centre, Department of Psychiatry, University of Melbourne & Melbourne Health, Melbourne, Victoria, Australia; 6 Melbourne School of Population and Global Health, The University of Melbourne, Melbourne, Victoria, Australia; 7 Department of Social Work, Monash University, Caulfield, Victoria, Australia; 8 University of Birmingham School of Psychology, Edgbaston, United Kingdom

## Abstract

**Importance:**

Altered functional connectivity (FC) is a common finding in resting-state functional magnetic resonance imaging (rs-fMRI) studies of people with psychosis, yet how FC disturbances evolve in the early stages of illness, and how antipsychotic treatment influences these disturbances, remains unknown.

**Objective:**

To investigate longitudinal FC changes in antipsychotic-naive and antipsychotic-treated patients with first-episode psychosis (FEP).

**Design, Setting, and Participants:**

This secondary analysis of a triple-blind, randomized clinical trial was conducted over a 5-year recruitment period between April 2008 and December 2016 with 59 antipsychotic-naive patients with FEP receiving either a second-generation antipsychotic or a placebo pill over a treatment period of 6 months. Participants were required to have low suicidality and aggression, to have a duration of untreated psychosis of less than 6 months, and to be living in stable accommodations with social support. Both FEP groups received intensive psychosocial therapy. A healthy control group was also recruited. Participants completed rs-fMRI scans at baseline, 3 months, and 12 months. Data were analyzed from May 2019 to August 2020.

**Interventions:**

Resting-state functional MRI was used to probe brain FC. Patients received either a second-generation antipsychotic or a matched placebo tablet. Both patient groups received a manualized psychosocial intervention.

**Main Outcomes and Measures:**

The primary outcomes of this analysis were to investigate (1) FC differences between patients and controls at baseline; (2) FC changes in medicated and unmedicated patients between baseline and 3 months; and (3) associations between longitudinal FC changes and clinical outcomes. An additional aim was to investigate long-term FC changes at 12 months after baseline. These outcomes were not preregistered.

**Results:**

Data were analyzed for 59 patients (antipsychotic medication plus psychosocial treatment: 28 [47.5%]; mean [SD] age, 19.5 [3.0] years; 15 men [53.6%]; placebo plus psychosocial treatment: 31 [52.5%]; mean [SD] age, 18.8 [2.7]; 16 men [51.6%]) and 27 control individuals (mean [SD] age, 21.9 [1.9] years). At baseline, patients showed widespread functional dysconnectivity compared with controls, with reductions predominantly affecting interactions between the default mode network, limbic systems, and the rest of the brain. From baseline to 3 months, patients receiving placebo showed increased FC principally within the same systems; some of these changes correlated with improved clinical outcomes (canonical correlation analysis *R* = 0.901; familywise error–corrected *P* = .005). Antipsychotic exposure was associated with increased FC primarily between the thalamus and the rest of the brain.

**Conclusions and Relevance:**

In this secondary analysis of a clinical trial, antipsychotic-naive patients with FEP showed widespread functional dysconnectivity at baseline, followed by an early normalization of default mode network and cortical limbic dysfunction in patients receiving placebo and psychosocial intervention. Antipsychotic exposure was associated with FC changes concentrated on thalamocortical networks.

**Trial Registration:**

ACTRN12607000608460

## Key Points

**Question:**

Are there different patterns of brain connectivity in antipsychotic-treated and antipsychotic-naive patients with psychosis, and how do these patterns evolve over time?

**Findings:**

In this secondary analysis of a triple-blind, longitudinal, placebo-controlled, randomized clinical trial in antipsychotic-naive patients, patients showed widespread brain dysconnectivity at baseline relative to healthy controls. Patients treated with therapy and placebo or with therapy and antipsychotics showed evidence of circuit-specific and treatment-specific normalization of connectivity over time.

**Meaning:**

In this study, some prominent baseline connectivity differences in first-episode psychosis normalized with both psychosocial therapy and placebo early in the illness course, with antipsychotics exerting circuit-specific effects.

## Teaser

This secondary analysis of a randomized clinical trial investigates differential patterns of brain connectivity in antipsychotic-treated and antipsychotic-naive patients with psychosis and how these patterns change over time.

## Introduction

Psychosis is thought to emerge from disrupted communication between large-scale functional brain networks.^1,2^ Resting-state functional magnetic resonance imaging (rs-fMRI) has been widely used to map altered functional connectivity (FC) in patients across different illness stages and within specific corticosubcortical^3,4^ and corticocortical networks.^5,6^ Numerous causes of altered FC in patients with psychosis have been proposed, including abnormal neurodevelopment,^7^ dopamine dysfunction,^8,9^ traumatic early-life experiences,^10^ and the neuromodulatory effect of antipsychotic medication.^11^ In particular, widespread and early treatment of patients with first-episode psychosis (FEP) with antipsychotics has made it difficult to disentangle the effects of medication from other factors, such as illness progression or nonpharmacological treatments, on FC.

Cross-sectional studies have shown widespread hypoconnectivity in antipsychotic-naive patients compared with healthy controls, with decreased connectivity between subcortical, frontal, and temporal regions.^12,13,14^ Hyperconnectivity has also been reported between the thalamus and primary sensory cortices^15^ and within the default mode network.^15,16,17^ These results suggest that not all FC abnormalities can be explained by medication. Nonetheless, longitudinal studies provide evidence of FC normalization after antipsychotic exposure,^11^ particularly within fronto-striatal-thalamic circuits^12,18,19^ and corticolimbic^18,20,21^ and corticocortical systems,^4,22^ which is sometimes correlated with symptom improvement.^4,18^

To our knowledge, no prior study has compared longitudinal FC changes in patients who were medicated and nonmedicated, making it impossible to disentangle FC changes attributable to antipsychotic medication vs other factors, such as the natural course of the illness or adjunct interventions. This comparison can be done only through a randomized placebo-controlled study of antipsychotic-naive patients. We recently used such a design to show, using a noninferiority design, that the placebo group had comparable clinical and functional outcomes to the medicated group^23^ and that patients who were medicated and unmedicated show different trajectories of gray matter volume, with antipsychotics normalizing basal ganglia volume in early illness stages.^23^

Here, we report a secondary analysis of FC in this cohort. Patients with FEP were scanned at baseline while antipsychotic-naive and then again at 3 and 12 months after randomization to receive treatment with either antipsychotic medication plus intensive psychosocial treatment (MIPT) or placebo plus intensive psychosocial treatment (PIPT). Our primary aims were to (1) identify FC differences between patients and controls at baseline; (2) compare FC changes over time in antipsychotic-treated patients and antipsychotic-naive patients during the initial stages of psychotic illness; and (3) examine whether any observed FC changes were associated with changes in symptoms or functional outcomes. We additionally investigated longer-term FC changes at the 12-month follow-up, after a period of time in which some participants in the placebo group had been exposed to antipsychotics.

## Methods

### Study Design

This secondary analysis of a randomized clinical trial was conducted over a 5-year recruitment period between April 2008 and December 2016 at the Early Psychosis Prevention and Intervention Centre, which is part of Orygen Youth Health in Melbourne, Australia. Patients were randomized to 1 of 2 groups: 1 group was given MIPT, and the other was given PIPT. A third healthy control group who received no intervention was also recruited. For both patient groups, the controlled treatment period spanned 6 months. Clinical assessments and MRIs were conducted at baseline, 3 months, and a final follow-up of 12 months. The preregistered primary and secondary clinical trial outcome measures were the Social and Occupational Functioning Assessment Scale (SOFAS) and the Brief Psychiatric Rating Scale (BPRS) total scores, respectively. The trial was registered with the Australian New Zealand Clinical Trials Registry in November 2007 and received ethics approval from the Melbourne Health Human Research and Ethics committee. We report here on a secondary analysis of FC measures, which was not part of the preregistered protocol. All participants gave written informed consent after having the study fully explained to them; parental consent was also obtained for participants younger than 18 years. This study followed the Consolidated Standards of Reporting Trials (CONSORT) reporting guideline. Further research and safety information can be found in the trial protocol in Supplement 1, the MRI protocol in Supplement 2, and eMethods 1 to 3, eTables 1 and 2, and eFigure 1 in Supplement 3.^24^

### Participants

**Figure 1.**
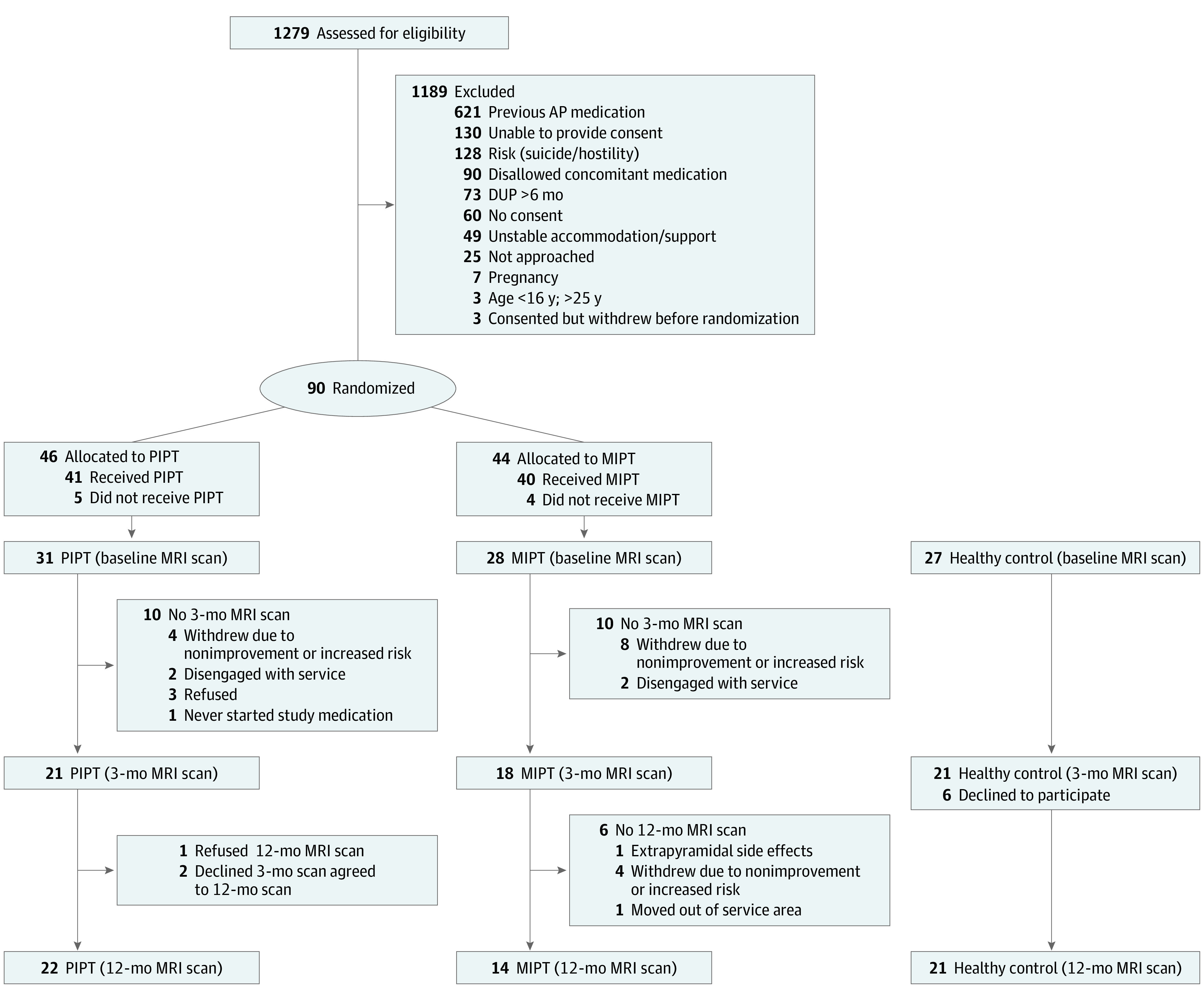
Consolidated Standards of Reporting Trials (CONSORT) Participant Flow Diagram AP indicates antipsychotic; DUP, duration of untreated psychosis; MIPT, AP medication plus intensive psychotherapy; MRI, magnetic resonance imaging; PIPT, placebo plus intensive psychotherapy.

Patients were aged 15 to 25 years and were experiencing a first episode of psychosis, defined as fulfilling the structured clinical interview for *DSM-IV* criteria for a psychotic spectrum disorder. Additional inclusion criteria to minimize risk included (1) duration of untreated psychosis of less than 6 months, (2) living in stable accommodations, (3) low risk to self or others, and (4) no or minimal previous exposure to antipsychotic medication. Healthy control participants were aged between 18 and 25 years and were psychiatrically, neurologically, and medically healthy (Figure 1 contains a detailed participant flow diagram).

### MRI Acquisition and Processing

Whole-brain T2*-weighted echo-planar images and anatomical T1-weighted scans were acquired for each participant using a 3-T Siemens Trio Tim scanner (Siemens Healthineers). Of a total of 202 rs-fMRI data sets, 193 scans survived our quality control and standard image processing procedures. To generate whole-brain FC matrices, we parceled each individual’s normalized scans into 300 cortical^25^ and 32 subcortical regions.^26^ Further details can be found in eMethods 4 and 5 in Supplement 3.

### Statistical Analysis

Nonparametric mixed-effects marginal models^27^ were used to analyze brain-wide FC changes across the 3 groups (MIPT, PIPT, and controls) and 3 time points (baseline, 3 months, and 12 months). The Network Based Statistic^28^ (NBS) was used to perform familywise error-corrected (FWE) inference at the level of connected components of edges, with the primary component-forming threshold, τ, set to *P* < .05. Further statistical details and results for τ = 0.01 and τ = 0.001 are reported in eMethods 6 and 7, eAppendixes 1 to 3, and eFigure 4 in Supplement 3.

We evaluated NBS results using a Bonferroni-corrected threshold of FWE *P* < .016, adjusted for 3 key contrasts^29^: (1) a contrast assessing baseline differences between healthy controls and patients; (2) a contrast isolating differential FC changes over time in the PIPT group (excluding 3-month scans for 4 patients in the PIPT group who were exposed to antipsychotics), compared with the healthy control group; and (3) a contrast isolating the specific effects of antipsychotic treatment by examining differential FC changes over time in the MIPT group compared with both the PIPT and healthy control groups. We repeated the same procedure for the secondary analysis of longer-term antipsychotic-naive and antipsychotic-related effects after the trial treatment period, this time including the 12-month time point (eMethods 6 and 7 in Supplement 3). We used nonparametric canonical correlation analysis^30^ to investigate associations between FC changes (ΔFC) within any identified NBS subnetworks and changes in the 2 preregistered^24^ trial outcome measures (SOFAS and BPRS) across all patients (details in eMethods 6 and 7 in Supplement 3). All statistical analyses were conducted in R Studio, version 1.3.1073 (R Studio Team). All *P* values were 2-sided, and data were analyzed from May 2019 to August 2020.

## Results

### Antipsychotic-Naive Effects

#### Baseline

A total of 59 patients (antipsychotic medication plus psychosocial treatment: 28 [47.5%]; mean [SD] age, 19.5 [3.0] years; 15 men [53.6%]; placebo plus psychosocial treatment: 31 [52.5%]; mean [SD] age, 18.8 [2.7] years; 16 men [51.6%]) and 27 controls (mean [SD] age, 21.9 [1.9] years; 17 women [63.0%]) completed a baseline scan; 39 patients (66.1%) and 21 controls (77.8%) completed a 3-month follow-up scan; and 35 patients (59.3%) and 21 controls (77.8%) completed a 12-month follow-up scan. Further information on demographic characteristics of the MIPT, PIPT, and control groups are presented in the Table and described in eMethods 6 and 7 in Supplement 3.

**Table.** Participant Characteristics

Characteristic	Mean (SD)		
	First-episode psychosis		Healthy controls (n = 27)
	PIPT (n = 31)	MIPT (n = 28)	
Baseline age, y	18.8 (2.7)	19.5 (3.0)	21.9 (1.9)
Women, No. (%)	15 (48.4)	13 (46.2)	17 (63)
Men, No. (%)	16 (51.6)	15 (53.6)	10 (37.0)
Left-handedness, No. (%)	2 (6.5)	2 (7.1)	3 (11.1)
Education, y	11.7 (1.8)	12.6 (2.3)	15.2 (1.9)
Baseline BPRS total, points^a^	59.2 (9.5)	55.6 (10.3)	NA
Baseline SOFAS, points^b^	53.0 (14.0)	52.4 (10.1)	NA
Baseline SANS, points^c^	35.7 (17.5)	32.8 (18.4)	NA
Baseline HAM-D, points^d^	19.0 (7.1)	18.4 (5.8)	NA
Baseline HAM-A, points^e^	21.6 (7.5)	19.6 (6.6)	NA
Baseline QLS, points^f^	66.7 (26.7)	71.6 (20.4)	NA
Diagnosis, No.			
Major depression with psychosis	8	5	NA
Schizophreniform disorder	4	4	
Psychotic disorder NOS	8	7	
Substance-induced psychotic disorder	5	2	
Delusional disorder	1	4	
Schizophrenia	5	5	
Missing diagnosis	0	1	
Substance use (WHO ASSIST score)			
Total	38.5 (33.7)	37.2 (29.9)	NA
Cannabis	8.7 (11.2)	8.2 (9.8)	
Tobacco	10.8 (11.2)	12.5 (10.7)	
Hallucinogens	2.1 (5.3)	1.7 (2.6)	
Amphetamines	3.2 (5.0)	2.6 (4.2)	

Abbreviations: BPRS, Brief Psychiatric Rating Scale; HAM-A, Hamilton Anxiety Scale-Anxiety; HAM-D, Hamilton Depression Scale-Depression; MIPT, antipsychotic medication plus intensive psychosocial treatment; NA, not applicable; NOS, not otherwise specified; PIPT, placebo plus intensive psychosocial treatment; QLS, Quality of Life Scale; SANS, Scale for the Assessment of Negative Symptoms; SOFAS, Social and Occupational Functioning Assessment Scale; WHO ASSIST, World Health Organization Alcohol, Smoking and Substance Involvement Screening Test.

^a^ BPRS total scores range from 24 to 168, with higher scores indicating worse symptoms.

^b^ SOFAS scores range from 1 to 100, with higher scores indicating better functioning.

^c^ SANS scores range from 0 to 125, with higher scores indicating worse symptoms.

^d^ HAM-D scores range from 0 to 50, with higher scores indicating worse symptoms.

^e^ HAM-A scores range from 0 to 56, with higher scores indicating worse symptoms.

^f^ QLS scores range from 0 to 132, with higher scores indicating better quality of life.

**Figure 2.**
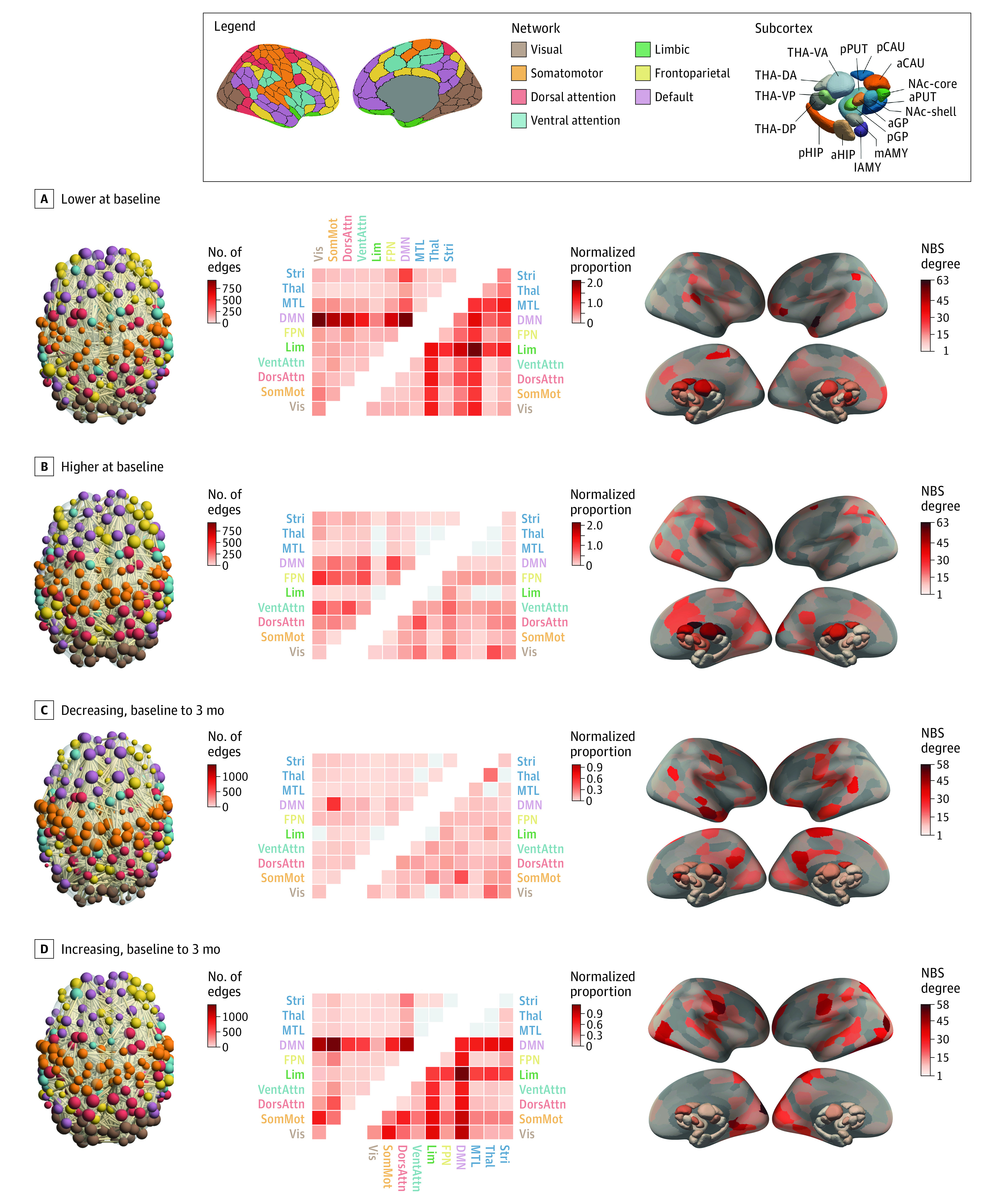
Baseline and Short-term Longitudinal Functional Connectivity Changes in Antipsychotic (AP)–Naive Patients Legend shows the Schaefer et al^28^ network and Tian et al^29^ subcortex parcellations (Scale II). Each panel contains (left to right) (1) visualization of the significant Network Based Statistic (NBS) subnetwork, with nodes colored by network and weighted by degree; (2) heatmap of the proportion of edges within the NBS component that fall within each of the canonical networks, quantified using raw (upper triangle) and normalized (lower triangle) proportions; and (3) surface renderings depicting the number of edges in the NBS subnetwork attached to each brain region (NBS degree). a Indicates anterior; AMY, amygdala; CAU, caudate nucleus; d, dorsal; DA, dorsoanterior; DMN, default mode network; DorsAttn, dorsal attention network; DP, dorsoposterior; FPN, frontoparietal network; GP, globus pallidus; HIP, hippocampus; l, lateral; Lim, cortical limbic network; m, medial; MTL, medial-temporal lobe (amygdala and hippocampus); NAc, nucleus accumbens; p, posterior; SomMot, somatomotor network; Stri, striatum; PUT, putamen; THA, thalamus; Thal, thalamus; v, ventral; VA, ventroanterior; VentAttn, ventral attention network; Vis, visual network; and VP, ventroposterior.

We identified a single NBS component showing widespread FC differences in patients compared with controls, comprising 4087 edges linking all 316 regions (FWE *P* = .02). Within this network, 2195 edges (53.7%) showed lower FC, and 1892 edges (46.3%) showed higher FC in patients (Figure 2A and B).

Using raw proportions, connections associated with reduced FC in patients were predominantly concentrated in the default mode network (DMN) (Figure 2A). Using normalized proportions, which emphasize network involvement after accounting for differences in network size (eMethods 6 and 7 in Supplement 3), limbic and medial-temporal lobe areas were also strongly implicated. At a regional level, the left superior temporal pole, inferior frontal and parietal cortices, and the right postcentral gyrus, ventroanterior thalamus, and posterior caudate were among the areas most strongly implicated in the network of FC reductions (Figure 2A). Edges in which FC was higher in patients showed a more homogeneous distribution across different networks (Figure 2B). Strongly implicated regions included the right posterior caudate, dorsoanterior and ventroanterior thalamus, left frontal eye field, left cuneus, right anterior caudate, and superior lateral occipital cortex.

#### Baseline to 3 Months

We identified a single widespread NBS component showing an altered FC trajectory in the PIPT group compared with controls, comprising 4128 edges linking all 316 regions (FWE *P* = .007). Within this network, 1913 edges (46.3%) showed decreasing FC and 2215 edges (53.7%) showed increasing FC in patients over time (Figure 2C and D). The edges showing declining FC over time in patients in the PIPT group had a diffuse distribution across different systems, with some evidence for preferential involvement of FC between the default mode and somatomotor systems (Figure 2C). The right posterior and anterior middle temporal gyrus, dorsoanterior thalamus, bilateral precuneus and posterior cingulate cortex, and left precentral gyrus were among the areas linked to many of the FC reductions over time (Figure 2C).

Edges showing increased FC in patients in the PIPT group over time were strongly concentrated in the DMN, using both raw and normalized counts. Normalized counts additionally implicated the cortical limbic system. Both of these networks showed reduced FC in patients compared with controls at baseline. Regions showing many FC increases over time included the right polar and lateral occipital cortices; bilateral postcentral, precentral, and lingual gyri; and the left lateral occipital and parietal cortices (Figure 2D).

#### Associations With Clinical Outcomes

**Figure 3.**
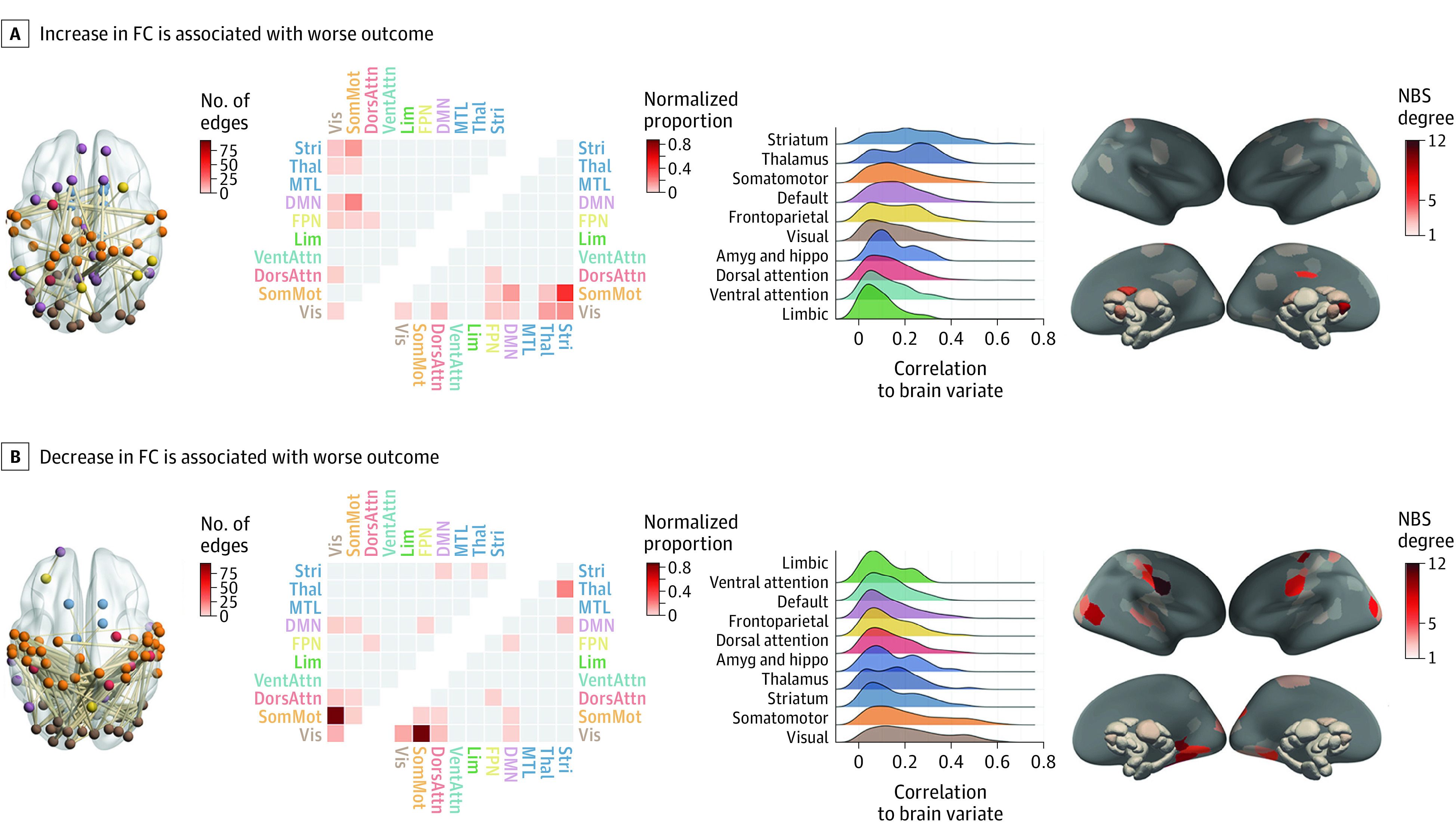
Association Between Short-term Functional Connectivity (FC) Changes and Behavioral Outcomes Panels depict the edges where change in FC was significantly correlated (false discovery rate [FDR]–corrected) with the brain change canonical variate. Each panel contains (from left to right) (1) a visualization of the edges within the Network Based Statistic (NBS) component that correlated with the brain change variate, with nodes colored by canonical network and weighted by the number of connections to which they are attached in the NBS subnetwork; (2) a heatmap of raw (upper triangle) and normalized (lower triangle) proportions of edges in NBS component that fall within each canonical network; (3) a ridge plot of the distribution of correlations between each edge of the NBS component and the brain variate in the canonical correlation analysis (CCA), separated by network and sorted by the median value; (4) surface renderings colored by the number of edges in the NBS subnetwork attached to each node (NBS degree). Amyg indicates amygdala; DMN, default mode network; DorsAttn, dorsal attention network; FPN, frontoparietal network; Hippo, hippocampus; Lim, cortical limbic network; MTL, medial-temporal lobe (amyg and hippo); SomMot, somatomotor network; Stri, striatum; Thal, thalamus; VentAttn, ventral attention network; Vis, visual network.

A canonical correlation analysis including 12 principal components each explaining greater than 2% of variance in ΔFC identified a single significant canonical variate linking FC changes to the primary trial outcome measures (eFigure 2 in Supplement 3) (FWE *P* = .005; *R* = 0.901). The canonical loadings indicate that this variate was strongly associated with reducing SOFAS over time (*r* = −0.99) and moderately associated with increasing BPRS (*r* = 0.49). A total of 179 edges showed significant correlations with the ΔFC variate (false discovery rate, threshold *P* < .05; only the edges in the network that survived *P* < .05 were retained), such that 59 edges correlated positively (0.32 < *r* < 0.66) (Figure 3A) and 120 edges correlated negatively (−0.41 < *r* < −0.65) (Figure 3B). Positively correlated edges (Figure 3A) largely implicated links between sensorimotor/visual and association and subcortical systems, particularly the striatum. Regions with the largest number of positively correlated edges were the right nucleus accumbens core, left posterior cingulate, and posterior caudate. Negatively correlated edges (Figure 3B) strongly implicated links between somatomotor and visual networks. Regions with the largest number of negatively correlated edges included bilateral precentral and postcentral gyrus, occipital pole, and lingual gyrus. Thus, across both PIPT and MIPT patients, worse clinical outcome was associated with increased FC between striatal and somatomotor regions, coupled with decreased FC between sensory and motor systems.

### Antipsychotic-Related Changes

#### Baseline to 3 Months

**Figure 4.**
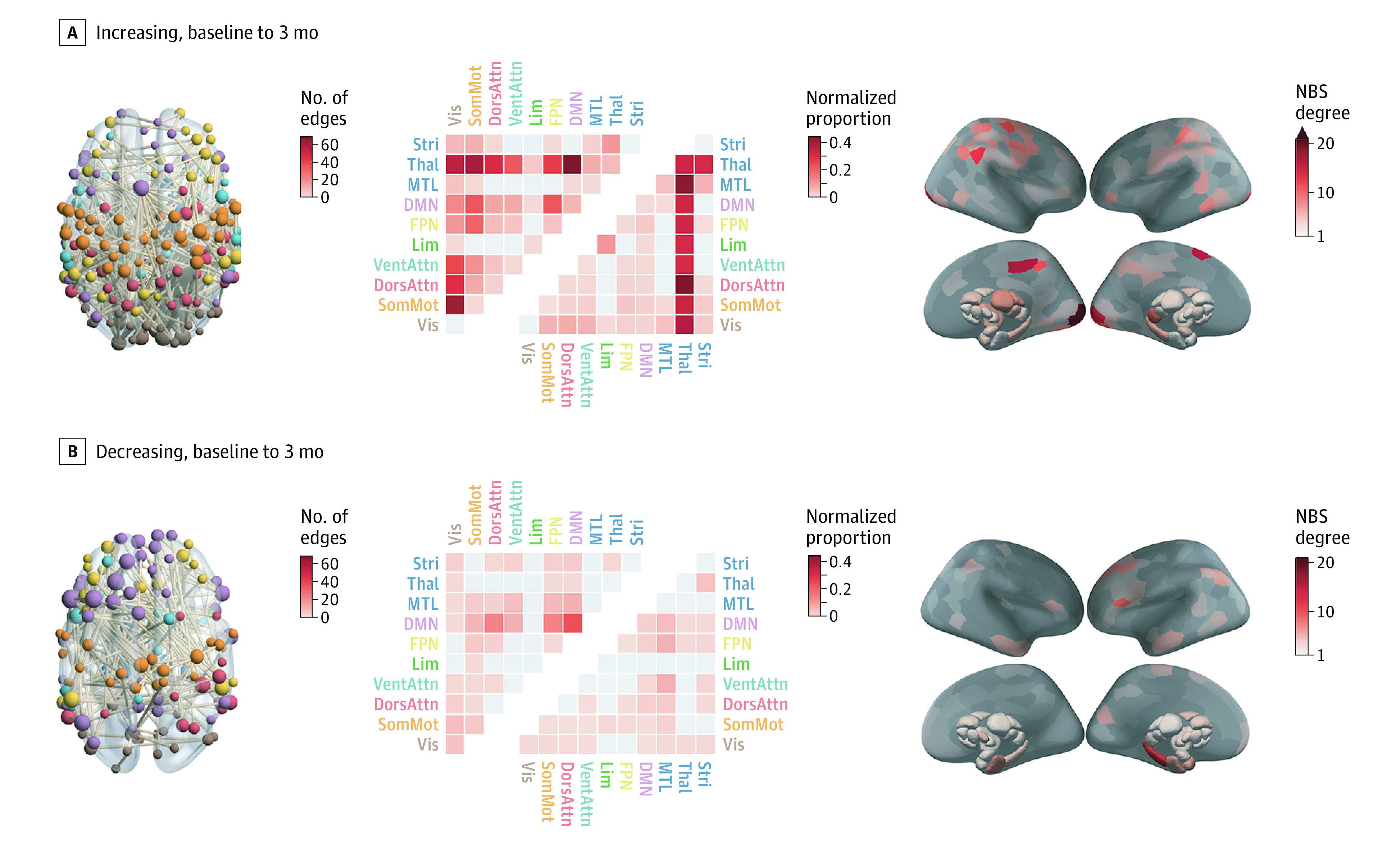
Longitudinal Functional Connectivity (FC) Changes Due to Antipsychotic (AP) Treatment Each panel shows changes from baseline to 3 months and contains (from left to right) (1) a visualization of the significant Network Based Statistic (NBS) subnetwork, with nodes colored by network and weighted by degree; (2) a heatmap of the proportion of edges within the NBS component that fall within each of the canonical networks, quantified using raw (upper triangle) and normalized (lower triangle) proportions; and (3) surface renderings depicted the number of edges in the NBS subnetwork attached to each brain region (NBS degree). DMN indicates default mode network; DorsAttn, dorsal attention network; FPN, frontoparietal network; Lim, cortical limbic network; MTL, medial-temporal lobe (amygdala and hippocampus); SomMot, somatomotor network; Stri, striatum; Thal, thalamus; VentAttn, ventral attention network; Vis, visual network.

We identified a single NBS component showing an altered FC trajectory in the MIPT group compared with the PIPT and control groups, comprising 634 edges and including all 316 regions (FWE *P* < .001) (Figure 4 and eFigure 6 in the Supplement). This network indicates that antipsychotic exposure is generally associated with more FC increases (446 edges) than decreases (188 edges) over time. The subnetwork showing increased FC over time strongly implicates the thalamus and its connections to all other networks, particularly when considering normalized counts (Figure 4A). Regionally, the right lingual gyrus, bilateral occipital pole, left superior frontal gyrus, and right precuneus cortex featured prominently in this subnetwork. Edges showing medication-related FC decreases are more diffusely spread across the networks (Figure 4B). Regionally, decreases predominantly involve the left posterior hippocampus and left inferior frontal gyrus. We found no significant associations between medication-related ΔFC and change in primary outcome measures at 3 months.

### Longer-term Illness-Related and Antipsychotic-Related Changes (12-Month Follow-up)

Long-term changes in FC in antipsychotic-naive patients were circumscribed to a relatively small subset of edges showing large effects concentrated predominantly in the default mode and somatomotor networks (eAppendixes 4-6 and eFigure 3 in Supplement 3). Long-term medication-related changes paralleled the 3-month results (eFigures 3 and 5 in Supplement 3), with more FC increases than decreases over time and a preferential involvement of the DMN and cortical limbic network. Notably, these networks showed lower FC in patients at baseline (Figure 2A).

## Discussion

This secondary analysis of a triple-blind, placebo-controlled, randomized clinical trial allowed us to disentangle longitudinal FC changes in antipsychotic-treated and antipsychotic-naive patients during the early stages of psychosis. We identified widespread FC changes in antipsychotic-naive patients within the first few months of an initial psychotic episode, involving both abnormally increasing and decreasing FC over time when compared with healthy controls, with evidence for a preferential involvement and recovery of default mode and cortical limbic systems in patients receiving psychosocial treatment and placebo. In contrast, antipsychotic-related changes predominantly involved increased FC between the thalamus and other brain networks. Across all patients, longitudinal change in FC over the first 3 months between subcortical, sensorimotor, and association networks was most strongly related to change in clinical outcome. We additionally found more circumscribed changes in antipsychotic-naive patients affecting medial parietal cortex over a 12-month period. Long-term antipsychotic effects were primarily associated with increased FC in cortical limbic and association systems.

### Baseline Connectivity Differences Between Antipsychotic-Naive Patients and Controls

Our baseline analysis identified a broadly distributed subnetwork of altered FC in antipsychotic-naive patients with FEP, involving an approximately equal proportion of edges showing hyperconnectivity and hypoconnectivity relative to controls. This broad distribution of FC differences is consistent with a general disconnection hypothesis of psychotic illness,^2,31,32^ in which symptoms are tied to a breakdown of interregional communication. Our results suggest that, from the outset of illness, the nature of this breakdown is both complex and widespread.

Within this affected subnetwork, FC reductions are predominantly concentrated within the DMN and its interactions with other systems. We also found evidence for preferential involvement of connectivity between the medial-temporal lobe (MTL), including both the amygdala and hippocampus, and cortical limbic systems with the rest of the brain, after accounting for differences in network size. Together, these findings point to a prominent role for dysconnectivity of default mode and cortical limbic systems in FEP. Given the presumed role of the DMN in self-referential and reflective processes^33,34^ and the cortical limbic and MTL systems in emotional function,^35^ our findings align with views that psychotic symptoms may arise from aberrant salience attributed to internal representations,^36^ as well as evidence for prominent impairments of emotional processing and expression in people with psychotic disorders.^37,38^ Moreover, our findings of hypoconnectivity across the DMN align with recent studies examining antipsychotic-naive patients.^12,39,40^ However, DMN dysfunction is a common finding in many different psychiatric disorders and may reflect a general vulnerability to psychopathology.^41^ At the regional level, the striatum and thalamus were attached to a large number of affected edges, which is consistent with previous research implicating the fronto-striato-thalamic circuits in the pathogenesis of psychosis^3,42,43,44^ and our previous investigation showing volumetric decline within the pallidum in patients in the PIPT group.^23^

### Connectivity Changes in Antipsychotic-Naive Patients

From baseline to 3 months, we also observed widespread FC changes in patients in the PIPT group relative to controls. The FC reductions were spread relatively evenly across networks, whereas FC increases were strongly concentrated on connections linking the DMN and cortical limbic system to the rest of the brain. Critically, some of these changes in FC, particularly those affecting links between sensory, subcortical, and association networks, were related to improved symptom ratings and functional outcomes over time.

The findings of increasing FC in the DMN and cortical limbic systems, together with the prominent reductions in these systems observed at baseline, suggest a normalization of FC over the first few months of illness. This recovery may be due to the placebo or psychosocial intervention.^24,45^ Although there is some evidence that such interventions can normalize FC across the limbic system and associated networks,^46^ previous research has not been able to rule out the effect of antipsychotic medication. Our findings suggest that either placebo administration or psychosocial intervention alone, or in combination, may be sufficient to normalize aberrant FC in these systems.

Our analysis of the 12-month time point found fewer and more circumscribed FC changes over time in the PIPT group relative to the control group, suggesting that the majority of FC changes occurred within the first 3 months of the study, when patients were most intensively engaged with the psychosocial therapy. However, definitively testing this hypothesis requires comparison with an additional treatment group receiving either basic support or no intervention. Such a design is difficult to justify ethically in patients who are unmedicated. Notably, our prior investigation of this cohort^23^ found that pallidal volume in patients receiving placebo normalized by the 12-month follow-up. In the current study, we see a normalization of altered FC in patients receiving placebo by 3 months, suggesting that normalization of function may precede structure.

The FC within the MTL was lower at baseline and did not show evidence for preferential increases over time, suggesting that reduced MTL FC may represent a core illness-related feature of psychosis that may not be modified by psychosocial treatment or placebo administration. This result aligns with patient studies^47^ and animal models^48^ suggesting a primary role for MTL areas in driving the onset of psychotic illness.

### Connectivity Changes in Antipsychotic-Treated Patients

Several studies have found that lowered FC at baseline is either normalized or partly normalized after antipsychotic treatment^11,18^ (although see also Röder et al^49^ and van Dellen et al^50^). Accordingly, we show that antipsychotic exposure largely increases FC and preferentially affects connections between the thalamus and the rest of the brain. The thalamus is a globally connected hub that relays multimodal information between diverse functional networks.^51^ The therapeutic efficacy of antipsychotics is primarily mediated by their antagonism of D2 receptors in the striatum,^52^ and dopamine dysregulation in psychosis is thought to disrupt striato-thalamic filtering of sensory and limbic information to the cortex.^53^ Our findings suggest that antipsychotics may partially remediate communication between the thalamus, striatum, and cortex, thereby normalizing information flow within widespread cortico-subcortical systems.

At 12 months, we found evidence for a prolonged increase of FC, mainly in the DMN and cortical limbic systems, in patients who are medicated. An early effect of antipsychotics on thalamocortical signaling may therefore be followed by a more sustained influence on the DMN and cortical limbic FC, which showed prominent illness-related differences at baseline and a partial recovery, correlated with symptom outcome, in the PIPT group. Normalizing communication between limbic/paralimbic systems and the rest of the brain may therefore be a primary therapeutic target in FEP.

### On the Generalizability of Our Findings

Our sample included patients with affective and nonaffective psychosis, given that antipsychotics are used in the treatment of both.^54,55^ Clinical and biological evidence supporting their nosological separation has been mixed,^56,57,58,59,60^ and our analysis presents a transdiagnostic investigation of the psychosis spectrum. Future work may seek to evaluate differences between distinct illness subtypes.

Our use of a placebo-controlled design required strict safety and ethical guidelines. To comply with these guidelines, patients were required to have low levels of suicidality and aggression, to have a duration of untreated psychosis of less than 6 months, and to be living in stable accommodations with social support. Additionally, patients who showed increased risk at any point or did not improve in clinical symptoms or functioning within 3 months of study intake were discontinued from the trial. These criteria resulted in removal of 17 patients by the 12-month time point.

### Limitations

Our study had some limitations. Our strict inclusion criteria raise concerns that our sample comprises patients with a mild form of illness. This seems unlikely, however, as the mean baseline BPRS score of patients in our study would classify them as markedly ill,^61^ and the mean baseline SOFAS score was consistent with serious functional impairment, being comparable to epidemiologically representative cohorts of patients with FEP (Table).^62^ It is possible, however, that patients who remained in the study, particularly at the 12-month time point, have a less deteriorating form of psychotic illness. As such, the apparent normalization of specific brain systems seen in patients in the PIPT group by this time point may not generalize to individuals with a more deteriorating form. By the same reasoning, it is also possible that the illness-related differences that we identified represent a conservative estimate of brain dysconnectivity. Future work may extend our approach to include more rigorous monitoring of patients with a higher risk and evaluate the extent to which our findings generalize to that FEP subgroup. We also note that, although rates of substance and tobacco use did not differ between the treatment groups, use of these substances was not controlled for in our analyses.

## Conclusions

Antipsychotic-naive patients with FEP showed widespread functional dysconnectivity at baseline, particularly in default mode and cortical limbic systems, with evidence for an improvement of these changes during the first 3 months of illness. Our results also suggest that antipsychotics may normalize dysconnectivity primarily by affecting thalamocortical and cortical limbic networks.

## Notes

Supplement 1.
Trial Protocol

Supplement 2.
MRI Protocol

Supplement 3. **eMethods 1.** Additional Details **eMethods 2.** Trial Safety Procedures **eMethods 3.** Antipsychotic and Concomitant Medication Details **eTable 1.** Cumulative Antipsychotic Exposure (olanzapine equivalents, mg) in Placebo Group (PIPT) and Medication Group (MIPT) **eTable 2.** Percentage of Each Treatment Group Included in Analyses Who Received Nonantipsychotic Psychotropic Medication During the Primary Treatment Period **eFigure 1.** Histogram of Cumulative Dose (olanzapine equivalents, mg) Exposure in the Placebo and Medication Groups Across the Three Time Points **eMethods 4.** MRI Acquisition Details **eMethods 5.** Image Processing and Quality Control **eMethods 6.** Further Details on Statistical Analysis **eMethods 7.** Demographic and Sample Characteristics **eAppendix 1.** Antipsychotic-Naïve Results (Baseline) **eAppendix 2.** Antipsychotic-Naïve Results (Baseline to 3 mo) **eAppendix 3.** Antipsychotic-Related Results (Baseline to 3 mo) **eAppendix 4.** Long-Term Changes in Antipsychotic-Naïve Patients Compared to Healthy Controls (Baseline to 12 mo) **eAppendix 5.** Long-Term Antipsychotic-Related Changes (Baseline to 12 mo) **eAppendix 6.** Association Between Long-Term Changes and Symptoms and Functioning **eFigure 2.** Canonical Correlation Analysis Between Short-Term Functional Connectivity Changes and Behavioral Outcomes **eFigure 3.** Longer-Term Effects in Antipsychotic-Naïve Patients (A, B) and Longer-Term Effects Related to Antipsychotic Medication (C,D) **eFigure 4.** Antipsychotic-Naïve and Antipsychotic-Treated Results at a Network Level (FWE *P* < .05) at Primary Thresholds of *P* < .01 and *P* < .001 **eFigure 5.** Baseline to 12 Months Antipsychotic-Naïve and Antipsychotic-Related Effects After Removing the 9 Subjects in the PIPT Group Who Were Exposed to Antipsychotics **eFigure 6.** Baseline to 3 Months Antipsychotic-Naïve and Antipsychotic-Related Effects After Adjusting for Nonantipsychotic Psychotropic Medication Use
eReferences

Supplement 4.
Data Sharing Statement
