# Supplementary material for: Functional Connectivity in Antipsychotic-Treated and Antipsychotic-Naive Patients With First-Episode Psychosis and Low Risk of Self-harm or Aggression: A Secondary Analysis of a Randomized Clinical Trial
Source: JAMA Psychiatry. 2021 Jun 23;78(9):1–11. doi: 10.1001/jamapsychiatry.2021.1422 (PMC8223142; doi:10.1001/jamapsychiatry.2021.1422)
Supplement: Supplement 1. — Trial Protocol [file jamapsychiatry-e211422-s001.pdf]

RESEARCH PROTOCOL
Version 11, 9 January 2018

A randomised placebo controlled trial of intensive psychosocial treatment plus or minus anti-psychotic medication for first episode psychosis with low-risk of self-harm or aggression. The STAGES Study: Staged Treatment and Acceptability Guidelines in Early Psychosis

Chief Investigators: Professor Patrick McGorry, Dr Shona Francey, Dr Barnaby Nelson, Dr Andrew Thompson, Professor Michael Berk, Susy Harrigan, Associate Professor Alex Fornito, Dr Brian O'Donoghue, Dr Kelly Allott, Dr Mario Alvarez-Jimenez

Co Investigators: Dr Craig Macneil, Dr Tina Proffitt, Kingsley Crisp, Lara Baldwin, Professor Christos Pantelis, Dr Aswin Ratheesh, Dr Andrea Polari, Jessica Graham, Sumudu Mallawaarachchi

Location of Study: Early Psychosis Prevention and Intervention Centre (EPPIC), a program of Orygen Youth Health

Sponsor: Orygen, The National Centre of Excellence in Youth Mental Health
35 Poplar Rd, Parkville VIC 3052

CONFIDENTIAL
Confidential information contained herein is made available to you in your capacity as Investigator. It is provided only for review by you, your staff, Institutional Review Board and/or Institutional Ethics Committee members, or regulatory authorities. Except as necessary to obtain properly informed consent for participation, it is expected that there will be no disclosure to other persons.

Table of Contents

1. INTRODUCTION 4

1.1. BACKGROUND 4

1.1.1. PHARMACOLOGICAL TREATMENT IN FEP 5

1.1.2. PSYCHOLOGICAL INTERVENTIONS IN SCHIZOPHRENIA AND FEP 6

1.1.3. EVIDENCE FROM STUDIES DELAYING INTRODUCTION OF NEUROLEPTICS 6

1.1.4. ETHICAL ISSUES 8

1.2. RATIONALE FOR THE CURRENT STUDY 9

1.2.1. AIMS OF THE CURRENT STUDY 10

1.2.2. HYPOTHESES 10

2. METHODOLOGY 11

2.1. RESEARCH PLAN 11

2.2. PARTICIPANTS 12

2.2.1. INCLUSION CRITERIA - FEP 13

2.2.3. EXCLUSION CRITERIA - FEP 13

2.2.5. WITHDRAWAL CRITERIA 14

2.3. DESIGN 15

2.3.1. RANDOMISATION DESIGN 15

TABLE 1. *RANDOMISATION LISTS FROM WHICH SUBJECTS WILL BE DRAWN* 16

2.3.2. SAMPLE SIZE CALCULATION 16

2.4. STATISTICAL ANALYSES 18

2.5. SAFETY MEASURES 19

2.5.1. DEFINITIONS 19

2.5.1.1. ADVERSE EVENT 19

2.5.1.2. SERIOUS ADVERSE EVENT 20

2.5.2 REPORTING SAES 20

2.6. INSTRUMENTS 21

2.6.1. DEMOGRAPHICS AND DIAGNOSTIC INFORMATION: 21

2.6.2. MONITORING OF MENTAL STATE AND RISK (WITHDRAWAL CRITERIA) 22

2.6.3. MONITORING OF SYMPTOMOLOGY 22
2.6.4. CLINICAL OUTCOME MEASURES 22
FEP PARTICIPANTS 22
2.6.5. QUALITY OF LIFE AND FUNCTIONING MEASURES 23
2.6.6. SUBSTANCE ABUSE MEASURES 23
FEP PARTICIPANTS 23
2.6.7. SAFETY ASSESSMENTS 23
FEP PARTICIPANTS 23
2.6.8. METABOLIC MONITORING 24
2.6.9. NEUROCOGNITIVE PROFILE 24
2.6.10. STRUCTURAL IMAGING 25
2.7. PROCEDURE 26
TABLE 2. *ASSESSMENT SCHEDULE* 28
2.7.1. STUDY WITHDRAWALS 32
3. TREATMENT MODALITIES 34
3.1. INTENSIVE PSYCHOSOCIAL TREATMENT PACKAGE 34
3.2. MEDICATION PROTOCOL 35
3.2.1. DRUGS, FORMULATIONS AND STRENGTHS 35
3.2.2. DOSAGES 35
3.2.3. MEDICATION COMPLIANCE 35
3.2.4. LABELLING, STORAGE AND ACCOUNTABILITY 36
3.2.5. BLINDING 36
3.3. CONCOMITANT THERAPY 36
4. OUTCOMES AND SIGNIFICANCE 37
5. DATA MANAGEMENT 38
6. ETHICAL CONSIDERATIONS 38
6.1. REGULATORY AND ETHICAL COMPLIANCE 39
7. GOVERNANCE AND FUNDING 40
8. REFERENCES 41

1. Introduction

1.1. Background

The last decade has seen a significant shift in psychiatric services around the world towards a greater focus on early intervention for psychotic illnesses. This development has been based on the notion that earliest possible detection and effective intervention for psychosis will deliver the best outcome for patients and result in the least burden for the health care system (Edwards et al., 2002; McGorry et al., 1996). Service reform has been effective in reducing the duration of untreated psychosis (DUP) experienced by those with first episode psychosis (FEP). This is shown by the significantly lower median DUP reported by specialist early psychosis services (e.g., Carbone et al., 1999; Linszen et al., 1998) compared to those reported for first episode cases seen in standard psychiatric services in the early 1990s (e.g. Loebel et al., 1992; Szymanski et al., 1996). This change has meant that people are receiving treatment for psychosis earlier in the course of their illness, which raises the question of what the most appropriate form of treatment is for people (very) early in the course of a first psychotic episode. It is likely that the influence of early detection programs associated with specialist early psychosis services has changed the composition of the first episode psychosis cohorts such that a change in treatment approach may be indicated.

It is well established that psychotic symptoms occur on a continuum throughout the general population (Johns & van Os, 2001; van Os, Hanssen, Bijl & Ravelli, 2000) and that many experience such symptoms without distress, lowered functioning or any discernable 'need for care' (Yung et al., 2006). This notion is supported by reports of high rates of psychotic like experiences reported in community studies (Yung 2007; Laurens 2007). The boundary, particularly at the earliest stages, between those who need and will benefit from treatment and those who will have a benign course without pharmacological intervention remains to be defined. There is also substantial diagnostic heterogeneity at this stage, and hence differential treatment requirements (Yung 2007). The twin possibilities that early intervention efforts result in some of these individuals being referred for treatment, and that shorter DUP may be associated with earlier remission (Loebel et al. 1992), less relapse (Crow et al. 1986) and better outcome (Marshall et al. 2005) suggest that more benign treatments may be the most appropriate first line interventions the earliest stages of the onset of

psychotic like features. This concept is central to the clinical staging model of psychiatric disorders, which posits that earlier treatments will be safer and more effective than those delivered later in the course of a disorder and may prevent progression to more severe forms of disorder (McGorry, Hickie, Yung, Pantelis, & Jackson, 2006). Overall, these emerging findings suggest that more benign treatments may be the most appropriate first line interventions for the earliest stages of the onset of psychotic like features, and that a staged approach to treatment choice may be optimal. Such interventions could consist of psychological treatments with demonstrated efficacy and no serious side effects, and would avoid the early introduction of neuroleptic medication. Anti-psychotic medications should then only be introduced as a second line treatment when more benign treatments have failed, or when a more aggressive course is evident.

1.1.1. Pharmacological treatment in FEP

Neuroleptic medications have been shown to be very effective for the treatment of acute psychosis; however, there are well-documented and sometimes serious side effects associated with these medications (Allison & Casey, 2001; Muench & Carey, 2001). The latter is underscored by evidence of the iatrogenic risks of atypical anti-psychotics in the short-term (Allison & Casey, 2001; Muench & Carey, 2001), possible morphological changes in the brain over the intermediate term (Corson, Nopoulos, Miller, Arndt, & Andreasen, 1999), and the poorly understood longer-term effects that may include tardive dyskinesia (Llorca, Chereau, Bayle, & Lancon, 2002). Agents that increase the risks of developing the metabolic syndrome contribute to a marked increase in long term cardiovascular risk, underscoring the necessity for cautious and individualised risk benefit analysis. On the other hand, other research has indicated that antipsychotic and other medications, notably lithium and SSRIs, may have neuroprotective effects (Berger et al., in press, Lieberman et al 2005). Coupled with the concerns over the safety of anti-psychotic medication is evidence that medication adherence is problematic. Coldham, Addington, and Addington (2002) found that approximately 60% of FEP patients at a specialised treatment service were not compliant or inadequately compliant with anti-psychotic medication, which is consistent with other reports (see Perkins, 1999; Oehl, Hummer & Fleischhacker, 2000). Moreover, the introduction of second-generation anti-psychotics has not improved adherence to medication (Kane & Malhotra, 2003).

1.1.2. Psychological interventions in schizophrenia and FEP

Recently, there has been increased interest in examining the effects of cognitive-behavioural treatment (CBT) for schizophrenia. There is evidence that CBT effects are greatest in the early stages of the illness. Morrison et al. (2004) and Bechdolf et al. (2007) reported that the conversion to FEP could be significantly reduced in at-risk samples by CBT. In the two trials that focused on FEP, the first found that CBT and supportive counselling had significant advantages over routine care (SoCRATES trial; Lewis et al, 2002) and the second demonstrated that Cognitively Oriented Psychotherapy for Early Psychosis (COPE) led to an improved adaptation to illness (Jackson et al, 2001). In a recent review of the clinical significance of CBT for the treatment of schizophrenia, consistent evidence was found that CBT reduces persistent positive symptoms in patients with chronic schizophrenia, and has modest effects in hastening recovery from acute episodes (Tarrier, 2005). There is also emerging evidence that CBT is likely to be beneficial for the FEP population (Tarrier, 2005). In a review of psychosocial interventions for FEP (Penn, Waldheter, Perkins, Mueser, & Lieberman, 2005), the authors found that CBT was effective in the treatment of auditory hallucinations (Lewis et al, 2002; Tarrier et al, 2004), decreased hopelessness (Power et al, 2003), increased adaptation to illness (Jackson et al, 2001), contributed to better treatment adherence (Haddock & Lewis, 2005), and that CBT and supportive counseling were both effective in reducing comorbid substance abuse (Edwards et al, 2006). Furthermore, emerging evidence reveals that the acceptability of individual CBT and family interventions for FEP patients is high (Haddock & Lewis, 2005). There is also evidence in bipolar disorder that the efficacy of psychotherapy is greatest at the earliest stages of the disorder, and declines substantially with increasing episode numbers, such that in individuals who have had over 12 episodes of illness, the effect of CBT switched to being detrimental (Scott et al 2006).

1.1.3. Evidence from studies delaying introduction of neuroleptics

As noted above, one of the most prominent reforms in public mental health care over the previous decade has been the emergent focus on early psychosis and the associated drive to provide rapid optimal treatment in order to prevent biological and psychosocial deterioration during the so-called “critical phase” or early years after onset (Birchwood, 2000). Within this paradigm it is accepted that

optimal treatment includes the early introduction of novel atypical neuroleptics at low doses in order to minimize DUP, which is thought to be associated with worse clinical and psychosocial outcome (Remington, Kapur & Zipursky, 1998; Wyatt & Henter, 2001). From this perspective, the "untreated" in DUP is synonymous with neuroleptic naïve. However, although there is now evidence for a small association between DUP and outcome in FEP (Marshall et al., 2005), it has not been established that anti-psychotic medication is an essential component of effective treatment for all cases of first episode psychosis. In a recent meta-analysis, Bola (2006) found a small, non-significant advantage for initially non-medicated groups with established schizophrenia over medicated groups in terms of improved outcomes. This finding may provide evidence that an initial period of non-medication produces superior outcomes to those who receive medication. At the very least, Bola's results indicate that there is no evidence that long-term harm is caused to psychotic patients by short-term medication-free periods. Further evidence is provided by Johnstone, Owens, Crowe and Davies' (1999) randomised controlled trial (RCT) of three anti-psychotic medications, plus a fourth placebo arm, with the aim of examining the potential effects of delaying anti-psychotic medication for a four-week period. At the 2.5-year follow-up, the occupational functioning, psychopathology, treatment needs and cognitive functioning of those who experienced the 4-week non-medication period was no different from those who were initially medicated. The authors concluded that a 4-week delay in the introduction of anti-psychotic medication does not produce long-term deleterious effects, compared to those who receive medication. Combined, these studies indicate little evidence that long-term harm results from a short-term delay in introducing anti-psychotic medication. Carpenter (1997) reviewed the data on medication free intervals in schizophrenia and concluded that there was no evidence of later harm from such intervals. This has led some researchers to advocate the provision of psychosocial interventions with short-term withholding of medication (see Bola, 2006b; Carpenter, Appelbaum and Levine, 2003). However, there is a paucity of quality research to date addressing the effects of delaying medication in schizophrenia, due to ethical concerns (Bola, 2006a). In a series of commentaries on the Bola paper, on the basis of the capacity to give informed consent and the lack of evidence of harm flowing from this approach, a number of eminent schizophrenia researchers have endorsed the notion that medication-free trials are ethical, even in patients with established illness, where it is established that medication plays a vital role in

treatment. They also emphasise that medication-free research is appropriate and especially illuminating in first episode samples at least for short periods (Schooler 2006).

1.1.4. Ethical issues

The basis for an ethical study of the efficacy of a psychosocial intervention in the absence of anti-psychotic medication in FEP lies in using multiple strategies to maximise the safety of participants and the need to investigate the assumption that all cases of FEP benefit from the early introduction of anti-psychotic medication. As argued above, in the era of early intervention, young people experiencing psychotic illnesses are receiving treatment much earlier in the course of their illness and it may be that more benign forms of treatment in the context of supportive psychotherapeutic relationships and close monitoring may be sufficient to enable satisfactory recovery without the risks and noxious side effects caused by even the most modern anti-psychotic medications (see McGorry et al, 2006). In consort, the epidemiological data that there are high rates of psychotic like experiences in community samples that are not associated with significant distress or impairment supports the systematic staged use of interventions concordant with clinical evolution and need.

In a discussion of ethical approaches to clinical trials in schizophrenia in the light of the 2002 Clarification of the 2000 Declaration of Helsinki, Carpenter, Appelbaum and Levine (2003) list five criteria that research plans must satisfy in order to meet the ethical standards set by the Clarification. Specifically, these criteria include:

1) the likelihood that the intervention being tested has a clinical advantage over existing treatments,
2) the application of careful informed consent procedures,
3) the presence of compelling reasons for placebo use,
4) the selection of participants that minimises potential serious adverse consequences, and
5) a risk-versus-benefit analysis that favours the advantages from placebo use over the risk to participants.

The rationale and research plan of the current study will be outlined in the following sections, before detailing how these criteria will be addressed.

1.2. Rationale for the current study

The service reform focusing on early intervention for psychotic illnesses has been effective in reducing the duration of untreated psychosis (DUP) experienced by those with first episode psychosis (FEP). This has meant that essential components of efficacious treatment for early psychosis need to be rethought and to be further explored in clinical trials.

On the one hand psychological interventions have been demonstrated to be effective in reducing level of symptoms, number of psychotic episodes and in improving functioning, and greater knowledge of the potential harm of neuroleptic treatment has become available. Combined, this suggests the value in reducing the number of young people with FEP treated with neuroleptics. On the other hand, with the potential neuroprotective components of pharmacological treatments and the potential harm of longer DUP, withholding medication may further negatively affect the early course of psychosis in young people with FEP.

Importantly, clinical trials focusing on exploring the essential components of FEP treatment can only be conducted in an ethical, safe, and feasible manner within a well-established early intervention service and by applying strict inclusion and withdrawal criteria for selecting 'low-risk' participants. The Early Psychosis Prevention and Intervention Centre (EPPIC) of ORYGEN Youth Health is a well-established specialised early psychosis service that incorporates a 24-hour crisis and assessment team (YAT), clinicians with considerable experience with the client group, and a good track record for research in early psychosis.

The current study is designed to investigate whether intensive psychosocial treatment and close monitoring in a specialized early intervention service constitutes an effective treatment of FEP in a selected subgroup of young people with FEP. The study will allow investigation of the acceptability, feasibility, safety and potential harm of withholding neuroleptic medication from a selected subgroup of young people with FEP who are judged to be at low risk and able to be managed conservatively, but closely monitored, in the community.

1.2.1. Aims of the current study

1. To compare 6 month functioning (SOFAS score) between the anti-psychotic-medication-free (placebo) intensive psychosocial treatment group (PIPT) and the medication plus psychosocial treatment group who will receive early treatment with anti-psychotic medication (MIPT).[1]
2. To examine the acceptability, feasibility and safety of delaying anti-psychotic medication for up to 6 months from carefully selected low-risk young people with FEP who are receiving intensive psychosocial treatment from a specialised FEP service. This examination will also look at the safety and tolerability of the medication that is received.
3. To investigate whether a subgroup of FEP patients will achieve satisfactory functioning, symptom reduction and remission following intensive psychosocial treatment in the absence of anti-psychotic medication (long drug naive time period but with intensive psychosocial treatment).
4. To identify predictors of success and failure in recovery from FEP with intensive psychosocial treatment in the absence of anti-psychotic medication.
5. To investigate whether the experimental prolonging of DUP (with regards to commencement of antipsychotic medication) contributes to variation in outcome.
6. To compare functioning (SOFAS score) between PIPT and MIPT at 12 and 24 months post study entry.
7. To compare symptomatology (BPRS score) between PIPT and MIPT at 6, 12 and 24 months post study entry.
8. To compare remission rates between PIPT and MIPT at 6, 12 and 24 months post study entry and recovery rates at 12 and 24 months post study entry.

1.2.2. Hypotheses

The hypothesis is that individuals assigned to intensive psychosocial treatment alone (PIPT) will be no worse on 6-month SOFAS functioning than individuals assigned to intensive psychosocial treatment plus anti-psychotic medication (MIPT) by more than a specified margin of equivalence.

[1] The sample size calculation is powered on this aim.

2. Methodology

2.1. Research Plan

Young people accepted into the EPPIC program will be screened for suitability for the current study on the basis of meeting predefined study inclusion criteria indicating low risk and adequate support outside the service (family or other social support). Prior studies in this population have shown that many young people experiencing FEP are still living at home and are dependent on their family, highlighting the potential of the family to provide resources and support (Malla et al, 2005). After informed consent has been obtained, participants will be randomly allocated to either the MIPT or PIPT group.

The intensive psychosocial treatment will incorporate close monitoring of mental state, intensive case management, cognitive behaviour therapy for psychotic and other symptoms, and family education and support (see below for details). For the PIPT group, this treatment will occur in the absence of anti-psychotic medication for 6 months unless they meet study withdrawal criteria (i.e., marked worsening of symptoms, increase in suicidality or aggressiveness, breakdown of family support). All study participants will be placed 'on alert' with YAT for the duration of the study to ensure that a rapid crisis response and implementation of established crisis plans occurs should this be required. Study Investigators who are all experienced clinicians will be available on roster for after hours emergency consultation regarding any of the study participants. All participants will be asked to provide written informed consent for participation in the study. Ethics approval for the study has been obtained from Melbourne Health's Office for Research. At the conclusion of the 6-month study period all participants will receive a comprehensive assessment and it may be that some from the placebo group are then commenced on anti-psychotic medication because although they did not meet study withdrawal criteria, low level symptoms or poor functioning indicate a need for stronger treatment.

Assessment of symptoms and functioning will be conducted at intake, 6 weeks, 3, 6, 12, and 24 months. At the conclusion of the study, the MIPT and PIPT groups will be compared on measures of functional outcome and acceptability of treatment received. Due to stringent inclusion and exclusion

criteria, recruitment to the study has been slower than anticipated. Consequently, the sample size and power calculations have been revised. Based on revised estimates the study is realistically expected to recruit approximately95 participants over a 5 - year period.

2.2. Participants

Young people accepted for treatment within EPPIC by the intake team (YAT) will be systematically screened for suitability for the study (low risk and aggression as assessed using the BPRS, see below) on the basis of meeting predefined study criteria indicating low risk (see below). Eligibility for EPPIC is defined as follows: Residing in Western Metropolitan Melbourne, aged 15 – 24 (up to 25th birthday), experiencing a first episode of psychosis, up to 7 days treatment with neuroleptic medication for the current episode or a lifetime maximum dose of antipsychotic medication equivalent to 1750mg of chlorpromazine. Operational criteria for psychosis are defined as at least one of the following symptoms measured on the expanded Brief Psychiatric Rating Scale (exBPRS, which is the 24-items rated 1 (not present) to 7 (extremely severe) version, Ventura, Green, Shaner & Liberman, 1993) present on a daily basis for at least one week:
exBPRS Item 9. Suspiciousness (= 5)
exBPRS Item 10. Hallucinations (= 5)
exBPRS Item 11. Unusual thought content (= 5)
exBPRS Item 15. Conceptual Disorganization (= 4)
In addition, participants must meet DSM-IV diagnostic criteria for one of schizophreniform psychosis, schizophrenia, schizoaffective disorder, delusional disorder, brief psychosis, major depressive disorder with psychotic features or psychosis NOS.
Reasons for non-participation will be recorded to assess the representativeness of the sample.

Approximately 43 healthy young people who are matched for age, gender and socioeconomic status to the participants with FEP will also be recruited for the purposes of forming a healthy control group.

2.2.1. Inclusion Criteria - FEP

- Ability to give informed consent (Capacity to give informed consent will be assessed by an OYH doctor in cases where an intellectual disability is suspected)

  Where participants are of legal childhood age (i.e., under 18 years), consent will also be obtained from one of the participant's parents/legal guardian. Both the parent/legal guardian and participant will be required to sign the consent form in such a case. It will be the investigator's responsibility to determine whether a participant of legal childhood age has the capacity to consent to the study.

- Adequate comprehension of English to enable study assessments to be conducted
- Low suicidality (as defined by a score of < 5 on the exBPRS Suicidality subscale, i.e., no more than occasional suicidal thoughts with no plan or intent)
- Low aggressiveness (as defined by a score of < 5 on the exBPRS Hostility scale, i.e., may have been angry or yelled on several occasions but has not threatened people or thrown objects) and
- Patients must be currently living in stable accommodation, in regular contact with people who support the young person's participation in the study.

2.2.2. Inclusion Criteria - Controls

- 15 – 24 years of age (up to 25th birthday)
- Capacity to give informed consent

  Where participants are of legal childhood age (i.e., under 18 years), consent will also be obtained from one of the participant's parents/legal guardian. Both the parent/legal guardian and participant will be required to sign the consent form in such a case. It will be the investigator's responsibility to determine whether a participant of legal childhood age has the capacity to consent to the study.

- Adequate comprehension of English to enable study assessments to be conducted

2.2.3. Exclusion Criteria - FEP

- DUP > 6 months

- previous treatment with antipsychotic medication beyond the allowed dose (refer to p. 29)
- previous treatment with lithium or anticonvulsant medication for manic episode
  current pregnancy

2.2.4. Exclusion Criteria – Controls

- Past or current history of psychotic disorder
- Current mental illness
- History of childhood trauma
- Neurological conditions
- Previous head injury, resulting in loss of consciousness

2.2.5. Withdrawal Criteria

In order to ensure the safety of the participants and to minimise any psychosocial damage from prolonged psychosis, strict withdrawal criteria, in terms of either worsening risk or mental state or failure to achieve satisfactory recovery (or both) will be used in this study. Worsening risk or mental state is operationally defined as any of:

- An increase in suicidality as defined by a score of 5 or more on the exBPRS, Suicidality subscale (i.e., many fantasies about suicide, specific suicide plan, non-lethal attempt) that is maintained for one week
- An increase in aggression as defined by a score of 5 or more on the BPRS Hostility subscale that is maintained for one week
- A significant increase in severity of positive psychotic symptoms (from baseline level assessed at entry to the study) maintained for at least 7 days, operationally defined as at least a 2-point increase on the BPRS subscale of conceptual disorganisation, hallucinations, suspiciousness, or unusual thought content, that is not due to substance use
- A significant decrease in overall functioning as defined by a 20-point drop in SOFAS score from the baseline score, maintained for one month
- Request by the participant for the introduction of antipsychotic medication due to distress associated with ongoing psychotic symptoms (i.e., withdrawal of consent to medication protocol of the research study)
- Becoming pregnant

Failure to achieve satisfactory recovery is defined as the persistence of severe psychotic symptoms 3 months after study entry. This is operationally defined as a score of 5 or more on the exBPRS hallucinations (i.e., daily hallucinations), unusual thought content (i.e., definite delusions with some preoccupation), or suspiciousness subscales (i.e., frequent paranoid interpretation of events and preoccupation), or a score of 4 or more on the exBPRS conceptual disorganisation subscale (i.e., speech difficult to understand, definite example of incoherence).

2.3. Design

This will be a randomised placebo-controlled trial (RCT) designed to investigate the effectiveness of intensive psychosocial intervention in the absence of anti-psychotic medication in the treatment of selected low-risk cases of FEP. Outcome will be assessed by comparing the two study groups in terms of both clinical recovery from psychotic symptoms and level of functioning demonstrated at follow-up assessment. In addition, the acceptability, feasibility and safety of withholding neuroleptic medication from young people with FEP who are receiving intensive psychosocial treatment from a specialised FEP service will be examined. All study participants will receive the intensive psychosocial intervention package that incorporates intensive case management, close monitoring of mental state, cognitive behavioural therapy and family support and education.

The 2 groups will be as follows:

1. Medication plus intensive psychosocial treatment (MIPT)
2. Placebo plus intensive psychosocial treatment (PIPT)

2.3.1. Randomisation design

A stratified randomisation design will be used to allocate subjects to either the PIPT or MIPT treatment groups. It has been established that DUP and gender are associated with functional outcome, the key outcome measure in this study. Hence, it is proposed to incorporate these characteristics as stratifying factors in the randomisation, since any chance imbalances on these prognostic variables may bias the analysis. DUP will be included as a three-level factor; < 1 month; 1 month to < 3 months, 3 months to 6 months. Along with gender, this will result in six separate

randomisation lists from which subjects will be drawn (see Table 1 below). Subjects will be allocated to either the PIPT or MIPT treatment groups using randomly permuted blocks within each stratum, to ensure that subject allocation to the treatment groups is approximately equal.

Table 1. *Randomisation lists from which subjects will be drawn*

	DUP		
	< 1 month	1 to < 3 months	3 – 6 months
Male			
Female			

2.3.2. Sample size calculation

The early psychosis field stands to gain a great deal of knowledge by studying the magnitude of treatment effects, which may act as a basis of future larger scale studies. Therefore, many of the statistical outcomes will be reported descriptively in terms of effect sizes and clinically significant levels of change.

One of the key aims of this study is to show that those individuals assigned to psychosocial treatment alone (PIPT), and who subsequently need to be withdrawn are not harmed, relative to the medicated (MIPT) group. The essence of this research is that of a one-sided non-inferiority trial, which aims to show that an experimental treatment is no less effective than an active control treatment – it may be more effective or it may have a similar effect. Since we are interested in showing that psychosocial treatment alone is no worse than treatment with antipsychotic medication, and that any difference between the groups, up to a specified magnitude, is of no clinical or practical importance, standard power calculations based on the conventional null hypothesis testing framework are unsuitable to determine the required sample size. Instead, the power analysis is based on the equivalence testing framework, where the goal is to rule out any differences of clinical importance between the two treatments being compared.

The SOFAS was chosen as the primary measure used to determine the appropriate sample size, as it represents the level of functioning, an important feature in patients with a psychotic disorder. The

fundamental question is whether the 6-month group outcomes on this measure differ sufficiently to be deemed as clinically important. We have carefully considered the range of treatment effects which could be considered as clinically trivial. Initially, a difference of up to 8 points on the SOFAS was proposed as constituting functional equivalence between the two treatment groups. This non-inferiority margin has now been revised in accordance with recommendations from the non-inferiority trial literature that a less stringent consensus non-inferiority limit be developed that is both clinically relevant and statistically feasible (Ware & Antman, 1997; Kaul & Diamond, 2006). Such an alternative has been proposed by Snapinn (2000). Based on Snapinn's method, the revised non-inferiority margin (δ) is set at 10.5 SOFAS points. Further details regarding the calculation and selection of this non-inferiority margin are available on request. This adoption of this method helps ensure the viability of the non-inferiority margin as the smallest value that represents a clinically important effect, as advocated in the literature on non-inferiority trials (for example, Piagio et al, 2006; Snapinn, 2000). An improvement of any magnitude which favours the PIPT (psychosocial treatment only) group also fits within the definition of non-inferiority. Conversely, any difference favouring the MIPT (initially medicated) group in excess of the specified difference of 10.5 SOFAS points will be regarded as providing clinically important evidence of the inferiority of psychosocial-only (PIPT) treatment.

A power calculation was performed based on an equivalence testing framework using SamplePower (version 2.0). The sample size calculation is powered on an analysis which compares the 6-month SOFAS scores of the PIPT and MIPT groups. It was determined that 30 subjects per group are required to result in power of 80% to show that the treatment means are equivalent, assuming that a 10.5 point difference or less is considered clinically unimportant and with alpha set at 0.05 (1-tailed test).

2.3.3. Sample size calculation - Controls

To attain as accurate an estimate of sample size as possible for the recruitment of healthy controls, power estimates are based on an equivalence testing framework. Given that a substudy within this research project intends to investigate the relationship between childhood trauma and cognition in participants with FEP, the calculation is powered on an analysis which compares cognition scores of FEP patients with a history of childhood trauma to those with FEP and no childhood trauma to those

of healthy controls (Aas, Dazzan, Fisher, et al., 2011). It was determined that 43 healthy control participants are required to achieve power of 80%, with alpha set at 0.05.

2.4. Statistical Analyses

1. The primary analysis examines the question of non-inferiority of the PIPT treatment. This will be assessed by using one-sided analyses, including the one-sided confidence interval approach. It is recommended that in non-inferiority trials, both intention-to-treat and per protocol analysis should be conducted to ensure that the findings are robust under both conditions (Snapinn, 2000).

2. The acceptability, feasibility and safety of withholding antipsychotic medication for up to 6 months in low-risk individuals will be examined using a range of descriptive and inferential analyses. Acceptability to patients will be assessed by examining the number of PIPT subjects who commence medication or drop out of the study compared with the number of MIPT subjects who cease medication or drop out of the study. The feasibility of PIPT treatment will be examined by assessing the number of patients who make it to the end of the 6-month trial without requiring medication. Safety aspects will be evaluated by comparing a range of outcomes between the PIPT and MIPT groups, including number of critical incidents and the number of times it was necessary to invoke the Mental Health Act.

3(a). The characteristics of the sub-group of patients who achieve satisfactory recovery following psychosocial treatment in the absence of medication will be investigated using logistic regression. This analysis will focus solely on the subjects assigned to the psychosocial intervention (PIPT); the comparison group will be the PIPT subjects who require medication prior to the end of the trial. Predictors will include severity of baseline symptoms, initial DUP, gender, premorbid adjustment, Axis II diagnosis and substance use, amongst others.

3(b). A slightly different question relates to the predictors of survival time in psychosocial only treatment, or put another way, what predicts time to commencement of medication. Again, this analysis focuses solely on the PIPT group. Kaplan-Meier survival analysis and Cox regression will be used to examine predictors of survival time in the PIPT treatment condition. Survival techniques

offer the advantage of accommodating different lengths of follow-up and taking into account censored data. Predictors will be similar to those used in the logistic regression analysis.

4. The psychopathology and demographic profile of those who achieve recovery will be assessed by comparing three outcome groups; (A) those who recover without antipsychotics; (B) those who recover with the help of antipsychotics, and (C) those who don't recover even with antipsychotic treatment. ANOVA and chi-square tests of significance will be used to assess differences between these three groups on a range of baseline symptom and sociodemographic variables. *A priori* specified contrasts will compare these groups;

- Combined recovered groups (A,B) vs unrecovered group (C);
- Recovered group (A) vs recovered group (B);
- Recovered group (A) vs unrecovered group (C)

5. The association between the duration of treated psychosis (DTP) in the PIPT group and outcome at 6, 12 and 24 months will be assessed using linear regression models, and will covary for duration of untreated psychosis (DUP).

6. A comparison the PIPT and MIPT groups of level of functioning, level of symptomatology and rates of remission at each of the follow-up assessments will be undertaken to estimate the magnitude of effects in operation.

2.5. Safety Measures

The definitions of adverse events (AEs) and serious adverse events (SAEs) are given below. These will be recorded for FEP participants.

2.5.1. Definitions

2.5.1.1. Adverse event

An adverse event is the development of an undesirable medical condition or the deterioration of a pre-existing medical condition following or during exposure to a pharmaceutical product, whether or

not considered causally related to the product. In this study, any undesirable medical condition occurring from the time of signing consent (even if no study treatment or pharmaceutical product has been administered) will be considered to constitute an adverse event.

Adverse events will be recorded using the UKU side effect rating scale.

2.5.1.2. Serious adverse event

A serious adverse event is an AE that fulfils one or more of the following criteria:

- results in death
- is immediately life-threatening
- requires in-patient hospitalization or prolongation of existing hospitalization
- results in persistent or significant disability or incapacity
- is a congenital abnormality or birth defect
- is an important medical event that may jeopardize the patient or may require medical intervention to prevent one of the outcomes listed above.

2.5.2 Reporting SAEs

SAEs will be reported to regulatory authorities by the principal investigator in accordance with regulations. Further information related to reporting SAEs will be provided in the STAGES procedures manual.

Serious Adverse Events (SAEs) due to underlying illness.

For the purposes of the STAGES study, given the population of young people with FEP, SAEs which fulfil the SAE criteria however are expected to be due to the patient's underlying illness, are NOT required to be reported in an expedited manner.

These events are still required to be recorded on SAE forms for participant safety, data recording and tracking purposes, however, reporting to the Sponsor can be performed in a bulk manner on a regular basis (eg. quarterly). Examples of such SAEs in this participant population include admissions for participant protection (increase in suicidality), etc.

All other unexpected and expected SAE's MUST be reported to the Sponsor (Orygen, The National Centre of Excellence in Youth Mental Health) within 24 hours of becoming aware of the event regardless of the relationship to the investigational product.

2.6. Instruments

2.6.1. Demographics and Diagnostic Information:

FEP Participants

- Age, sex, education, marital status, occupation. Assessed at baseline, 3, 6, 12 and 24 months
- The Structured Clinical Interview for DSM-IV for Axis I disorders (SCID-I). The SCID-I is a structured interview based on the DSM-IV and will be used to determine Axis I diagnoses in FEP patients according to DSM-IV criteria. Assessed at baseline
- The Structured Clinical Interview for DSM-IV Axis II Personality Disorders (SCID-II) to determine any Axis II diagnoses. Assessed at 12 weeks for FEP participants
- Premorbid Adjustment Scale (PAS, Canon-Spoor et al. 1982). Assessed at 6 weeks for FEP participants
- Childhood Trauma Questionnaire (CTQ). Assessed at baseline for FEP participants by the treating clinician
- General Trauma Questions (Creamer et al., 2001). Assessed at baseline for FEP participants
- Nottingham Onset Schedule (NOS, Singh et al. 2005). Assessed at 6 weeks for FEP participants
- Medical and Psychiatric history. Assessed at baseline, 3,6,12 and 24 months for FEP participants
- Family History Index (FHI). Assessed at baseline

Control Participants

- Age, sex, education, marital status, occupation. Assessed at baseline, 6, 12 and 24 months

- Childhood Trauma Questionnaire (CTQ). Assessed as part of the screening procedure for control participants
- Medical and Psychiatric history. Assessed at baseline,6, 12 and 24 months
- Family History Index (FHI). Assessed at baseline
- Structured Clinical Interview for DSM-IV-TR Axis I Disorders, Research Version, Non-Patient Edition (SCID-I/NP, First, et al., 2002). Assessed as part of the screening procedure for control participants

2.6.2. Monitoring of Mental State and Risk (withdrawal criteria)

FEP Participants

Weekly throughout treatment phase, then at 12 and 24 months for FEP participants:

- Expanded Brief Psychiatric Rating Scale version 4 (ExBPRSvs.4)
- Social and Occupational Functioning Scale (SOFAS)

Control Participants

- Expanded Brief Psychiatric Rating Scale version 4 (ExBPRSvs.4)
- Social and Occupational Functioning Scale (SOFAS). Baseline, 6 12 and 24 months

2.6.3. Monitoring of symptomology

Baseline, 6 weeks (SANS only), 3, 6, 12 and 24 months for FEP participants:

- Scale for the Assessment of Negative Symptoms (SANS: Andreasen,1983)
- Hamilton Rating Scale for Depression (HAM-D: Hamilton, 1960)
- Hamilton Rating Scale for Anxiety (HAM-A: Hamilton 1959)

2.6.4. Clinical outcome measures

FEP Participants

- The UKU Side Effects Rating Scale (UKU: Lingjaerde et al, 1987). Baseline, first 4 weeks, then at 3, 6, 12, and 24 months.
- Clinical Global Impression Scale-Severity (CGI). Baseline, 6 weeks, 3, 6, 12, and 24 months.

2.6.5. Quality of life and functioning measures

<u>FEP Participants</u>

Baseline, 6, 12, and 24 months

- Heinrich Quality of Life Scale (QLS: Heinrichs, Hanlon, et al.,1984)
- World Health Organisation Quality of Life Assessment (WHOQOL-BREF, WHOQOL Group, 1998)

<u>Control Participants</u>

- Heinrich Quality of Life Scale (QLS: Heinrichs, Hanlon, et al.,1984). Baseline, 6, 12 and 24 months
- World Health Organisation Quality of Life Assessment (WHOQOL-BREF, WHOQOL Group, 1998). Baseline, 6, 12 and 24 months

2.6.6. Substance Abuse Measures

<u>FEP Participants</u>

Baseline then at 3, 6, 12 and 24 months

- World Health Organisation Alcohol Smoking & Substance Involvement Screening Test (WHO ASSIST, WHO ASSIST Working Group, 2002)

2.6.7. Safety Assessments

<u>FEP Participants</u>

3, 6, 12 and 24 months

- Admission to inpatient ward

2.6.8. Metabolic Monitoring

<u>FEP Participants</u>

Baseline, 3, 6, 12 and 24 months

- Height and weight to estimate BMI
- Systolic and diastolic blood pressure
- Waist and hip circumference to estimate ratio
- Blood glucose (random if can't get fasting)
- Total cholesterol
- Low and high density lipoprotein
- Triglycerides

<u>Control Participants</u>

Baseline, 6, 12 and 24 months

- Height and weight to estimate BMI
- Systolic and diastolic blood pressure
- Waist and hip circumference to estimate ratio

2.6.9. Neurocognitive profile

<u>FEP participants</u> - Baseline, 6, 12 and 24 months

<u>Control participants</u> - Baseline, 6 months, 12 and 24 months The neurocognitive test battery is comprised of paper and pencil measures of reading ability (as a proxy for premorbid ability), general intelligence, verbal immediate attention span and working memory, verbal relational memory (i.e., paired associate learning), verbal fluency, and response inhibition. The tests were selected based on the information available regarding validity and reliability, potential clinical utility, and practicality and tolerability. The entire battery will take approximately 35 minutes to administer (depending on subject factors such as ability and illness severity). The tests chosen for inclusion in the battery specifically include: 1) those on which first-episode or early psychosis/schizophrenia patients and controls have

been shown to differ significantly, and 2) those which are purported to specifically tap into prefrontal and medial temporal (especially left hippocampal) functioning.

- *Wide Range Achievement Test (Fourth Edition), Reading Test (WRAT-4).* The WRAT-4 is a graded, oral reading test that examines word recognition and pronunciation.
- *Wechsler Adult Intelligence Scale – 3rd Edition (WAIS-III)*. The Information & Picture Completion two-subtest short form of the WAIS-III will be used to provide a measure of the current intellectual functioning of the participants. The digit-span subtest will also be administered (taking approximately 3 minutes) to assess immediate verbal attention span and working memory.
- *The Stroop Test (Golden Version).* This test assesses the ease with which a subject can shift his or her perceptual set to incorporate changing demands and suppress a habitual response in favour of a less typical one.
- *Melbourne Relational Learning Task (MelRel).* The MelRel is a novel verbal associative learning task previously demonstrated to be sensitive to dysfunction in medial temporal lobe structures and to load minimally on attention and working memory.
- *The Controlled Oral Word Association Test (COWAT).* This test provides a measure of speed and efficiency of lexical search, and verbal generativity (verbal association fluency).
- *Wechsler Adult Intelligence Scale – 3rd Edition (WAIS-III)*. The Digit Symbol – Coding subtest will be administered to assess the speed of information processing. The test takes approximately two minutes to administer.

2.6.10. Structural Imaging

Consenting participants will undergo 3 Tesla magnetic resonance imaging (MRI) at the Royal Children's Hospital at baseline, three and 12 month follow-ups, allowing us to characterise both short and long-term effects of differences in treatment strategies on brain structure and function. Participants will be required to lie in the scanner for approximately 60 minutes. This is a fairly standard length of time that we have used extensively in previous studies of clinical patients and which we have found is well tolerated. During this time, various scans will be acquired to measure different aspects of brain structure, function and chemistry. Structure will be assessed using T1-weighted and diffusion-weighted imaging. Function will be assessed using T2*-star weighted

imaging of brain activity during a simple resting-state, and during performance of memory and emotion-processing tasks. Chemistry will be assessed using voxel-based magnetic resonance spectroscopy. The measures derived using these techniques will enable detailed assessment of how treatment impacts the brain. Where possible, participants will be introduced to the scanning environment via an on-site mock scanner. For a summary of assessments, please see Table 2.

2.6.11 Medication Monitoring – FEP participants

- Concomitant medication. Assessed at baseline, 3, 6, 12 and 24 months.
- Medication Adherence Rating Scale (MARS). Assessed at 4, 8, 12, 16, 20 and 26 weeks.

2.7. Procedure

2.7.1. FEP Participants

After providing informed consent to involvement in the study, participants will be allocated to a treatment team comprising a Consultant Psychiatrist and a Case Manager. An additional Family worker will provide family-oriented intervention and support as required. The treating team will meet frequently with the participant especially in the early phases of engagement and treatment, in order to monitor mental state and check for signs of deterioration or increasing risks, with at least twice weekly face-to-face appointments supplemented by phone contact as required. Emergency response systems will be put in place for each participant including crisis plans, YAT notification and intervention and rostered Chief Investigator backup if deemed appropriate by the treating team as described previously. Intensive (at least weekly) psychosocial treatment will continue for the 6-month duration of the trial, unless the participant is withdrawn from the trial due to worsening illness, failure to achieve satisfactory recovery or if withdrawn from the medication arm of the trial as operationally defined below. A long term follow-up visit 2 years after trial completion is planned, in order to determine if there is any enduring differences between group clinical, neuropsychological or neuroimaging sequelae of this staged treatment approach.

Medication

Medication (MIPT) group: Participants randomised to this arm of the trial will be prescribed anti-psychotic medication by the treating Consultant Psychiatrist and will also be prescribed other psychotropic medication (e.g., antidepressant, anxiolytics) if warranted.
Placebo (PIPT) group: Although inclusion in the placebo group of this RCT precludes the use of anti-psychotic medication, other psychotropic medication as considered appropriate for individual study participants by the treating Consultant Psychiatrist (e.g., antidepressants, anxiolytics) will be permitted but will be recorded for later inclusion in analyses.

2.7.2. Controls

Prior to consenting to participate in the research study, screening questionnaires will be administered, asking about experiences of childhood trauma in addition to any psychiatric symptoms previously or currently experienced. These questionnaires consist of the Childhood Trauma Questionnaire (CTQ), and the Structured Clinical Interview for DSM-IV-TR Axis I Disorders, Research Version, Non-patient Edition (SCID-I/NP). This will determine whether the potential participant is eligible to take part.

If the potential participant meets the requirements based on the outcome of the screening questionnaires and they are willing to participate in the research study, informed consent will be obtained. If the screening questionnaire shows that they do not meet criteria to be in the research project, the investigator will discuss other potential options with them in regards to available research studies.

Once informed consent has been obtained, the participant will take part in research assessments which will occur at baseline, 6, 12 and 24 month time points.

Table 2. *Assessment Schedule*

Week	0	1	2	3	4	5	6	7	8	9	10	11	12	13	14	15	16	17	18	19	20	21	22	23	24	25	26	52	104
Informed consent	X<br>C																												
Medical and Psychiatric history	X<br>C												X														X<br>C	X	X
Concomitant Medication	X												X														X	X	X
Metabolic monitoring	X<br>C*												X														X<br>C*	X<br>C*	C*
Withdrawal criteria		X	X	X	X	X	X	X	X	X	X	X	X	X	X	X	X	X	X	X	X	X	X	X	X	X			
Admission to psychiatric inpatient unit													X														X	X	X
MARS					X				X				X				X				X						X		
UKU Side Effect Rating Scale	X	X	X	X	X								X														X	X	X
CGI	X						X						X														X	X	X

Week	0	1	2	3	4	5	6	7	8	9	10	11	12	13	14	15	16	17	18	19	20	21	22	23	24	25	26	52	104
Demographics	X<br>C												X														X<br>C	X<br>C	X<br>C
SCID-I	X																												
Capsule Dispensing	Completed whenever pills are dispensed																												
Psychological Intervention Checklist	Completed after every OCM appointment																												
Pill Count/ Compliance Rating	Completed whenever pills are returned																												
SCID-II													X																
BPRS (full scale)	X						X						X														X	X	X
SANS	X						X						X														X	X	X
HAM-D	X												X														X	X	X

Week	0	1	2	3	4	5	6	7	8	9	10	11	12	13	14	15	16	17	18	19	20	21	22	23	24	25	26	52	104
HAM-A	X												X														X	X	X
QLS (Heinrichs)	X C																										X C	X C	X C
NOS							X																						
Family History Index (FHI)	X C																												
SOFAS	X C	X	X	X	X	X	X	X	X	X	X	X	X	X	X	X	X	X	X	X	X	X	X	X	X	X	X C	X C	X C
PAS							X																						
WHOQOL-BREF	X C																										X C	X C	X C
ASSIST	X												X														X	X	X
CTQ	X C																												

Week	0	1	2	3	4	5	6	7	8	9	10	11	12	13	14	15	16	17	18	19	20	21	22	23	24	25	26	52	104
GTQ	X																												
Neuropsychology Assessments	X<br>C																										X<br>C	X<br>C	X<br>C
SCID-NP	C																												
Structural imaging	X												X															X	

‘X’: FEP participants; ‘C’: Healthy control participants

* For healthy controls, this will be non-blood measures only (eg. Weight, waist circumference)

2.7.1. Study Withdrawals

FEP participants

Unless consent is withdrawn, all study participants will continue to be monitored and assessed using the study instruments as per the study protocol for the duration of the study regardless of whether they are commenced on anti-psychotic medication due to worsening symptoms or if they decide to withdraw from the medication arm of the trial. If withdrawal criteria are met due to a worsening of mental state, increased risks, or persistent severe psychosis, participants will immediately be offered appropriate treatment, most likely to be the introduction of low-dose anti-psychotic medication in the least restrictive but safe environment. It is possible that, in the event that a participant experiences a worsening of mental state but does not agree to the commencing of anti-psychotic medication, the Mental Health Act may be enforced to allow necessary treatment without the participant’s consent. At every stage of this study the safety of participants will remain the highest priority.

Control participants

Unless consent is withdrawn, all participants will be assessed at baseline, 6, 12 and 24-month time points. If consent is withdrawn, all personal information already collected will be retained and form part of the research project results. If participants do not want their data to be included, they must inform a study personnel member when they withdraw from the study.

2.7.2. Medication withdrawal

There are two clinical scenarios in which the study medication (risperidone or placebo) will need to be withdrawn. The first scenario is when the study period has been completed and the participant has been on the study medication for the six-month period. The second scenario is when the participant meets the withdrawal criteria of the study and needs to be withdrawn from the study medication, e.g. if the participant is over-threshold for risks to self or to others, as determined by the BPRS criteria. As these are two distinct clinical scenarios, they will be considered separately.

Withdrawal due to completion of study period of 6 months

Participants will be administered the study medication for a period of six months and therefore it should be aimed to cease the study medication on the exact date corresponding to end of this six-month period. Depending on the dose of the study medication, this may mean tapering the study medication in the weeks preceding this date.

The decision for maintenance treatment after 6 months should be considered based on a) patient preference b) discussion of the 50% probability that the participant may have been on placebo c) possible side effects d) length of previous psychosis and e) risks related to psychosis.

In a majority of situations where remission is achieved and maintained, it is possible that a decision to remain off antipsychotics will be taken. The study medication should then be tapered at a rate of one tablet per week, corresponding to 1mg of risperidone if the participant is receiving the active medication. During this tapering period, participants should be reviewed at least weekly either by their case manager or doctor and a medical review should occur at least fortnightly during this phase. Participants should continue to be reviewed weekly in the month following the discontinuation of study medication. If a participant experiences a relapse of psychotic symptoms either during or after the discontinuation phase, the decision to commence antipsychotic medication should be based on the frequency, duration and intensity of symptoms. Antipsychotic medication should only be considered if a participant experiences full threshold symptoms for at least seven days and the decision whether to commence antipsychotic medication should be made in consultation with the participant, their caregiver (if appropriate, e.g. under 18 years of age) and the treating team. If it was decided upon to commence an antipsychotic medication, risperidone could be considered as an option. If the participant chooses to continue antipsychotic medication for prophylaxis, then open label Risperidone may be started at 1mg from the day after the last day of blinded risperidone 1mg/placebo.

Withdrawal due to meeting medication withdrawal criteria

During the six-month study period, participants may meet the medication withdrawal criteria and as a result the study medication will need to be discontinued. The study medication should be tapered at a rate of one tablet per week, corresponding to 1mg of risperidone (if the participant is receiving the active medication). However, the tapering could be performed at a faster rate if clinically

indicated (e.g. if the participant is experiencing intolerable side-effects) with the appropriate medical supervision in the appropriate clinical setting.

Meeting the withdrawal criteria does not necessarily mean that an alternative antipsychotic medication should be commenced. However, if clinically indicated, the decision to commence an antipsychotic medication should be made in consultation with the participant, their caregiver (if appropriate, e.g. under 18 years of age) and the treating team. In the circumstances of the study medication being discontinued because the withdrawal criteria has been met, then medications other than risperidone may be preferred as the new antipsychotic medication. The new antipsychotic medication can be commenced as the study medication is being reduced and it should be commenced at a low dose according to the Australian Guidelines for Early Psychosis.

3. Treatment Modalities

3.1. Intensive psychosocial treatment package

Four Components:

1. Case management: A therapist/case manager will be assigned to each study participant. Comprehensive case management including engagement, problem solving, advocacy and support will be provided to all study participants and will provide the context for the provision of the components of the enhanced psychosocial treatment. The EPPIC Case management manual (EPPIC, 2001) provides a detailed description of the content and structure of case management and will be an important resource for study therapists.
2. Close monitoring and crisis response: Participants will be seen at least twice per week by their treating team during the first two months of the study, and will continue to be seen frequently throughout the study. In addition, all study participants will be ‘flagged’ with the crisis response team (YAT) and an individually tailored, written ‘crisis plan’ provided so that rapid assessment and intervention can be provided should this be required during periods when the case manager or psychiatrist are not available. This will ensure that a safety backup plan is in place, and regularly reviewed, for each study participant.
3. CBT/ Cognitively Oriented Psychotherapy for Early Psychosis (COPE): The cognitive behavioural therapy will be a specific therapy developed for the first episode psychosis group and will focus on symptom and stress reduction, facilitating adaptation to illness and treatment

of secondary morbidity including substance misuse. This individually-tailored or formulation-driven psychological therapy will be administered by the case manager/therapist within the case management setting. Therapists will receive regular individual and group supervision and will draw on appropriate therapy resource manuals that have been developed at EPPIC (e.g., COPE, ACE, CAP).

4. Family support and education: As per the standard EPPIC program procedures, therapists/case managers are the first point of contact for families of clients. To maintain consistency in therapy, it is proposed that STAGES case managers continue with this model of contact. They will maintain regular communication with families in order to assist with providing information and education about psychosis and recovery in young people and to provide supportive counselling sessions as required. For complex cases, families will be linked with a family worker for additional support. .

3.2. Medication Protocol

3.2.1. Drugs, formulations and strengths

The double-blind treatment will consist of 1 mg risperidone, and matching placebo tablets. All tablets will be identical in appearance, smell, taste and formulation, except for the active component. Trial medication will be sourced from a GMP provider.

3.2.2. Dosages

The intent is for trial medication administration to be titrated in 1mg increments at the discretion of the treating clinician over a four-week period, up to a maximum maintenance dose of 6 mg/day is reached, if required. Dose will be held at 2mg/day if adequate response is observed within 4 weeks. Medication will be supplied and packaged to allow flexible dosing.

3.2.3. Medication Compliance

Participants will be asked to return medication packaging and compliance will be measured by pill count. In addition, the Medication Adherence Rating Scale (MARS, Thompson, Kulkarni & Sergejew, 2000) will be administered monthly for the duration of the study.

3.2.4. Labelling, storage and accountability

The study medication will be labelled and will comply with local regulatory requirements. The study medication will be stored securely at an appropriate temperature. Accountability records will be maintained. Storage and accountability details will be specified in the STAGES Pharmacy Manual.

3.2.5. Blinding

An independent person will be provided with unblinding envelopes to ensure that unblinding can occur if necessary. Unblinding will only be permitted in the case of a medical emergency when the appropriate management of the patient necessitates knowledge of the treatment randomization. Commencement of antipsychotic treatment due to study withdrawal or worsening symptoms will not be a reason for unblinding. All cases of unblinding will be documented.

3.3. Concomitant therapy

All medications (prescriptions or over-the-counter medications) that are ongoing at the start of the trial or started during the trial will be documented.

The following concomitant medications will be allowed, under the specified circumstances:

Benzodiazepines

Minimal use of benzodiazepines will be allowed during the initial assessment period for the management of insomnia and agitation. Usage will be recorded.

Anti-Depressant Medication

Anti-depressant medication, if clinically indicated, may be continued or commenced during the trial but must be documented for inclusion in analyses.

The following medications are disallowed prior to and during the study:

Lithium and anticonvulsants

If a participant has required lithium or anticonvulsants for the treatment of a prior manic episode he/she will be excluded from the study. If a manic syndrome develops during the course of the study, the participant will be withdrawn and offered an appropriate treatment.

Antipsychotic Medication

During the trial, no concomitant antipsychotic medication will be allowed, and previous treatment with antipsychotic medication is an exclusion criterion for the study. However, brief exposure to antipsychotic medication is permitted without study withdrawal under the following special circumstances:

1. Up to 7 days treatment with antipsychotic medication for the current episode prior to enrolment in the current study will be permitted
2. A lifetime maximum dose of antipsychotic medication equivalent to 1750mg of chlorpromazine.
3. One episode of up to 3 days of antipsychotic medication is permitted for crisis containment, if required, during the course of the study without necessitating withdrawal from the trial.

4. Outcomes and significance

This trial contributes to the recent trend of focusing on functional outcomes, rather than focusing solely on reduction in clinical symptoms (Malla, Norman, & Joober, 2005, Killackey et al., 2006). However, clinical symptoms, remission and recovery will be assessed as secondary outcomes with the BPRS. Remission at 6 months will be defined in accordance with Andreasen et al. (2005) by the relevant BPRS items (grandiosity, suspiciousness, unusual thought content, hallucinatory behavior, conceptual disorganization, mannerism, blunted affect) scored 3 or below.
The study is powered for functioning at 6 month follow up. However, exploratory analysis will also be conducted on 12-, and 24- month functional and clinical outcomes. For the 12- and 24-month follow-up the 6-month time criterion suggested by Andreasen et al. (2005) to define remission will be included in analysis.

The major outcomes of interest in this RCT are whether participants in the placebo group achieve similar levels of functioning and symptomatic improvement compared to the medication group at short- and long-term follow-up. A wide range of factors including DUP, diagnostic features, demographic variables, substance use and social support may be important in determining response to treatment in FEP. However, potential harm will be closely monitored. It is anticipated that a case series of recovery 'successes' will be described and guidelines for the implementation of further RCTs of anti-psychotic-free treatment in FEP including recommendations for inclusion and exclusion criteria will be developed.

In summary, the current trial represents a unique attempt to investigate whether intensive psychosocial treatment and close monitoring in a specialized early intervention service constitutes effective treatment of FEP in a selected subgroup of young people with FEP. It will also shed light on the issue of whether a longer duration of psychotic symptoms before treatment with antipsychotic medication is commenced results in worse symptomatic and functional outcome than a shorter duration. The findings will provide a basis for future larger scale studies and may ultimately have important clinical implications for early intervention in psychotic disorders.

5. Data Management

An appropriate electronic case report form (eCRF) will be used for this study. Data collected in the eCRF will be transmitted via a secure website. Access to the eCRF will be restricted to study personnel and the level of access will be set to maintain the privacy and confidentiality of participant information. A screening log will be maintained. Source documentation (a 'Source File' including information that may be documented in the patient's medical record) that substantiates the information collected in the eCRF will be maintained indefinitely (other study-related documentation as outlined in the Good Clinical Practice guideline will also need to be retained for the same period of time). The eCRF will be managed by staff at Orygen, The National Centre of Excellence in Youth Mental Health, who will also be responsible for data checking and verification.

6. Ethical Considerations

The intensive involvement of case manager and psychiatrist with the participant and the involvement with and support of family members mean that this study can be conducted safely and therefore ethically within the context of EPPIC and Orygen Youth Health. It is our contention that the current research plan meets all of the criteria recommended by Carpenter et al. (2003) and thus can be considered to meet the most recent ethical standards for clinical trials in psychiatry. The proposed RCT addresses these criteria in the following ways:

1) Firstly, community studies suggest that there are a significant number of individuals with psychotic-like experiences who do not decline in functioning or need treatment (Yung et al. 2007; Laurens et al. 2007). It is likely that the intensive psychosocial treatment without medication will

have clinically significant advantages over existing treatments, as the psychosocial intervention is a more benign treatment option which may be suitable for those in an earlier courses of illness (McGorry et al, 2006) and the long-term adverse effects of anti-psychotic medication, including neurologic, endocrine and metabolic effects, have been well documented (see Gardner, Baldessarini, & Waraich, 2005 for a review). The side effects of anti-psychotic medication provide a compelling reason to seek new treatments that are effective and safer than those that are currently available (Carpenter et al, 2003).

2) Entry to the study will be contingent upon informed consent given by the participant and in the case of minors, a parent/legal guardian.

3) Participants in the non-medication arm will not be denied *all* forms of treatment, only antipsychotic medication. These participants will be issued a placebo in order to maintain the blind with those receiving medication, however what is of issue is the effectiveness of an intensive psychosocial treatment delivered to those with FEP.

4) All those referred to EPPIC will be screened in order to assess their eligibility for the study and only those with low risks and adequate family support will be offered randomisation into the trial.

5) With predetermined withdrawal criteria, any participant whose symptoms worsen or can no longer be tolerated, or who experiences a further decline in functioning, will be withdrawn from the trial and offered antipsychotic medication. Furthermore, if a more benign treatment option such as the intensive psychosocial treatment is found to be effective, the benefits for those with FEP will outweigh the risks of being assigned to the non-medication group.

6.1. Regulatory and ethical compliance

This study will not commence until the final protocol, informed consent form and other associated documents are approved by the local institutional ethics committee (IEC). Any protocol amendments will also be managed in accordance with local ethical requirements.

This study will be conducted in accordance with Declaration of Helsinki and Good Clinical Practice guidelines as well as any local regulatory requirements.

7. Governance and Funding

Orygen, The National Centre of Excellence in Youth Mental Health is the sponsor of this study. The study is being funded by an NHMRC program grant awarded to Professor Patrick McGorry. Janssen Research Foundation (JRF) has also provided funding for the study. The following investigators are coordinating the study:

Professor Patrick McGorry
Psychiatrist & Director, Orygen, The National Centre of Excellence in Youth Mental Health & Orygen Youth Health
Locked Bag 10 (35 Poplar Road)
Parkville VIC 3052
Australia
Phone: +61 3 9342 2850
Email: pat.mcgorry@orygen.org.au

Dr Shona Francey
Clinical Psychologist & EPPIC Stream Leader
Orygen, The National Centre of Excellence in Youth Mental Health & Orygen Youth Health
Locked Bag 10 (35 Poplar Road)
Parkville VIC 3052
Australia
Phone: +61 3 9342 2800
Email: shona.francey@orygen.org.au

Dr Barnaby Nelson
Clinical Psychologist & Research Fellow, PACE Clinic
Orygen, The National Centre of Excellence in Youth Mental Health& Orygen Youth Health
Locked Bag 10 (35 Poplar Road)
Parkville VIC 3052
Australia
Phone: +61 3 9342 2800
Email: barnaby.nelson@orygen.org.au

8. References

Allen, J. P., Litten, R. Z., Fertig, J. B., & Babor, T. (1997) A review of research on the Alcohol Use Disorders Identification Test (AUDIT). Alcoholism: Clinical and Experimental Research, 21, 613-619.

Andreasen, N. C. (1983). The Scale for the Assessment of Negative Symptoms (SANS). Iowa City, University of Iowa.

Andreasen N.C., Carpenter WT, Kane JM, Lasser RA, Marder SR, Weinbereger DR (2005) Remission in schizophrenia. Am J Psychiatry 162: 441-49.

Bebbington, P., & Nayani, T. (1995). The Psychosis Screening Questionnaire. *International Journal of Methods in Psychiatric Research, 6,* 11-19.

Bechdolf A, Wagner, M, Veith V, Ruhrmann S, Brockhaus-Dumke A, Pukrop R, Berning J, Stamm E, Janssen B, Decker P, Bottlender R, Maurer KMöller HJ, Gaebel W, Maier W, H Häfner, Klosterkötter J (2007) Preventing progression to first-episode psychosis in early initial prodromal states (submitted).

Birchwood, M. (2000). Early intervention and sustaining the management of vulnerability. *Australian and New Zealand Journal of Psychiatry, 34*, s181-184.

Bola, J. R., & Mosher, L. R. (2003). Treatment of acute psychosis without neuroleptics: Two-year outcomes from the soteria project. *Journal of Nervous and Mental Disease, 191*, 219-229.

Bola, J. R. (2006a). Medication-free research in ealry episode schizophrenia: Evidence of long-term harm? *Schizophrenia Bulletin, 32*, 288-296.

Bola, J. R. (2006b). Psychosocial acute treatment in early-epsiode schizophrenia disorders. *Research on Social Work Practice, 16*, 263-275.

Cannon-Spoor, H. E., S. G. Potkin, et al. (1982). "Measurement of premorbid adjustment in chronic schizophrenia." *Schizophrenia Bulletin 8* (3): 470-484.

Carbone, S., Harrigan, S., McGorry, P. D., Curry, C., & Elkins, K. (1999). Duration of untreated psychosis and 12-month outcome in first-episode psychosis: The impact of treatment approach. *Acta Psychiatrica Scandinavica, 100*, 96-104.

Carpenter, W. T. (1997). The risk of medication-free research. *Schizophrenia Bulletin, 23*, 11-18.

Carpenter, W. T., Appelbaum, P. S., & Levine, R. J. (2003). The declaration of helsinki and clinical trials: A focus on placebo-controlled trials in schizophrenia. *American Journal of Psychiatry, 160*, 356-362.

Coldham, E. L., Addington, J., & Addington, D. (2002). Medication adherence of individuals with a first episode of psychosis. *Acta Psychiatrica Scandinavica, 106*, 286-290.

Conigrave, K. M., Saunders, J. B., & Reznik, R. B. (1995) Predictive capacity of the AUDIT questionnaire for alcohol-related harm. Addiction, 90, 1479-1485.

Corson, P. W., Nopoulos, P., Miller, D. D., Arndt, S., & Andreasen, N. C. (1999). Change in basal ganglia volume over 2 years in patients with schizophrenia: Typical versus atypical neuroleptics. *American Journal of Psychiatry, 156*, 1200-1204.

Creamer M, Burgess P, McFarlane A.C., (2001). Links Post-traumatic stress disorder: findings from the Australian National Survey of Mental Health and Well-being. Psychol Med. 2001 Oct;31(7):1237-47

Crow, T. J., MacMillan, J. F., Johnson, A. L., & Johnstone, E. C. (1986). A randomised controlled trial of prophylactic neuroleptic treatment. *British Journal of Psychiatry, 148*, 120-127.

Cullberg, J., Levander, S., Holmqvist, R., Mattsson, M., & Wieselgren, I. (2002). One-year outcome in first episode psychosis patients in the swedish parachute project. *Acta Psychiatrica Scandinavica, 106*, 276-285.

Daeppen, J. B., Yersin, B., Landry, U., Pecoud, A., & Decrey, H. (2000) Reliability and validity of the Alcohol Use Disorders Identification Test (AUDIT) imbedded within a general health risk screening questionnaire: Results of a survey in 332 primary care patients. Alcoholism: Clinical and Experimental Research, 24, 659-665

de Haan, L., Linszen, D. H., Lenior, M. E., Doderlein de Win, E., & Gorsira, R. (2003). Duration of untreated psychosis and outcome of schizophrenia: Delay in intensive psychosocial treatment versus delay in treatment with antipsychotic medication. *Schizophrenia Bulletin, 29*, 341-348.

Edwards, J., Harris, M. G., & Bapat, S. (2005). Developing services for first-episode psychosis and the critical period. *British Journal of Psychiatry, 187*(suppl. 48), s91-97.

Edwards, J., Elkins, K., Hinton, M., Harrigan, S. M., Donovan, K., Athanasopoulos, O., et al. (2006). Randomized controlled trial of a cannabis-focused intervention for young people with first-episode psychosis. *Acta Psychiatrica Scandinavica, 114*, 109-117.

EPPIC. (2001). *Case management in early psychosis: A handbook*. Melbourne: Early Psychosis Prevention and Intervention Centre.

First, M. B., R. L. Spitzer, et al. (1996). Structured clinical interview for DSM-IV Axis 1 Disorders - Patient Edition (SCID - I/P, Version 2.0). New York, N.Y. State Psychiatric Institute.

First, M. B., R. L. Spitzer, et al. (2002). Structured Clinical Interview for DSM-IV-TR Axis 1 Disorders, Research Version, Non-patient Edition, (SCID - I/NP). New York, N.Y. State

Psychiatric Institute.

Gardner, D. M., Baldessarini, R. J., & Waraich, P. (2005). Modern antipsychotic drugs: A critical review. *Canadian Medical Association Journal, 172*, 1703-1711.

Haddock, G., & Lewis, S. W. (2005). Psychological interventions in early psychosis. *Schizophrenia Bulletin, 31*, 697-704.

Hamilton, M. (1959). "The assessment of anxiety state by rating." *British Journal of Medical Psychology*, *32*, 50-55.

Hamilton, M. (1960). "A rating scale for depression." Journal of Neurology, Neurosurgery and Psychiatry,,: 56-62.

Heinrichs, D. W., T. E. Hanlon, et al. (1984). "The Quality of Life Scale: An instrument for rating the schizophrenic deficit syndrome." *Schizophrenia Bulletin*, *10(3*), 388-96.

Jackson, H. J., McGorry, P. D., Henry, L., Edwards, J., Hulbert, C., Harrigan, S. M., et al. (2001). Cognitively oriented psychotherapy for early psychosis (cope): A 1-year follow-up. *British Journal of Clinical Psychology, 40*, 57-70.

Johns, L. C., & van Os, J. (2001). The continuity of psychotic experiences in the general population. *Clinical Psychology Review, 21*, 1125-1141.

Johnstone, E. C., Owens, D. G. C., Crow, T. J., & Davis, J. M. (1999). Does a four-week delay in the introduction of medication alter the course of functional psychosis? *Journal of Psychopharmacology, 13*, 238-244.

Kalla, O., Aaltonen, J., Wahlstrom, J., Lehtinen, V., Garcia Cabeza, I., & Gonzalez de Chavez, M. (2002). Duration of untreated psychosis and its correlates in first-episode psychosis in finland and spain. *Acta Psychiatrica Scandinavica, 106*, 265-275.

Kane, J. M., & Malhotra, A. (2003). The future of pharmacotherapy for schizophrenia. *World Psychiatry, 2*, 81-86.

Kaul S and Diamond GA. (2006). Good Enough: A primer on the analysis and interpretation of noninferiority trials. *Annals of Internal Medicine, 145*, 62-69.

Laurens, K. R., Hodgins, S., Maughan, B., Murray, R. M., Rutter, M. L., & Taylor, E. A. (2007). Community screening for psychotic-like experiences and other putative antecedents of schizophrenia in children aged 9-12 years. *Schizophrenia Research, 90*, 130-146.

Lehtinen, V., Aaltonen, J., Koffert, T., Rakkolainen, V., & Syvalahti, E. (2000). Two-year outcome in first-episode psychosis treated according to an integrated model. Is immediate neuroleptisation always needed? *European Psychiatry, 15*, 312-320.

Lewis, S. W., Tarrier, N., Haddock, G., Bentall, R. P., Kinderman, P., Kingdon, D., et al. (2002). Randomised controlled trial of cognitive-behavioural therapy in early schizophrenia: Actual-phase outcomes. *British Journal of Psychiatry, 181*(suppl.43), s91-97.

Lieberman JA, Tollefson GD, Charles C, et al.: Antipsychotic drug effects on brain morphology in first-episode psychosis. Arch Gen Psychiatry 2005; 62:361-70

Lieberman, J. A., Tollefson, G. D., Charles, C., Zipursky, R., Sharma, T., Kahn, R. S., et al. (2005). Antipsychotic drug effects on brain morphology in first-episode psychosis. *Archives of General Psychiatry, 62*, 361-370.

Lingjaerde, O., Ahlfors, U. G., Bech, P., Dencker, S. J., & Elgen, K. (1987). The uku side effect rating scale. A new comprehensive rating scale for psychotropic drugs and a cross-sectional study of side effects in neuroleptic-treated patients. *Acta Psychiatrica Scandinavica, 334*, 1-100.

Linszen, D., Lenior, M., De Haan, L., Dingemans, P., & Gersons, B. (1998). Early intervention, untreated psychosis and the course of early schizophrenia. *The British journal of psychiatry. Supplement., 172*(33), 84-89.

Llorca, P. M., Chereau, I., Bayle, F. J., & Lancon, C. (2002). Tardive dyskinesias and antipsychotics: A review. *European Psychiatry, 17*, 129-138.

Loebel, A. D., Lieberman, J. A., Alvir, J. M. J., Mayeroff, D. I., Geisler, S. H., & Szymanski, S. R. (1992). Duration of psychosis and outcome in first-episode schizophrenia. *American Journal of Psychiatry, 149*, 1183-1188.

Malla, A. K., Norman, R. M. G., & Joober, R. (2005). First-episode psychosis, early intervention, and outcome: What have we learned? *Canadian Journal of Psychiatry, 50*, 881-891.

Marshall, M., Lewis, S. W., Lockwood, A., Drake, R., Jones, P., & Croudace, T. (2005). Association between duration of untreated psychosis and outcome in cohorts of first-episode patients. *Archives of General Psychiatry, 62*, 975-983.

McGorry, P. D., Edwards, J., Mihalopoulos, C., Harrigan, S. M., & Jackson, H. J. (1996). Eppic: An evolving system of early detection and optimal management. *Schizophrenia Bulletin, 22*(2), 305-326.

McGorry, P. D., Hickie, I. B., Yung, A. R., Pantelis, C., & Jackson, H. J. (2006). Clinical staging of psychiatric disorders: A heuristic framework for choosing earlier, safer and more effective interventions. *Australian and New Zealand Journal of Psychiatry, 40*, 616-622.

Mosher, L. R. (1999). Soteria and other alternatives to acute psychiatric hospitalization: A personal and professional review. *Journal of Nervous and Mental Disease, 187*, 142-149.

Muench, J., & Carey, M. (2001). Diabetes mellitus associated with atypical antipsychotic medications: New case report and review of the literature. *Journal of the American Board of Family Practice, 14*, 278-282.

Oehl, M., Hummer, M., & Fleischhacker, W. W. (2000). Compliance with antipsychotic treatment. *Acta Psychiatrica Scandinavica, 102*(suppl. 407), 83-86.

Penn, D. L., Waldheter, E. J., Perkins, D. O., Mueser, K. T., & Lieberman, J. A. (2005). Psychosocial treatment for first-episode psychosis: A research update. *American Journal of Psychiatry, 162*, 2220-2232.

Perkins, D. O. (1999). Adherence to antipsychotic medications. *Journal of Clincial Psychiatry, 60*, 25-30.

Perkins, D. O., Johnson, J. L., Hamer, R. M., Zipursky, R. B., Keefe, R. S., Centorrhino, F., et al. (2006). Predictors of antipsychotic medication adherence in patients recovering from a first psychotic episode. *Schizophrenia Research, 83*, 53-63.

Power, P. J. R., Bell, R. J., Mills, R., Herrman-Doig, T., Davern, M., Henry, L., et al. (2003). Suicide prevention in first episode psychosis: The development of a randomised controlled trial of cognitive therapy for acutely suicidal patients with early psychosis. *Australian and New Zealand Journal of Psychiatry, 37*, 414-420.

Remington, G., Kapur, S., & Zipursky, R. B. (1998). Pharmacotherapy of first-episode schizophrenia. *British Journal of Psychiatry, 172*, 66-70.

Saunders, J. B., Aasland, O. G., Babor, T. F., de la Fuente, J. R., & Grant, M. (1993). Development of the alcohol use disorders identification test (audit): Who collaborative project on early detection of persons with harmful alcohol consumption--ii. *Addiction, 88*, 791-804.

Schooler, N. S. (2006). Implications for future research of ‘‘medication-free research in early episode schizophrenia’’. *Schizophrenia Bulletin, 32*, 297–298.

Scott, J., Paykel, E., Morriss, R., Bentall, R., Kinderman, P., Johnson, T., et al., (2006). Cognitive-behavioural therapy for severe and recurrent bipolar disorders: randomised controlled trial. *British Journal of Psychiatry, 188*, 313-320.

Singh, S.P., Cooper, J.E., Fisher, H.L, Tarrant, C.J., Lloyd, T., Banjo, J, Corfe, S., & Jones, P. (2005). Determining the chronology and components of psychosis onset: the Nottingham Onset Schedule (NOS). *Schizophrenia Research, 80,* 117-30.

Snapinn, S.M. (2000). Noninferiority trials. Current Controlled Trials in Cardiovascular Medicine, 1, 19-21.

Szymanski, S. R., Cannon, T. D., Gallacher, F., Erwin, R. J., & Gur, R. E. (1996). Course of treatment response in first-episode and chronic schizophrenia. *American Journal of Psychiatry, 153*, 519-525.

Tarrier, N., Lewis, S. W., Haddock, G., Bentall, R. P., Drake, R., Kinderman, P., et al. (2004). Cognitive-behavioural therapy in first-episode and early schizophrenia: 18-month follow-up of a randomised controlled trial. *British Journal of Psychiatry, 184*, 231-239.

Tarrier, N. (2005). Cognitive behaviour therapy for schizophrenia - a review of development, evidence and implementation. *Psychotherapy and Psychosomatics, 74*, 136-144.

Thompson, K., Kulkarni, J., & Sergejew, A.A. (2000). Reliability and validity of a new Medication Adherence Rating Scale (MARS) for the psychoses. *Schizophrenia Research, 42,* 241-7.

van Os, J., Hanssen, M., Bijl, R. V., & Ravelli, A. (2000). Strauss (1969) revisted: A psychosis continuum in the general population? *Schizophrenia Research, 45*, 11-20.

Ventura, J., Green, M. F., Shaner, A., & Liberman, R. P. (1993). Training and quality assurance with the brief psychiatric rating scale: "the drift busters." *International Journal of Methods in Psychiatric Research, 34*, 221-244.

Ware JH, Antman EM. (1997). Equivalence trials. [Editorial] New England Journal of Medicine, 337, 1159-1161.

WHO ASSIST Working Group (2002). The Alcohol, Smoking and Substance Involvement Screening Test (ASSIST): development, reliability and feasibility. *Addiction, 97 (9):* 1183-1194.

WHOQOL Group. (1998). Development of the World Health Organization WHOQOL-BREF Quality of Life Assessment. *Psychological Medicine*, *28*, 551-558.

Wyatt, R. J., & \Henter, I. (2001). Rationale for the study of early intervention. *Schizophrenia Research, 51*, 69-76.

Yung, A. R., Buckby, J. A., Cotton, S. M., Cosgrave, E. M., Killackey, E. J., Stanford, C., et al. (2006). Psychotic-like experiences in nonpsychotic help-seekers: Associations with distress, depression, and disability. *Schizophrenia Bulletin, 32*, 352-359.

Yung, A. R., Buckby, J. A., Cosgrave, E. M., Killackey, E., Baker, K., Cotton, S. M., et al. (2007). Association between psychotic experiences and depression in a clinical sample over 6 months. *Schizophrenia Research, epub ahead of print*.
