# Supplementary material for: Functional Connectivity in Antipsychotic-Treated and Antipsychotic-Naive Patients With First-Episode Psychosis and Low Risk of Self-harm or Aggression: A Secondary Analysis of a Randomized Clinical Trial
Source: JAMA Psychiatry. 2021 Jun 23;78(9):1–11. doi: 10.1001/jamapsychiatry.2021.1422 (PMC8223142; doi:10.1001/jamapsychiatry.2021.1422)
Supplement: Supplement 2. — MRI Protocol [file jamapsychiatry-e211422-s002.pdf]

The Royal Children's Hospital Melbourne

Medical Imaging Referral

Patient/parent please inform Medical Imaging staff if patient details are incorrect

NAME
ADDRESS
HOSPITAL UR DATE OF BIRTH / /
AFFIX PATIENT LABEL HERE ↑

Print patient's name if label used ____

Examination required (including sedation, general anaesthetic or play therapy)

MRI Brain (Research Protocol)

Patient location ◯ Outpatient ◯ Ward: Room/cubicle number ____

Known allergy ◯ No ◯ Yes, *please specify*

Reason for examination and relevant past history

A randomised placebo controlled trial of intensive psychosocial treatment plus or minus anti-psychotic medication for first episode psychosis with low-risk of self-harm or aggression

All study participants are receiving the intensive psychosocial intervention package that incorporates intensive case management, close monitoring of mental state, cognitive behavioural therapy and family support and education.

Radiologist Report

Standard research MRI report

Consultant

Referring d

Signature

Pager nu

Provider

Imaging Technologist Use

◯ **Positive ID**

Allergy ◯ No ◯ Yes, *please specify* ____

Study label

Sequence	Completed *Yes* *No*	FWHM	T2*	Technical Reasons for Failure
AA Scout	☐ ☐			
T2 Tran FSE	☐ ☐			
MPRAGE	☐ ☐			
Field Map	☐ ☐			
Resting State	☐ ☐			
MGH DTI 60Dir	☐ ☐			
**Follow Up Scan**	☐ ☐			
AA Scout	☐ ☐			
T2 Tran FSE	☐ ☐			
MPRAGE	☐ ☐			
Field Map	☐ ☐			
Resting State	☐ ☐			
Dicom Transfer	☐ ☐			

Comments :

MR Tech		Date :

REVIEWED
By Michael Kean at 12:39 pm, Sep 10, 2012

B17v1.3	Table of contents<br>32 Channel Head Coil	MR062

IMPORTANT - fMRI / DTI Scans aligned 20 degrees to AC-PC Line

\\USER
- Research Projects
  - MNC
    - STAGES (MR062)
      - Baseline Scan
      - AAHScout_32ch
      - T2W FSE
      - MPRAGE - Sagittal
      - 3D GRE SHIM
      - gre_field_mapping
      - Resting State
      - DTI_MGH_60Dir
      - Follow-Up Time Points
      - AAHScout_32ch
      - T2W FSE
      - MPRAGE
      - gre_field_mapping
      - Resting State

Referrer : Dr Alex Fornito
Source : Melbourne Centre for Neurosciences
Source : MNC / OP
Billing : MR062
Interfaces : Nil
Data Export : MNC

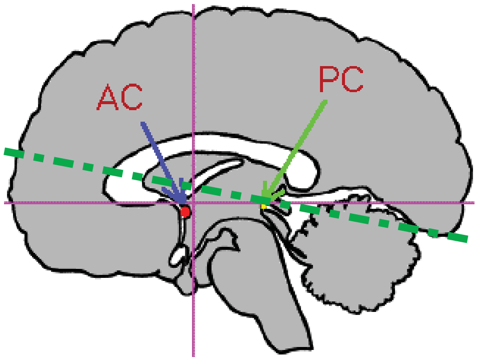

\\USER\Research Projects\MNC\STAGES (MR062)\AAHScout_32ch

TA: 0:28 PAT: Off Voxel size: 1.6×1.6×1.6 mm Rel. SNR: 1.00 SIEMENS: AALScout

Properties

Prio Recon	Off
Before measurement	
After measurement	
Load to viewer	On
Inline movie	Off
Auto store images	On
Load to stamp segments	On
Load images to graphic segments	On
Auto open inline display	Off
Start measurement without further preparation	Off
Wait for user to start	Off
Start measurements	single

Routine

Slab group 1	
Slabs	1
Dist. factor	20 %
Position	L0.0 A11.6 H0.0
Orientation	Sagittal
Phase enc. dir.	A >> P
Rotation	0 deg
AutoAlign	Head
Phase oversampling	0 %
Slice oversampling	0.0 %
Slices per slab	128
FoV read	260 mm
FoV phase	100.0 %
Slice thickness	1.6 mm
TR	3.15 ms
TE	1.37 ms
Averages	1
Concatenations	1
Filter	Prescan Normalize
Coil elements	HEA;HEP

Contrast

Flip angle	8 deg
Averaging mode	Short term
Reconstruction	Magnitude
Measurements	1

Resolution

Base resolution	160
Phase resolution	100 %
Slice resolution	69 %
Phase partial Fourier	6/8
Slice partial Fourier	6/8
PAT mode	None
Matrix Coil Mode	Auto (CP)
Image Filter	Off
Distortion Corr.	Off
Unfiltered images	Off
Prescan Normalize	On
Normalize	Off
B1 filter	Off
Raw filter	Off
Elliptical filter	Off

Geometry

Multi-slice mode	Sequential
Series	Ascending
Set-n-Go Protocol	Off
Table position	H
Table position	0 mm
Inline Composing	Off

System

Body	Off
HEP	On
HEA	On
SP4	Off
SP2	Off
SP8	Off
SP6	Off
SP3	Off
SP1	Off
SP7	Off
SP5	Off
Positioning mode	REF
MSMA	S - C - T
Sagittal	R >> L
Coronal	A >> P
Transversal	F >> H
Save uncombined	Off
Coil Combine Mode	Adaptive Combine
Auto Coil Select	Default
Shim mode	Tune up
Adjust with body coil	Off
Confirm freq. adjustment	Off
Assume Silicone	Off
? Ref. amplitude 1H	0.000 V
Adjustment Tolerance	Auto
Adjust volume	
Position	Isocenter
Orientation	Transversal
Rotation	0.00 deg
R >> L	350 mm
A >> P	263 mm
F >> H	350 mm

Inline

Time to center	11.5 s
MapIt	None
Contrasts	1

Sequence

Introduction	On
Dimension	3D
Asymmetric echo	Weak
Bandwidth	550 Hz/Px
RF pulse type	Fast
Gradient mode	Normal
Excitation	Non-sel.
RF spoiling	On

\\USER\Research Projects\MNC\STAGES (MR062)\T2W FSE

TA: 2:44 PAT: Off Voxel size: 0.8×0.8×4.0 mm Rel. SNR: 1.00 SIEMENS: tse

Properties

Prio Recon	Off
Before measurement	
After measurement	
Load to viewer	On
Inline movie	Off
Auto store images	On
Load to stamp segments	Off
Load images to graphic segments	Off
Auto open inline display	Off
Start measurement without further preparation	On
Wait for user to start	Off
Start measurements	single

Routine

Slice group 1	
Slices	36
Dist. factor	0 %
Position	R0.5 A2.0 F3.4
Orientation	T > C-11.4 > S-2.5
Phase enc. dir.	R >> L
Rotation	90.00 deg
Phase oversampling	20 %
FoV read	240 mm
FoV phase	75.0 %
Slice thickness	4.0 mm
TR	4500 ms
TE	70 ms
Averages	1
Concatenations	2
Filter	Prescan Normalize, Elliptical filter
Coil elements	HEA;HEP

Contrast

TD	0.0 ms
MTC	Off
Magn. preparation	None
Flip angle	136 deg
Fat suppr.	None
Water suppr.	None
Restore magn.	Off
Averaging mode	Long term
Reconstruction	Magnitude
Measurements	1
Multiple series	Each measurement

Resolution

Base resolution	320
Phase resolution	100 %
Phase partial Fourier	Off
Trajectory	Cartesian
Interpolation	Off
PAT mode	None
Matrix Coil Mode	Auto (CP)
Image Filter	Off
Distortion Corr.	Off
Unfiltered images	Off
Prescan Normalize	On
Normalize	Off
B1 filter	Off
Raw filter	Off
Elliptical filter	On
Mode	Inplane

Geometry

Multi-slice mode	Interleaved
Series	Interleaved
Special sat.	Parallel F
Gap	10 mm
Thickness	50 mm
Set-n-Go Protocol	Off
Table position	H
Table position	0 mm
Inline Composing	Off
Tim CT mode	Off

System

Body	Off
HEP	On
HEA	On
SP4	Off
SP2	Off
SP8	Off
SP6	Off
SP3	Off
SP1	Off
SP7	Off
SP5	Off
Positioning mode	REF
MSMA	S - C - T
Sagittal	R >> L
Coronal	A >> P
Transversal	F >> H
Save uncombined	Off
Coil Combine Mode	Adaptive Combine
AutoAlign	---
Auto Coil Select	Default
Shim mode	Standard
Adjust with body coil	Off
Confirm freq. adjustment	Off
Assume Silicone	Off
? Ref. amplitude 1H	0.000 V
Adjustment Tolerance	Auto
Adjust volume	
Position	R0.5 A2.0 F3.4
Orientation	T > C-11.4 > S-2.5
Rotation	90.00 deg
A >> P	240 mm
R >> L	180 mm
F >> H	144 mm

Physio

1st Signal/Mode	None
Dark blood	Off
Resp. control	Off

Inline

Subtract	Off
Std-Dev-Sag	Off
Std-Dev-Cor	Off
Std-Dev-Tra	Off

Std-Dev-Time	Off
MIP-Sag	Off
MIP-Cor	Off
MIP-Tra	Off
MIP-Time	Off
Save original images	On

Sequence

Introduction	On
Dimension	2D
Compensate T2 decay	Off
Reduce Motion Sens.	On
Contrasts	1
Bandwidth	188 Hz/Px
Flow comp.	Slice
Allowed delay	60 s
Echo spacing	11.6 ms
Define	Turbo factor
Turbo factor	17
Echo trains per slice	17
RF pulse type	Fast
Gradient mode	Fast

\\USER\Research Projects\MNC\STAGES (MR062)\MPRAGE

TA: 9:14 PAT: Off Voxel size: 1.0×1.0×1.2 mm Rel. SNR: 1.00 SIEMENS: tfl

Properties

Prio Recon	Off
Before measurement	
After measurement	
Load to viewer	On
Inline movie	Off
Auto store images	On
Load to stamp segments	Off
Load images to graphic segments	Off
Auto open inline display	Off
Start measurement without further preparation	On
Wait for user to start	Off
Start measurements	single

Routine

Slab group 1	
Slabs	1
Dist. factor	50 %
Position	Isocenter
Orientation	Sagittal
Phase enc. dir.	A >> P
Rotation	0.00 deg
Phase oversampling	0 %
Slice oversampling	0.0 %
Slices per slab	176
FoV read	256 mm
FoV phase	93.8 %
Slice thickness	1.20 mm
TR	2300 ms
TE	2.98 ms
Averages	1
Concatenations	1
Filter	Prescan Normalize
Coil elements	HEA;HEP

Contrast

Magn. preparation	Non-sel. IR
TI	900 ms
Flip angle	9 deg
Fat suppr.	None
Water suppr.	None
Averaging mode	Long term
Reconstruction	Magnitude
Measurements	1
Multiple series	Off

Resolution

Base resolution	256
Phase resolution	100 %
Slice resolution	100 %
Phase partial Fourier	Off
Slice partial Fourier	Off
Interpolation	Off
PAT mode	None
Matrix Coil Mode	Auto (CP)
Image Filter	Off
Distortion Corr.	Off
Unfiltered images	Off
Prescan Normalize	On
Normalize	Off
B1 filter	Off
Raw filter	Off
Elliptical filter	Off

Geometry

Multi-slice mode	Single shot
Series	Interleaved
Set-n-Go Protocol	Off
Table position	H
Table position	0 mm
Inline Composing	Off

System

Body	Off
HEP	On
HEA	On
Positioning mode	REF
MSMA	S - C - T
Sagittal	R >> L
Coronal	A >> P
Transversal	F >> H
Save uncombined	Off
Coil Combine Mode	Adaptive Combine
AutoAlign	---
Auto Coil Select	Default
Shim mode	Standard
Adjust with body coil	Off
Confirm freq. adjustment	Off
Assume Silicone	Off
? Ref. amplitude 1H	0.000 V
Adjustment Tolerance	Auto
Adjust volume	
Position	Isocenter
Orientation	Sagittal
Rotation	0.00 deg
F >> H	256 mm
A >> P	240 mm
R >> L	212 mm

Physio

1st Signal/Mode	None
Dark blood	Off
Resp. control	Off

Inline

Subtract	Off
Std-Dev-Sag	Off
Std-Dev-Cor	Off
Std-Dev-Tra	Off
Std-Dev-Time	Off
MIP-Sag	Off
MIP-Cor	Off
MIP-Tra	Off
MIP-Time	Off
Save original images	On

Sequence

Introduction	On
Dimension	3D
Elliptical scanning	Off
Asymmetric echo	Off
Bandwidth	240 Hz/Px
Flow comp.	No

Echo spacing	7.1 ms
RF pulse type	Fast
Gradient mode	Normal
Excitation	Non-sel.
RF spoiling	On

\\USER\Research Projects\MNC\STAGES (MR062)\3D GRE SHIM

TA: 1:04 PAT: Off Voxel size: 2.1×2.1×2.0 mm Rel. SNR: 1.00 USER: CV_shim_452

Properties

Prio Recon	Off
Before measurement	
After measurement	
Load to viewer	Off
Inline movie	Off
Auto store images	On
Load to stamp segments	Off
Load images to graphic segments	Off
Auto open inline display	Off
Start measurement without further preparation	On
Wait for user to start	Off
Start measurements	single

Routine

Slab group 1	
Slabs	1
Dist. factor	20 %
Position	L0.3 A14.9 H19.9
Orientation	T > C-14.7 > S1.8
Phase enc. dir.	R >> L
Rotation	88.00 deg
Auto	Off
Phase oversampling	0 %
Slice oversampling	12.5 %
Slices per slab	64
FoV read	200 mm
FoV phase	75.0 %
Slice thickness	2.00 mm
TR	877.68 ms
TE 1	4.80 ms
TE 2	9.60 ms
Averages	1
Concatenations	1
Filter	Distortion Corr.(2D)
Coil elements	HEA;HEP

Contrast

Magn. preparation	None
Flip angle	12 deg
Fat suppr.	None
Restore magn.	Off
Averaging mode	Short term
Reconstruction	Magnitude
Measurements	1
Multiple series	Off

Resolution

Base resolution	96
Phase resolution	100 %
Slice resolution	100 %
Phase partial Fourier	Off
Slice partial Fourier	Off
Trajectory	Cartesian
Interpolation	Off
PAT mode	None
Matrix Coil Mode	Auto (CP)
Image Filter	Off
Distortion Corr.	On
Mode	2D
Unfiltered images	Off
Prescan Normalize	Off
Normalize	Off
B1 filter	Off
Raw filter	Off
Elliptical filter	Off
POCS	Off

Geometry

Multi-slice mode	Sequential
Series	Ascending
Special sat.	None
Set-n-Go Protocol	Off
Table position	H
Table position	0 mm
Inline Composing	Off

System

Body	Off
HEP	On
HEA	On
SP4	Off
SP2	Off
SP8	Off
SP6	Off
SP3	Off
SP1	Off
SP7	Off
SP5	Off
Positioning mode	FIX
MSMA	S - C - T
Sagittal	R >> L
Coronal	A >> P
Transversal	F >> H
Save uncombined	Off
Coil Combine Mode	Adaptive Combine
AutoAlign	---
Auto Coil Select	Default
Shim mode	Standard
Adjust with body coil	Off
Confirm freq. adjustment	Off
Assume Silicone	Off
? Ref. amplitude 1H	0.000 V
Adjustment Tolerance	Auto
Adjust volume	
! Position	L0.4 A15.6 H16.1
! Orientation	T > C-14.7 > S1.8
! Rotation	-2.00 deg
! R >> L	210 mm
! A >> P	210 mm
! F >> H	130 mm

Physio

1st Signal/Mode	None
Segments	72
Tagging	None
Dark blood	Off
Cine	Off
Resp. control	Off

Inline

Subtract	Off
Std-Dev-Sag	Off

Std-Dev-Cor	Off
Std-Dev-Tra	Off
Std-Dev-Time	Off
MIP-Sag	Off
MIP-Cor	Off
MIP-Tra	Off
MIP-Time	Off
Save original images	On

Sequence

Introduction	Off
Dimension	3D
Elliptical scanning	Off
Reordering	Linear
Asymmetric echo	Off
Contrasts	2
Bandwidth 1	801 Hz/Px
Bandwidth 2	801 Hz/Px
Flow comp. 1	No
Flow comp. 2	No
Readout mode	Monopolar
Optimization	Min. TR
Allowed delay	0 s
Echo spacing	12.2 ms
Sequence type	Gre

Define	Shots
Shots per slice	1
RF pulse type	Normal
Gradient mode	Normal
Excitation	Slab-sel.
Flip angle mode	Constant
RF spoiling	On
Phase Enc. Rewinder	On

\\USER\Research Projects\MNC\STAGES (MR062)\gre_field_mapping

TA: 0:56 Voxel size: 3.3×3.3×3.5 mm Rel. SNR: 1.00 SIEMENS: gre_field_mapping

Properties

Prio Recon	Off
Before measurement	
After measurement	
Load to viewer	On
Inline movie	Off
Auto store images	On
Load to stamp segments	Off
Load images to graphic segments	Off
Auto open inline display	Off
Start measurement without further preparation	On
Wait for user to start	Off
Start measurements	single

Routine

Slice group 1	
Slices	37
Dist. factor	0 %
Position	R3.8 A6.1 H0.0
Orientation	T > C-14.1 > S-2.6
Phase enc. dir.	A >> P
Rotation	0.00 deg
Phase oversampling	0 %
FoV read	210 mm
FoV phase	100.0 %
Slice thickness	3.5 mm
TR	420 ms
TE 1	5.19 ms
TE 2	7.65 ms
Averages	1
Concatenations	1
Filter	None
Coil elements	HEA;HEP

Contrast

MTC	Off
Flip angle	60 deg
Fat suppr.	None
Averaging mode	Long term
Reconstruction	Magn./Phase
Measurements	1
Multiple series	Each measurement

Resolution

Base resolution	64
Phase resolution	100 %
Phase partial Fourier	Off
Interpolation	Off
Matrix Coil Mode	Auto (CP)
Image Filter	Off
Distortion Corr.	Off
Prescan Normalize	Off
Normalize	Off
B1 filter	Off
Raw filter	Off
Elliptical filter	Off

Geometry

Multi-slice mode	Interleaved
Series	Interleaved
Special sat.	None
Set-n-Go Protocol	Off
Table position	H
Table position	0 mm
Inline Composing	Off

System

Body	Off
HEP	On
HEA	On
SP4	Off
SP2	Off
SP8	Off
SP6	Off
SP3	Off
SP1	Off
SP7	Off
SP5	Off
Positioning mode	REF
MSMA	S - C - T
Sagittal	R >> L
Coronal	A >> P
Transversal	F >> H
Save uncombined	Off
Coil Combine Mode	Adaptive Combine
AutoAlign	---
Auto Coil Select	Default
Shim mode	Standard
Adjust with body coil	Off
Confirm freq. adjustment	Off
Assume Silicone	Off
? Ref. amplitude 1H	0.000 V
Adjustment Tolerance	Auto
Adjust volume	
Position	R3.8 A6.1 H0.0
Orientation	T > C-14.1 > S-2.6
Rotation	0.00 deg
R >> L	210 mm
A >> P	210 mm
F >> H	130 mm

Composing

Sequence

Introduction	On
Dimension	2D
Asymmetric echo	Off
Contrasts	2
Bandwidth	260 Hz/Px
Flow comp.	Yes
RF pulse type	Normal
Gradient mode	Normal
RF spoiling	On

\\USER\Research Projects\MNC\STAGES (MR062)\Resting State

TA: 8:00 PAT: 3 Voxel size: 3.3×3.3×3.5 mm Rel. SNR: 1.00 SIEMENS: ep2d_pace

Properties

Prio Recon	Off
Before measurement	
After measurement	
Load to viewer	On
Inline movie	Off
Auto store images	On
Load to stamp segments	Off
Load images to graphic segments	Off
Auto open inline display	Off
Start measurement without further preparation	On
Wait for user to start	On
Start measurements	single

Routine

Slice group 1	
Slices	37
Dist. factor	0 %
Position	R3.8 A6.1 H0.0
Orientation	T > C-14.1 > S-2.6
Phase enc. dir.	A >> P
Rotation	0.00 deg
Phase oversampling	0 %
FoV read	210 mm
FoV phase	100.0 %
Slice thickness	3.5 mm
TR	2000 ms
TE	32.0 ms
Averages	1
Concatenations	1
Filter	Raw filter, Prescan Normalize
Coil elements	HEA;HEP

Contrast

MTC	Off
Flip angle	90 deg
Fat suppr.	Fat sat.
Averaging mode	Long term
Reconstruction	Magnitude
Measurements	234
Delay in TR	0 ms
Multiple series	Off

Resolution

Base resolution	64
Phase resolution	100 %
Phase partial Fourier	Off
Interpolation	Off
PAT mode	GRAPPA
Accel. factor PE	3
Ref. lines PE	36
Matrix Coil Mode	CP
Reference scan mode	Separate
Distortion Corr.	Off
Unfiltered images	Off
Prescan Normalize	On
Raw filter	On
Intensity	Weak
Slope	25
Elliptical filter	Off
Hamming	Off

Geometry

Multi-slice mode	Interleaved
Series	Interleaved
Special sat.	None
Set-n-Go Protocol	Off
Table position	H
Table position	0 mm
Inline Composing	Off

System

Body	Off
HEP	On
HEA	On
SP4	Off
SP2	Off
SP8	Off
SP6	Off
SP3	Off
SP1	Off
SP7	Off
SP5	Off
Positioning mode	FIX
MSMA	S - C - T
Sagittal	R >> L
Coronal	A >> P
Transversal	F >> H
Coil Combine Mode	Sum of Squares
AutoAlign	---
Auto Coil Select	Default
Shim mode	Standard
Adjust with body coil	Off
Confirm freq. adjustment	Off
Assume Silicone	Off
? Ref. amplitude 1H	0.000 V
Adjustment Tolerance	Auto
Adjust volume	
Position	R3.8 A6.1 H0.0
Orientation	T > C-14.1 > S-2.6
Rotation	0.00 deg
R >> L	210 mm
A >> P	210 mm
F >> H	130 mm

Physio

1st Signal/Mode	None

BOLD

GLM Statistics	Off
Dynamic t-maps	Off
Starting ignore meas	2
Ignore after transition	0
Model transition states	Off
Temp. highpass filter	Off
Threshold	4.00
Paradigm size	20
Meas[1]	Baseline
Meas[2]	Baseline
Meas[3]	Baseline
Meas[4]	Active
Meas[5]	Active
Meas[6]	Active
Meas[7]	Active
Meas[8]	Active

Meas[9]	Active
Meas[10]	Active
Meas[11]	Active
Meas[12]	Active
Meas[13]	Active
Meas[14]	Active
Meas[15]	Active
Meas[16]	Active
Meas[17]	Active
Meas[18]	Active
Meas[19]	Active
Meas[20]	Active
Motion correction	Off
Spatial filter	Off

Sequence

Introduction	On
Bandwidth	2520 Hz/Px
Free echo spacing	Off
Echo spacing	0.5 ms
EPI factor	64
RF pulse type	Normal
Gradient mode	Fast*

\\USER\Research Projects\MNC\STAGES (MR062)\DTI_MGH_60Dir

TA: 9:26 PAT: 2 Voxel size: 2.3×2.3×2.3 mm Rel. SNR: 1.00 USER: ep2d_diff_MGH

Properties

Prio Recon	Off
Before measurement	
After measurement	
Load to viewer	On
Inline movie	Off
Auto store images	On
Load to stamp segments	Off
Load images to graphic segments	Off
Auto open inline display	Off
Start measurement without further preparation	On
Wait for user to start	Off
Start measurements	single

Routine

Slice group 1	
Slices	54
Dist. factor	0 %
Position	L0.0 A6.9 F0.7
Orientation	T > C-9.6 > S-2.0
Phase enc. dir.	A >> P
Rotation	0.00 deg
Phase oversampling	0 %
FoV read	240 mm
FoV phase	100.0 %
Slice thickness	2.30 mm
TR	7750 ms
TE	112 ms
Averages	1
Concatenations	1
Filter	Raw filter, Prescan Normalize
Coil elements	HEA;HEP

Contrast

MTC	Off
Magn. preparation	None
Fat suppr.	Fat sat.
Averaging mode	Long term
Reconstruction	Magnitude
Delay in TR	0 ms
Multiple series	Off

Resolution

Base resolution	104
Phase resolution	100 %
Phase partial Fourier	6/8
Interpolation	Off
PAT mode	GRAPPA
Accel. factor PE	2
Ref. lines PE	30
Matrix Coil Mode	Triple
Reference scan mode	Separate
Distortion Corr.	Off
Prescan Normalize	On
Raw filter	On
Intensity	Weak
Slope	25
Elliptical filter	Off
Hamming	Off

Geometry

Multi-slice mode	Interleaved
Series	Interleaved
Special sat.	None
Set-n-Go Protocol	Off
Table position	H
Table position	0 mm
Inline Composing	Off

System

Body	Off
HEP	On
HEA	On
SP4	Off
SP2	Off
SP8	Off
SP6	Off
SP3	Off
SP1	Off
SP7	Off
SP5	Off
Positioning mode	FIX
MSMA	S - C - T
Sagittal	R >> L
Coronal	A >> P
Transversal	F >> H
Coil Combine Mode	Adaptive Combine
AutoAlign	---
Auto Coil Select	Default
Shim mode	Standard
Adjust with body coil	Off
Confirm freq. adjustment	Off
Assume Silicone	Off
? Ref. amplitude 1H	0.000 V
Adjustment Tolerance	Auto
Adjust volume	
Position	L0.0 A6.9 F0.7
Orientation	T > C-9.6 > S-2.0
Rotation	0.00 deg
R >> L	240 mm
A >> P	240 mm
F >> H	125 mm

Physio

1st Signal/Mode	None
Resp. control	Off

Diff

Diffusion mode	MDDW
Diff. weightings	2
b-value 1	0 s/mm²
b-value 2	3000 s/mm²
Mosaic	On
Noise level	40
Diff. directions	60

Sequence

Introduction	Off
Bandwidth	2290 Hz/Px
Free echo spacing	Off
Echo spacing	0.57 ms
EPI factor	104
RF pulse type	Normal

Gradient mode	Fast
Sequence Mode	Product
Diff Grad Table	Single
Direction Scheme	Single
Dummy Scans	3
T2 Weighted Images	10
FFT Scale Factor	0.00

\\USER\Research Projects\MNC\STAGES (MR062)\AAHScout_32ch

TA: 0:28 PAT: Off Voxel size: 1.6×1.6×1.6 mm Rel. SNR: 1.00 SIEMENS: AALScout

Properties

Prio Recon	Off
Before measurement	
After measurement	
Load to viewer	On
Inline movie	Off
Auto store images	On
Load to stamp segments	On
Load images to graphic segments	On
Auto open inline display	Off
Start measurement without further preparation	Off
Wait for user to start	Off
Start measurements	single

Routine

Slab group 1	
Slabs	1
Dist. factor	20 %
Position	L0.0 A11.6 H0.0
Orientation	Sagittal
Phase enc. dir.	A >> P
Rotation	0 deg
AutoAlign	Head
Phase oversampling	0 %
Slice oversampling	0.0 %
Slices per slab	128
FoV read	260 mm
FoV phase	100.0 %
Slice thickness	1.6 mm
TR	3.15 ms
TE	1.37 ms
Averages	1
Concatenations	1
Filter	Prescan Normalize
Coil elements	HEA;HEP

Contrast

Flip angle	8 deg
Averaging mode	Short term
Reconstruction	Magnitude
Measurements	1

Resolution

Base resolution	160
Phase resolution	100 %
Slice resolution	69 %
Phase partial Fourier	6/8
Slice partial Fourier	6/8
PAT mode	None
Matrix Coil Mode	Auto (CP)
Image Filter	Off
Distortion Corr.	Off
Unfiltered images	Off
Prescan Normalize	On
Normalize	Off
B1 filter	Off
Raw filter	Off
Elliptical filter	Off

Geometry

Multi-slice mode	Sequential
Series	Ascending
Set-n-Go Protocol	Off
Table position	H
Table position	0 mm
Inline Composing	Off

System

Body	Off
HEP	On
HEA	On
SP4	Off
SP2	Off
SP8	Off
SP6	Off
SP3	Off
SP1	Off
SP7	Off
SP5	Off
Positioning mode	REF
MSMA	S - C - T
Sagittal	R >> L
Coronal	A >> P
Transversal	F >> H
Save uncombined	Off
Coil Combine Mode	Adaptive Combine
Auto Coil Select	Default
Shim mode	Tune up
Adjust with body coil	Off
Confirm freq. adjustment	Off
Assume Silicone	Off
? Ref. amplitude 1H	0.000 V
Adjustment Tolerance	Auto
Adjust volume	
Position	Isocenter
Orientation	Transversal
Rotation	0.00 deg
R >> L	350 mm
A >> P	263 mm
F >> H	350 mm

Inline

Time to center	11.5 s
MapIt	None
Contrasts	1

Sequence

Introduction	On
Dimension	3D
Asymmetric echo	Weak
Bandwidth	550 Hz/Px
RF pulse type	Fast
Gradient mode	Normal
Excitation	Non-sel.
RF spoiling	On

\\USER\Research Projects\MNC\STAGES (MR062)\T2W FSE

TA: 2:44 PAT: Off Voxel size: 0.8×0.8×4.0 mm Rel. SNR: 1.00 SIEMENS: tse

Properties

Prio Recon	Off
Before measurement	
After measurement	
Load to viewer	On
Inline movie	Off
Auto store images	On
Load to stamp segments	Off
Load images to graphic segments	Off
Auto open inline display	Off
Start measurement without further preparation	On
Wait for user to start	Off
Start measurements	single

Routine

Slice group 1	
Slices	36
Dist. factor	0 %
Position	R0.5 A2.0 F3.4
Orientation	T > C-14.0 > S-1.8
Phase enc. dir.	R >> L
Rotation	90.00 deg
Phase oversampling	20 %
FoV read	240 mm
FoV phase	75.0 %
Slice thickness	4.0 mm
TR	4500 ms
TE	70 ms
Averages	1
Concatenations	2
Filter	Prescan Normalize, Elliptical filter
Coil elements	HEA;HEP

Contrast

TD	0.0 ms
MTC	Off
Magn. preparation	None
Flip angle	136 deg
Fat suppr.	None
Water suppr.	None
Restore magn.	Off
Averaging mode	Long term
Reconstruction	Magnitude
Measurements	1
Multiple series	Each measurement

Resolution

Base resolution	320
Phase resolution	100 %
Phase partial Fourier	Off
Trajectory	Cartesian
Interpolation	Off
PAT mode	None
Matrix Coil Mode	Auto (CP)
Image Filter	Off
Distortion Corr.	Off
Unfiltered images	Off
Prescan Normalize	On
Normalize	Off
B1 filter	Off
Raw filter	Off
Elliptical filter	On
Mode	Inplane

Geometry

Multi-slice mode	Interleaved
Series	Interleaved
Special sat.	Parallel F
Gap	10 mm
Thickness	50 mm
Set-n-Go Protocol	Off
Table position	H
Table position	0 mm
Inline Composing	Off
Tim CT mode	Off

System

Body	Off
HEP	On
HEA	On
SP4	Off
SP2	Off
SP8	Off
SP6	Off
SP3	Off
SP1	Off
SP7	Off
SP5	Off
Positioning mode	REF
MSMA	S - C - T
Sagittal	R >> L
Coronal	A >> P
Transversal	F >> H
Save uncombined	Off
Coil Combine Mode	Adaptive Combine
AutoAlign	---
Auto Coil Select	Default
Shim mode	Standard
Adjust with body coil	Off
Confirm freq. adjustment	Off
Assume Silicone	Off
? Ref. amplitude 1H	0.000 V
Adjustment Tolerance	Auto
Adjust volume	
Position	R0.5 A2.0 F3.4
Orientation	T > C-14.0 > S-1.8
Rotation	90.00 deg
A >> P	240 mm
R >> L	180 mm
F >> H	144 mm

Physio

1st Signal/Mode	None
Dark blood	Off
Resp. control	Off

Inline

Subtract	Off
Std-Dev-Sag	Off
Std-Dev-Cor	Off
Std-Dev-Tra	Off

Std-Dev-Time	Off
MIP-Sag	Off
MIP-Cor	Off
MIP-Tra	Off
MIP-Time	Off
Save original images	On

Sequence

Introduction	On
Dimension	2D
Compensate T2 decay	Off
Reduce Motion Sens.	On
Contrasts	1
Bandwidth	188 Hz/Px
Flow comp.	Slice
Allowed delay	60 s
Echo spacing	11.6 ms
Define	Turbo factor
Turbo factor	17
Echo trains per slice	17
RF pulse type	Fast
Gradient mode	Fast

\\USER\Research Projects\MNC\STAGES (MR062)\MPRAGE

TA: 9:14 PAT: Off Voxel size: 1.0×1.0×1.2 mm Rel. SNR: 1.00 SIEMENS: tfl

Properties

Prio Recon	Off
Before measurement	
After measurement	
Load to viewer	On
Inline movie	Off
Auto store images	On
Load to stamp segments	Off
Load images to graphic segments	Off
Auto open inline display	Off
Start measurement without further preparation	On
Wait for user to start	Off
Start measurements	single

Routine

Slab group 1	
Slabs	1
Dist. factor	50 %
Position	Isocenter
Orientation	Sagittal
Phase enc. dir.	A >> P
Rotation	0.00 deg
Phase oversampling	0 %
Slice oversampling	0.0 %
Slices per slab	176
FoV read	256 mm
FoV phase	93.8 %
Slice thickness	1.20 mm
TR	2300 ms
TE	2.98 ms
Averages	1
Concatenations	1
Filter	Prescan Normalize
Coil elements	HEA;HEP

Contrast

Magn. preparation	Non-sel. IR
TI	900 ms
Flip angle	9 deg
Fat suppr.	None
Water suppr.	None
Averaging mode	Long term
Reconstruction	Magnitude
Measurements	1
Multiple series	Off

Resolution

Base resolution	256
Phase resolution	100 %
Slice resolution	100 %
Phase partial Fourier	Off
Slice partial Fourier	Off
Interpolation	Off
PAT mode	None
Matrix Coil Mode	Auto (CP)
Image Filter	Off
Distortion Corr.	Off
Unfiltered images	Off
Prescan Normalize	On
Normalize	Off
B1 filter	Off
Raw filter	Off
Elliptical filter	Off

Geometry

Multi-slice mode	Single shot
Series	Interleaved
Set-n-Go Protocol	Off
Table position	H
Table position	0 mm
Inline Composing	Off

System

Body	Off
HEP	On
HEA	On
Positioning mode	REF
MSMA	S - C - T
Sagittal	R >> L
Coronal	A >> P
Transversal	F >> H
Save uncombined	Off
Coil Combine Mode	Adaptive Combine
AutoAlign	---
Auto Coil Select	Default
Shim mode	Standard
Adjust with body coil	Off
Confirm freq. adjustment	Off
Assume Silicone	Off
? Ref. amplitude 1H	0.000 V
Adjustment Tolerance	Auto
Adjust volume	
Position	Isocenter
Orientation	Sagittal
Rotation	0.00 deg
F >> H	256 mm
A >> P	240 mm
R >> L	212 mm

Physio

1st Signal/Mode	None
Dark blood	Off
Resp. control	Off

Inline

Subtract	Off
Std-Dev-Sag	Off
Std-Dev-Cor	Off
Std-Dev-Tra	Off
Std-Dev-Time	Off
MIP-Sag	Off
MIP-Cor	Off
MIP-Tra	Off
MIP-Time	Off
Save original images	On

Sequence

Introduction	On
Dimension	3D
Elliptical scanning	Off
Asymmetric echo	Off
Bandwidth	240 Hz/Px
Flow comp.	No

Echo spacing	7.1 ms
RF pulse type	Fast
Gradient mode	Normal
Excitation	Non-sel.
RF spoiling	On

\\USER\Research Projects\MNC\STAGES (MR062)\gre_field_mapping

TA: 0:56 Voxel size: 3.3×3.3×3.5 mm Rel. SNR: 1.00 SIEMENS: gre_field_mapping

Properties

Prio Recon	Off
Before measurement	
After measurement	
Load to viewer	On
Inline movie	Off
Auto store images	On
Load to stamp segments	Off
Load images to graphic segments	Off
Auto open inline display	Off
Start measurement without further preparation	On
Wait for user to start	Off
Start measurements	single

Routine

Slice group 1	
Slices	37
Dist. factor	0 %
Position	R3.6 A11.6 H1.6
Orientation	T > C-11.1 > S-2.0
Phase enc. dir.	A >> P
Rotation	0.00 deg
Phase oversampling	0 %
FoV read	210 mm
FoV phase	100.0 %
Slice thickness	3.5 mm
TR	420 ms
TE 1	5.19 ms
TE 2	7.65 ms
Averages	1
Concatenations	1
Filter	None
Coil elements	HEA;HEP

Contrast

MTC	Off
Flip angle	60 deg
Fat suppr.	None
Averaging mode	Long term
Reconstruction	Magn./Phase
Measurements	1
Multiple series	Each measurement

Resolution

Base resolution	64
Phase resolution	100 %
Phase partial Fourier	Off
Interpolation	Off
Matrix Coil Mode	Auto (CP)
Image Filter	Off
Distortion Corr.	Off
Prescan Normalize	Off
Normalize	Off
B1 filter	Off
Raw filter	Off
Elliptical filter	Off

Geometry

Multi-slice mode	Interleaved
Series	Interleaved
Special sat.	None
Set-n-Go Protocol	Off
Table position	H
Table position	0 mm
Inline Composing	Off

System

Body	Off
HEP	On
HEA	On
SP4	Off
SP2	Off
SP8	Off
SP6	Off
SP3	Off
SP1	Off
SP7	Off
SP5	Off
Positioning mode	REF
MSMA	S - C - T
Sagittal	R >> L
Coronal	A >> P
Transversal	F >> H
Save uncombined	Off
Coil Combine Mode	Adaptive Combine
AutoAlign	---
Auto Coil Select	Default
Shim mode	Standard
Adjust with body coil	Off
Confirm freq. adjustment	Off
Assume Silicone	Off
? Ref. amplitude 1H	0.000 V
Adjustment Tolerance	Auto
Adjust volume	
Position	R3.6 A11.6 H1.6
Orientation	T > C-11.1 > S-2.0
Rotation	0.00 deg
R >> L	210 mm
A >> P	210 mm
F >> H	130 mm

Composing

Sequence

Introduction	On
Dimension	2D
Asymmetric echo	Off
Contrasts	2
Bandwidth	260 Hz/Px
Flow comp.	Yes
RF pulse type	Normal
Gradient mode	Normal
RF spoiling	On

\\USER\Research Projects\MNC\STAGES (MR062)\Resting State

TA: 8:00 PAT: 3 Voxel size: 3.3×3.3×3.5 mm Rel. SNR: 1.00 SIEMENS: ep2d_pace

Properties

Prio Recon	Off
Before measurement	
After measurement	
Load to viewer	On
Inline movie	Off
Auto store images	On
Load to stamp segments	Off
Load images to graphic segments	Off
Auto open inline display	Off
Start measurement without further preparation	On
Wait for user to start	On
Start measurements	single

Routine

Slice group 1	
Slices	37
Dist. factor	0 %
Position	R3.6 A11.6 H1.6
Orientation	T > C-11.1 > S-2.0
Phase enc. dir.	A >> P
Rotation	0.00 deg
Phase oversampling	0 %
FoV read	210 mm
FoV phase	100.0 %
Slice thickness	3.5 mm
TR	2000 ms
TE	32.0 ms
Averages	1
Concatenations	1
Filter	Raw filter, Prescan Normalize
Coil elements	HEA;HEP

Contrast

MTC	Off
Flip angle	90 deg
Fat suppr.	Fat sat.
Averaging mode	Long term
Reconstruction	Magnitude
Measurements	234
Delay in TR	0 ms
Multiple series	Off

Resolution

Base resolution	64
Phase resolution	100 %
Phase partial Fourier	Off
Interpolation	Off
PAT mode	GRAPPA
Accel. factor PE	3
Ref. lines PE	36
Matrix Coil Mode	CP
Reference scan mode	Separate
Distortion Corr.	Off
Unfiltered images	Off
Prescan Normalize	On
Raw filter	On
Intensity	Weak
Slope	25
Elliptical filter	Off
Hamming	Off

Geometry

Multi-slice mode	Interleaved
Series	Interleaved
Special sat.	None
Set-n-Go Protocol	Off
Table position	H
Table position	0 mm
Inline Composing	Off

System

Body	Off
HEP	On
HEA	On
SP4	Off
SP2	Off
SP8	Off
SP6	Off
SP3	Off
SP1	Off
SP7	Off
SP5	Off
Positioning mode	FIX
MSMA	S - C - T
Sagittal	R >> L
Coronal	A >> P
Transversal	F >> H
Coil Combine Mode	Sum of Squares
AutoAlign	---
Auto Coil Select	Default
Shim mode	Standard
Adjust with body coil	Off
Confirm freq. adjustment	Off
Assume Silicone	Off
? Ref. amplitude 1H	0.000 V
Adjustment Tolerance	Auto
Adjust volume	
Position	R3.6 A11.6 H1.6
Orientation	T > C-11.1 > S-2.0
Rotation	0.00 deg
R >> L	210 mm
A >> P	210 mm
F >> H	130 mm

Physio

1st Signal/Mode	None

BOLD

GLM Statistics	Off
Dynamic t-maps	Off
Starting ignore meas	2
Ignore after transition	0
Model transition states	Off
Temp. highpass filter	Off
Threshold	4.00
Paradigm size	20
Meas[1]	Baseline
Meas[2]	Baseline
Meas[3]	Baseline
Meas[4]	Active
Meas[5]	Active
Meas[6]	Active
Meas[7]	Active
Meas[8]	Active

Meas[9]	Active
Meas[10]	Active
Meas[11]	Active
Meas[12]	Active
Meas[13]	Active
Meas[14]	Active
Meas[15]	Active
Meas[16]	Active
Meas[17]	Active
Meas[18]	Active
Meas[19]	Active
Meas[20]	Active
Motion correction	Off
Spatial filter	Off

Sequence

Introduction	On
Bandwidth	2520 Hz/Px
Free echo spacing	Off
Echo spacing	0.5 ms
EPI factor	64
RF pulse type	Normal
Gradient mode	Fast*
