# Supplementary material for: Functional Connectivity in Antipsychotic-Treated and Antipsychotic-Naive Patients With First-Episode Psychosis and Low Risk of Self-harm or Aggression: A Secondary Analysis of a Randomized Clinical Trial
Source: JAMA Psychiatry. 2021 Jun 23;78(9):1–11. doi: 10.1001/jamapsychiatry.2021.1422 (PMC8223142; doi:10.1001/jamapsychiatry.2021.1422)
Supplement: Supplement 3. — eMethods 1. Additional Details eMethods 2. Trial Safety Procedures eMethods 3. Antipsychotic and Concomitant Medication Details eTable 1. Cumulative Antipsychotic Exposure (olanzapine equivalents, mg) in Placebo Group (PIPT) and Medication Group (MIPT) eTable 2. Percentage of Each Treatment Group Included in Analyses Who Received Nonantipsychotic Psychotropic Medication During the Primary Treatment Period eFigure 1. Histogram of Cumulative Dose (olanzapine equivalents, mg) Exposure in the Placebo and Medication Groups Across the Three Time Points eMethods 4. MRI Acquisition Details eMethods 5. Image Processing and Quality Control eMethods 6. Further Details on Statistical Analysis eMethods 7. Demographic and Sample Characteristics eAppendix 1. Antipsychotic-Naïve Results (Baseline) eAppendix 2. Antipsychotic-Naïve Results (Baseline to 3 mo) eAppendix 3. Antipsychotic-Related Results (Baseline to 3 mo) eAppendix 4. Long-Term Changes in Antipsychotic-Naïve Patients Compared to Healthy Controls (Baseline to 12 mo) eAppendix 5. Long-Term Antipsychotic-Related Changes (Baseline to 12 mo) eAppendix 6. Association Between Long-Term Changes and Symptoms and Functioning eFigure 2. Canonical Correlation Analysis Between Short-Term Functional Connectivity Changes and Behavioral Outcomes eFigure 3. Longer-Term Effects in Antipsychotic-Naïve Patients (A, B) and Longer-Term Effects Related to Antipsychotic Medication (C,D) eFigure 4. Antipsychotic-Naïve and Antipsychotic-Treated Results at a Network Level (FWE P < .05) at Primary Thresholds of P < .01 and P < .001 eFigure 5. Baseline to 12 Months Antipsychotic-Naïve and Antipsychotic-Related Effects After Removing the 9 Subjects in the PIPT Group Who Were Exposed to Antipsychotics eFigure 6. Baseline to 3 Months Antipsychotic-Naïve and Antipsychotic-Related Effects After Adjusting for Nonantipsychotic Psychotropic Medication Use eReferences [file jamapsychiatry-e211422-s003.pdf]

Supplementary Online Content

Chopra S, Francey SM, O'Donoghue B, et al. Changes in functional connectivity in antipsychotic-treated and antipsychotic naive patients: a secondary analysis of a randomized clinical trial. *JAMA Psychiatry*. Published online June 23, 2021. doi:10.1001/jamapsychiatry.2021.1422

eMethods 1. Additional Details

eMethods 2. Trial Safety Procedures

eMethods 3. Antipsychotic and Concomitant Medication Details

eTable 1. Cumulative Antipsychotic Exposure (olanzapine equivalents, mg) in Placebo Group (PIPT) and Medication Group (MIPT)

eTable 2. Percentage of Each Treatment Group Included in Analyses Who Received Nonantipsychotic Psychotropic Medication During the Primary Treatment Period

eFigure 1. Histogram of Cumulative Dose (olanzapine equivalents, mg) Exposure in the Placebo and Medication Groups Across the Three Time Points

eMethods 4. MRI Acquisition Details

eMethods 5. Image Processing and Quality Control

eMethods 6. Further Details on Statistical Analysis

eMethods 7. Demographic and Sample Characteristics

eAppendix 1. Antipsychotic-Naïve Results (Baseline)

eAppendix 2. Antipsychotic-Naïve Results (Baseline to 3 mo)

eAppendix 3. Antipsychotic-Related Results (Baseline to 3 mo)

eAppendix 4. Long-Term Changes in Antipsychotic-Naïve Patients Compared to Healthy Controls (Baseline to 12 mo)

eAppendix 5. Long-Term Antipsychotic-Related Changes (Baseline to 12 mo)

eAppendix 6. Association Between Long-Term Changes and Symptoms and Functioning

eFigure 2. Canonical Correlation Analysis Between Short-Term Functional Connectivity Changes and Behavioral Outcomes

© 2021 Chopra S et al. *JAMA Psychiatry.*

eFigure 3. Longer-Term Effects in Antipsychotic-Naïve Patients (A, B) and Longer-Term Effects Related to Antipsychotic Medication (C,D)

eFigure 4. Antipsychotic-Naïve and Antipsychotic-Treated Results at a Network Level (FWE $P < .05$) at Primary Thresholds of $P < .01$ and $P < .001$

eFigure 5. Baseline to 12 Months Antipsychotic-Naïve and Antipsychotic-Related Effects After Removing the 9 Subjects in the PIPT Group Who Were Exposed to Antipsychotics

eFigure 6. Baseline to 3 Months Antipsychotic-Naïve and Antipsychotic-Related Effects After Adjusting for Nonantipsychotic Psychotropic Medication Use

eReferences

This supplementary material has been provided by the authors to give readers additional information about their work.

© 2021 Chopra S et al. *JAMA Psychiatry*.

eMethods 1. Additional Details

The trial took place at the Early Psychosis Prevention and Intervention Centre, which is part of Orygen Youth Health, Melbourne, Australia. The randomization phase of the study terminated at 6 months, so patients in either the MIPT or PIPT group could have received antipsychotic medication and ongoing psychosocial interventions in between 6 and 12 months into the study.

eMethods 2. Trial Safety Procedures

To further ensure safety, several discontinuation criteria were applied in the clinical trial. These were operationally defined as any of the following[1,2]: increased risk to self or others (score of ≥5 on the BPRS-4 Suicidality or Hostility subscales, maintained for 1 week); increase in positive psychotic symptom severity (2-point increase on the BPRS-4 subscale of Conceptual Disorganisation, Hallucinations, Unusual Thought Content, or Suspiciousness) maintained for at least 1 week not due to substance use; decrease in overall functioning (20-point drop in SOFAS score from baseline maintained for 1 month); request by the participant for the introduction of antipsychotic medication; failure to satisfactorily recover 3 months after study entry (a score of 5 or more on the BPRS-4 Hallucinations, Suspiciousness, and Unusual Thought Content subscales or a score of 4 or greater on Conceptual Disorganisation); or becoming pregnant. All participants gave written informed consent after having the study fully explained to them, parental consent was also obtained for participants under the age of 18.

eMethods 3. Antipsychotic and Concomitant Medication Details

The current study took place in the context of a larger clinical trial examining functional and clinical outcome[1], in which the analyses included patients in the PIPT group who switched to antipsychotics. In the current study, we only included patients in the PIPT group who remained on placebo to more clearly delineate the effects of antipsychotic exposure.

Four patients within the PIPT group were switched to open-label antipsychotic medication before the 3-month MRI scan and were excluded from the primary analysis. Two additional individuals were exposed to antipsychotics at doses lower than the minimal exposure limit for inclusion in our study. Excluding these two additional participants from the analysis yielded a qualitatively similar pattern of results. After termination of the randomization phase at 6 months, five additional patients in the PIPT group were exposed to antipsychotic medication

© 2021 Chopra S et al. *JAMA Psychiatry.*

between the 3-month and 12-month scans. Mean cumulative dose and rates of exposure for both patient groups at each timepoint are provided in Supplementary Table 1. Concomitant medications were permitted during the trial, except for additional antipsychotics or mood stabilisers. Rates of concomitant medication use between the two treatment groups were not significantly different (Supplementary Table 2). To ensure that non-antipsychotic psychotropics did not have a large impact on our primary findings, we recomputed our analyses, this time including a binary nuisance covariate for each of the three medication classes. The findings remain largely the same (Supplementary Figure 5).

eTable 1. Cumulative Antipsychotic Exposure (olanzapine equivalents, mg) in Placebo Group (PIPT) and Medication Group (MIPT)

	Baseline	3-months	12-months
PIPT, mg (min; max; median; n[1])	0; 3; 0; 0	0; 959; 0; 4[2]	0; 4177; 0.5; 9
MIPT, mg (min; max; median)	0; 30; 0	168; 993; 369	330; 3229; 812

[1]Number of patients in the placebo group who were exposed to antipsychotic medication at amounts greater than mandated by the minimal study inclusion criterion (lifetime 58mg olanzapine equivalents exposure).

[2] Note: these four patients were excluded from the primary analysis; thus, cumulative antipsychotic exposure of the analysis sample was min= 0; max = 55; median = 0.

eTable 2. Percentage of Each Treatment Group Included in Analyses Who Received Nonantipsychotic Psychotropic Medication During the Primary Treatment Period

	Benzodiazepine	Antidepressant	Other[1]
PIPT, %	30.0	56.7	30.0
MIPT, %	62.0	51.7	41.4

[1] This category included people taking zopiclone, dexamethasone, benztropine and clonidine.

There were no significant differences between rates of non-antipsychotic psychotropic medication use during the primary treatment period in benzodiazepine ($\chi^2 = 2.94; p = 0.086$), antidepressant ($\chi^2 = 0.118; p = 0.732$), or other ($\chi^2 = 0.567; p = 0.452$).

© 2021 Chopra S et al. *JAMA Psychiatry.*

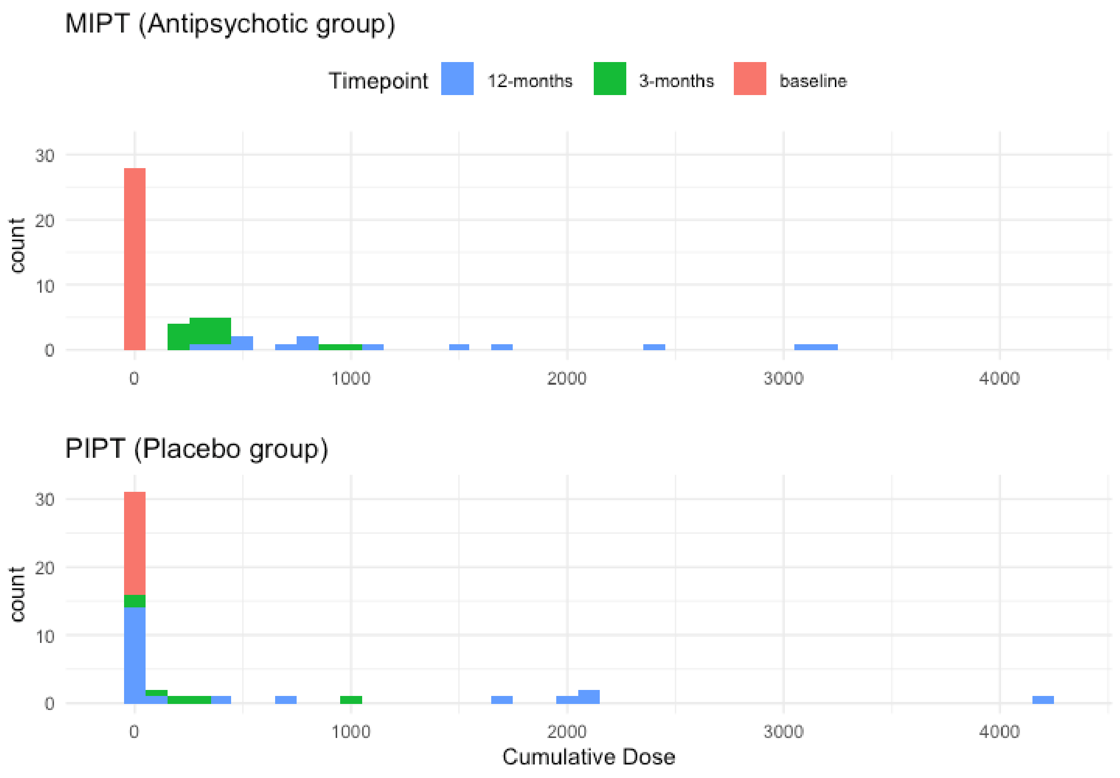

eFigure 1. Histogram of Cumulative Dose (olanzapine equivalents, mg) Exposure in the Placebo and Medication Groups Across the Three Time Points

eMethods 4. MRI Acquisition Details

The 3T Siemens Trio Tim scanner equipped with a 32-channel head coil was located at the Royal Children's Hospital in Melbourne, Australia. Participants were instructed to lie still in the scanner while keeping they eyes closed and maintaining wakefulness. A total of 234 functional volumes with 37 slices each and an interleaved acquisition, were acquired using the following parameters: repetition time =

2000ms; echo time = 32ms; flip angle = 90°; field of view = 210mm; slice thickness of 3.5 mm, and 3.3 x 3.3 x 3.55 mm voxels. For each participant's T1-weighted image, a total of 176 slices were acquired with an interleaved acquisition using the following parameters: TR = 2300ms; TE = 2.98ms; flip angle of 9°; FOV of 256mm; voxel size of 1.1 x 1.1. x 1.2 mm.

© 2021 Chopra S et al. *JAMA Psychiatry*.

eMethods 5. Image Processing and Quality Control

A total of 202 rs-fMRI datasets were acquired in this study. Raw images were first put through an automated quality control procedure (MRI-QC)[3], which resulted in the exclusion of 4 scans due to large artefacts. All remaining images were then processed using a standardised pipeline (*fmriprep*[4]). Briefly, the pipeline included slice time correction, non-linear spatial normalisation to MNI space, brain tissue segmentation, susceptibility distortion estimation and resampling to 2mm. We used previously suggested stringent criteria for excluding and an additional 5 datasets on the basis of excess motion[5]. Automated ICA-based artefact removal (ICA-AROMA)[6] was applied, then averaged signals from the white matter, CSF, and entire brain were removed from voxelwise time series via linear regression, prior to detrending and high-pass filtering ($f > .005$ Hz). At each stage of pre-processing, quality control (QC-FC) metrics, FC matrices and carpet plots were visualised to ensure the processing step was having the desired effect of mitigating the impact of noise variables. A total of 193 scans survived our quality control procedure. A full quality control report can be accessed here: https://sidchop.github.io/STAGES_rs-fMRI/. To generate whole-brain FC matrices, we parcellated each individual's normalised scans into 300 cortical[7] and 32 subcortical regions[8]. We screened all regions for insufficient BOLD signal intensity by first calculating each region's mean BOLD signal across all 193 scans. We sorted the regional BOLD intensity values from largest to smallest and found the ''elbow'' of this distribution using the pairwise differences. This led us to exclude 16 cortical parcels, located across the orbitofrontal cortex and temporal pole. In the remainder of the analysis, we therefore used 316 regions. FC was estimated as the Pearson correlation between each pair of regional time series for each individual.

eMethods 6. Further Details on Statistical Analysis

Mixed-effects marginal models were used to analyse brain-wide FC changes across the three groups (MIPT, PIPT and controls) and three timepoints (baseline, 3-months and 12-months)[9]. At each of the 99,856 edges linking 316 regions, we computed ordinary least squares estimators of group-level regression parameters and a robust-covariance estimator to account for within-subject correlations. This method allows for robust and accurate estimation of random effects while mitigating problems posed by misspecification of covariance structure when using traditional mixed-effects model[9]. The approach also allows for brain-wide non-parametric computation of p-values at each edge using wild-bootstrapping (10,000

© 2021 Chopra S et al. *JAMA Psychiatry.*

bootstraps)[10]. All code used to analyse data and generate figures can be accessed here: https://github.com/sidchop/Stages_rs-fMRI/.

The Network Based Statistic (NBS) was used to perform family-wise error-corrected (FWE) inference at the level of connected-components of edges showing a common effect, resulting in a substantial boost in statistical power compared to mass univariate analysis[11]. The NBS procedure involves setting a primary component-forming threshold, τ, which is applied to both the observed data, and the bootstrap-generated null data. The choice of this threshold is arbitrary; more lenient thresholds will be sensitive to weaker differences distributed over a large number of edges while more stringent thresholds will be sensitive to stronger effects possibly extending over smaller subsets of edges. We report here results for τ set to $p < 0.05$ and show results for $p < 0.01$ and $p < 0.001$ in this Supplement. To compute accurate p-values for the null data, we used a modified parametric test developed specifically for unbiased inference on marginal models[9]. For both the observed and bootstrap-generated null data, the size (number of edges) of the connected components in the supra-thresholded network was recorded. The size of largest component from each bootstrap was used to build a null distribution and a corrected p-value for each observed component was estimated as the proportion of null component sizes that was larger than the observed value.

We evaluated NBS results using a Bonferroni-corrected threshold of $p_{FWE} < .016$, adjusted for three key contrasts. This first contrast addressed our first aim and simply tested for baseline differences between healthy controls and patients, collapsed across MIPT and PIPT groups. The second and third contrasts addressed our second study aim. The second contrast was designed to isolate differential FC changes over time in the antipsychotic-naïve patient (PIPT) group compared to the healthy control group. This contrast was defined as a group by time interaction examining changes in PIPT patients (excluding 3-month scans of three patients who were exposed to antipsychotic medication during the treatment period) compared to controls. Two individuals were exposed to antipsychotics at doses lower than the minimal exposure limit for inclusion in our study. Excluding these participants from the analysis yielded a qualitatively similar pattern of results. The third contrast was designed to isolate the specific effects of antipsychotic treatment by examining differential FC changes over time in the antipsychotic-treated (MIPT) group compared to both the PIPT and healthy control group. This contrast was defined as a conjunction based on the intersection of two

© 2021 Chopra S et al. *JAMA Psychiatry.*

subsidiary contrasts: the group-by-time interaction for MIPT patients vs controls and the group-by-time interaction for MIPT patients vs PIPT patients. This contrast maps FC changes in medicated patients that differ from both antipsychotic-naïve patients and healthy controls, thus isolating the effects of antipsychotic treatment most relevant to psychosis. In all three contrasts, the healthy controls provide an important normative benchmark against which to evaluate longitudinal changes in the patient groups (see also Chopra et al., 2020). To address our third study aim, we used non-parametric canonical correlation analysis (CCA) to investigate the relationships between FC changes (ΔFC) within any of the NBS subnetworks identified in the above analyses and changes in the two pre-registered primary trial outcome measures—SOFAS and BPRS total scores—across all patients.

All contrasts were adjusted for age, sex, and mean framewise displacement (head motion). All continuous covariates were centred. Age was split into between- and within-subject components, with both included as covariates in the design matrix[9].

To comprehensively delineate changes in FC across 49,928 different connections, present the results at three different scales: (1) the individual edge level, embedded in the spatial layout of the brain (e.g., Fig 1a; panel 1); (2) a level in which different regions are aggregated in one of 10 canonical brain networks, and we show the proportions of affected edges both within and between these networks (e.g., Fig 1a; panel 2); and (3) the level of individual brain regions, to identify specific brain areas which had a high number of significant connections (e.g., Fig 1a; panel 3).

Analysis of network level effects. To determine whether the observed FC changes showed any network-specificity, we calculated the proportion of edges within a given NBS component that fell within each of seven canonical brain networks[29] (e.g. Fig 1a – upper triangle of matrix). Different brain networks have intrinsic differences in their size, therefore we present both raw proportions and proportions normalized by the total number of possible network connections between each pair of networks (e.g. Fig 1a – lower triangle of matrix); the former identifies preferential involvement of a given network in an absolute sense while the latter accounts for differences in network size (i.e., the tendency for larger networks to be more likely to be implicated in a given NBS network).

© 2021 Chopra S et al. *JAMA Psychiatry.*

We used canonical correlation analysis (CCA) to investigate the relationships between FC changes within any NBS subnetworks and changes in symptoms and functioning across all patients. CCA is a multivariate statistical method that identifies linear combinations of two sets of variables that maximally correlate with each other. The statistical significance of the resulting canonical variates was assessed using permutation-testing[12]. First, we extracted FC estimates for each connection of the sub-network identified in the NBS analysis for both baseline and 3-months timepoints. Residual change scores were computed by regressing 3-months values on baseline values and retaining the residuals (ΔFC). We then summarized these high-dimensional edgewise change values using principal component analysis (PCA), retaining components that account for $>2\%$ of variance. Residual change scores for each clinical and functional scale were also computed by regressing 3-months values on baseline values and retaining the residuals. For each of the two identified sub-networks showing longitudinal changes, we computed a CCA assessing the correlation between ΔFC and changes in the two pre-registered primary trial outcome measures: SOFAS and BPRS total scores[1]. To assess statistical significance, we used a recently developed, robust and accurate non-parametric method for inference on the canonical variates[12]. The threshold for statistical significance was set to $p_{fwe} < 0.0125$, which is Bonferroni-corrected for four CCA analyses which included ΔFC from each if the primary longitudinal contrasts for both the baseline to 3-months and baseline to 12-months analysis. Thus, the p-values assessing the correlation between the variates for each CCA were corrected for the family of comparisons run within each CCA, and also corrected for the family of comparisons run across CCA analyses.

To assess which edges contributed most to the ΔFC canonical variate identified in the CCA, we computed the correlation between the residual change score at each edge and the ΔFC composite score. To assess statistical significance, we used robust bootstrapping to estimate the standard error at each edge, computed z-scores by dividing the correlation by the standard error and used these z-scores to compute two-tailed p-values[13]. Edges showing reliable loadings on the canonical variate were those that survived an FDR-correction of these p-values ($q < .05$).

Our secondary analysis repeated the two longitudinal contrasts from our primary analyses, except this time including the 12-month follow-up timepoint in addition to the 3-month timepoint. The contrasts of interest were linear polynomial contrasts examining differences in

© 2021 Chopra S et al. *JAMA Psychiatry*.

linear trend across all three timepoints. We constrain our contrasts in this way, because our hypotheses concern linear interactions between group and time over the follow-up period. The treatment period for the trial ended after 6-months and four PIPT patients commenced antipsychotic medication in this intervening period, in addition to the four patients who had commenced at the 3-month timepoint. Thus, between the 3-month and 12-month scan, a total of eight PIPT patients commenced antipsychotics, whereas all MIPT patients continued medication with varying degrees of exposure. We therefore specified a covariate quantifying cumulative exposure to antipsychotics (olanzapine equivalent, milligrams) for all eight patients within the PIPT group who were exposed to medication at the 3-month or 12-month timepoint. This procedure allowed us to statistically adjust for antipsychotic exposure in the PIPT group when attempting to disentangle the long-term change in FC in antipsychotic-naïve and antipsychotic-treated patients.

eMethods 7. Demographic and Sample Characteristics

We have previously reported some demographics and clinical characteristics of this cohort[14], but there are subtle differences in current sample compared to the previously reported sample (Table 1). Briefly, we detected no significant differences at baseline between patients and controls, but the patients were on average, younger and less educated. At baseline, the two patient groups (PIPT and MIPT) did not significantly differ in age, education, sex, handedness, BPRS or SOFAS scores. Additionally, the two patient groups did not significantly differ in overall substance use at baseline ($t = 0.235; p = 0.815$) or at 3-months ($t = -1.36; p = 0.184$). During the trial treatment period, the patient groups did not differ in the rates of antidepressant ($\chi^2 = 0.118; p = .732$), benzodiazepine ($\chi^2 = 2.94; p = .086$) or other ($\chi^2 = 0.567; p = .452$) medication exposure.

eAppendix 1. Antipsychotic-Naïve Results (Baseline)

Using τ-thresholds of $p < .01$ and $p < .001$, we observe that the strongest FC reductions in patients are concentrated in the limbic network and striatum, while the strongest FC increases occur between the thalamus and visual network (Sup. Fig 3).

eAppendix 2. Antipsychotic-Naïve Results (Baseline to 3 mo)

© 2021 Chopra S et al. *JAMA Psychiatry.*

Using τ-thresholds of $p < .01$ and $p < .001$, FC decreases in patients remain evenly distributed across networks. Strong FC increases in patients are especially concentrated in the DMN, limbic, and visual networks (Sup. Fig 3).

eAppendix 3. Antipsychotic-Related Results (Baseline to 3 mo)

Using τ-thresholds of $p < 0.01$ and $p < 0.001$, we find evidence that the strongest medication-related FC increases are concentrated in the visual, somatomotor, and attentional networks (Sup. Fig 3). This result aligns with our CCA of antipsychotic-naive changes over time, in which increased ΔFC in sensory networks is associated with improved functioning and symptoms over the first 3 months of illness.

Figure 3 – *Canonical Correlation Analysis between short-term functional connectivity changes and behavioural outcomes.* Canonical loadings of the principal components summarising change in FC (left), scatterplot of the correlation between the brain change and behaviour change canonical variates (middle), and canonical loadings for the change in the two primary trial outcome measures (right; SOFAS, social and occupational functioning assessment scale; BPRS, brief psychiatric rating scale).

eAppendix 4. Long-Term Changes in Antipsychotic-Naïve Patients Compared to Healthy Controls (Baseline to 12 mo)

A significant group-by-time interaction (Sup. Fig 1a-b; $p = 0.048_{fwe}$) was detected only at a τ-threshold of p<0.001 (33 edges), with 23 edges showing a decrease and 10 edges showing an increase over 12-months. This result suggests that long-term illness-related changes in FC are circumscribed to a relatively small subset of edges showing large effects. Edges showing reduced FC in patients over time predominantly implicated the default mode and somatomotor networks, with the right precuneus and bilateral post-central being the most heavily implicated brain regions. Edges showing increased FC in patients over time primarily linked DMN, visual, ventral attention and thalamic systems, with the left ventroposterior thalamus and right supra-marginal gyrus being the regions attached to the most edges showing increased FC over time.

eAppendix 5. Long-Term Antipsychotic-Related Changes (Baseline to 12 mo)

At a τ-threshold of p<.05, we identified a single NBS component showing an altered FC trajectory over 12-months in the MIPT group compared to the PIPT and control groups,

© 2021 Chopra S et al. *JAMA Psychiatry.*

comprising of 402 edges and including all 316 regions (Sup. Fig 1c-d; $p_{fwe} = .0084$). As with the 3-month results, medication was associated with a higher number of FC increases (302 edges) over time than decreases (100 edges). Raw counts indicate that these effects were concentrated in limbic, default, frontoparietal, somatomotor and ventral attention networks; normalized counts suggest a preferential concentration in the limbic system. Notably, the limbic network also showed lowered FC in patients at baseline. At a regional level, right prefrontal cortex, and left superior temporal gyrus and posterior hippocampus were strongly implicated in antipsychotic-related increases in FC. Edges showing medication-related decreases in FC were more diffusely spread across the networks, with a strong involvement of the left medial amygdala.

Using τ-thresholds of $p<0.01$ and $p<0.001$, we find evidence that the strongest effects for medication-related FC increases are concentrated in medial temporal areas and association and attentional networks (Sup Fig 3).

eAppendix 6. Association Between Long-Term Changes and Symptoms and Functioning

No significant associations were detected between Δ_{12}FC in the illness- and medication-related networks and primary outcome measures

© 2021 Chopra S et al. *JAMA Psychiatry.*

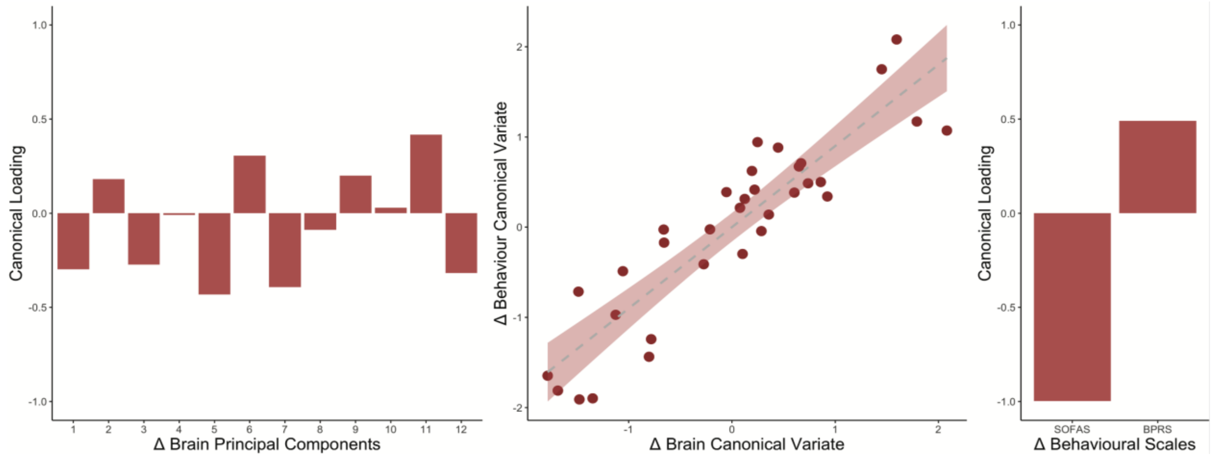

eFigure 2. Canonical Correlation Analysis Between Short-Term Functional Connectivity Changes and Behavioral Outcomes

Canonical loadings of the principal components summarising change in FC (left), scatterplot of the correlation between the brain change and behaviour change canonical variates (middle), and canonical loadings for the change in the two primary trial outcome measures (right; SOFAS, social and occupational functioning assessment scale; BPRS, brief psychiatric rating scale).

© 2021 Chopra S et al. *JAMA Psychiatry*.

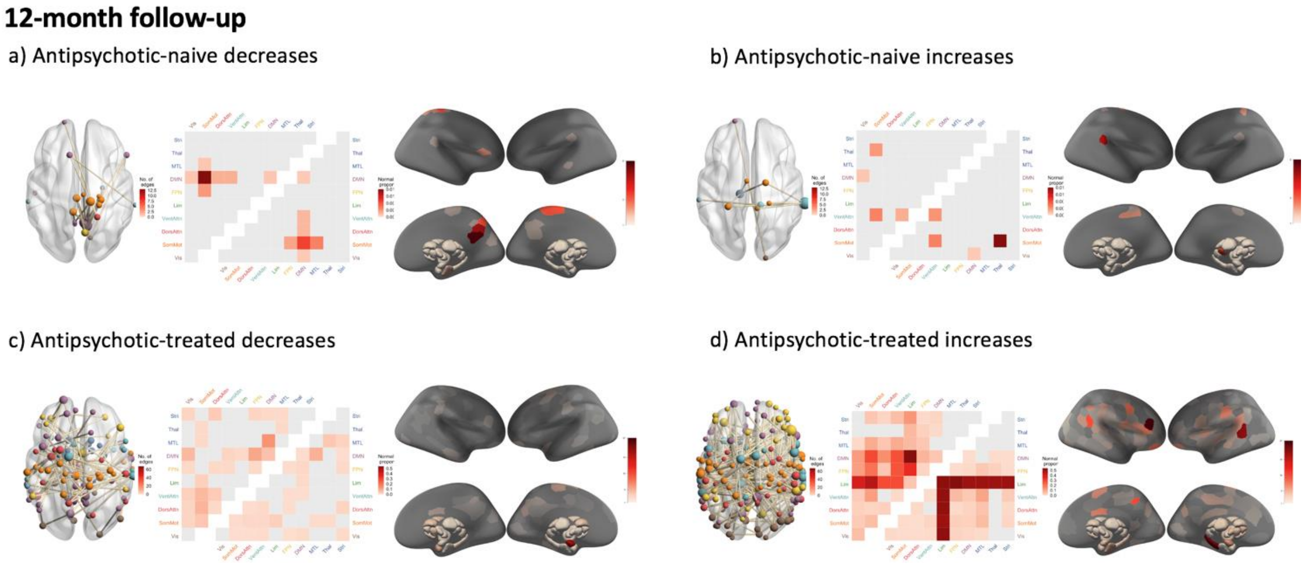

eFigure 3. Longer-Term Effects in Antipsychotic-Naïve Patients (A, B) and Longer-Term Effects Related to Antipsychotic Medication (C,D)

Each of the four panels contains three figures (from left to right): (1) a visualisation of the significant NBS subnetwork, with the nodes coloured by network and weighted by degree; (2) a heatmap of the proportion of edges within the NBS component that fall within each of the canonical networks as represented quantified using raw (upper triangle) and normalized (lower triangle) proportions; and (3) surface renedings depicted the number of edges in the NBS subnetwork attached to each brain region. Vis, Visual network; SomMot, Somatomotor network; DorsAttn, Dorsal Attention network; VentAttn, Ventral Attention network; Lim, Limbic network; FPN, Frontoparietal network; DMN, Default mode network; MTL, medial temporal lobe (amygdala and hippocampus).

© 2021 Chopra S et al. *JAMA Psychiatry*.

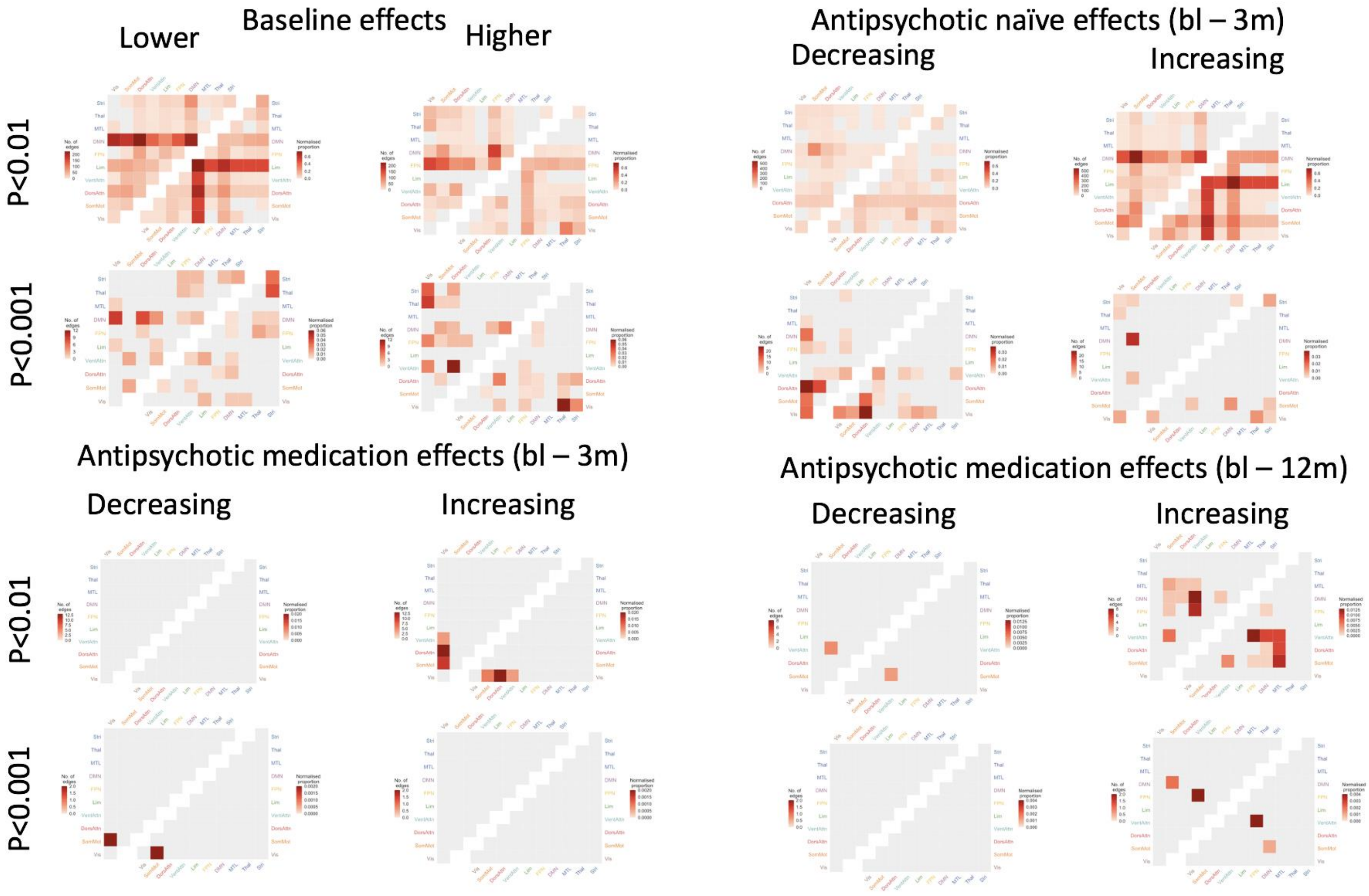

eFigure 4. Antipsychotic-Naïve and Antipsychotic-Treated Results at a Network Level (p_{fwe}<0.05) at Primary Thresholds of *P* < .01 and *P* < .001

© 2021 Chopra S et al. *JAMA Psychiatry*.

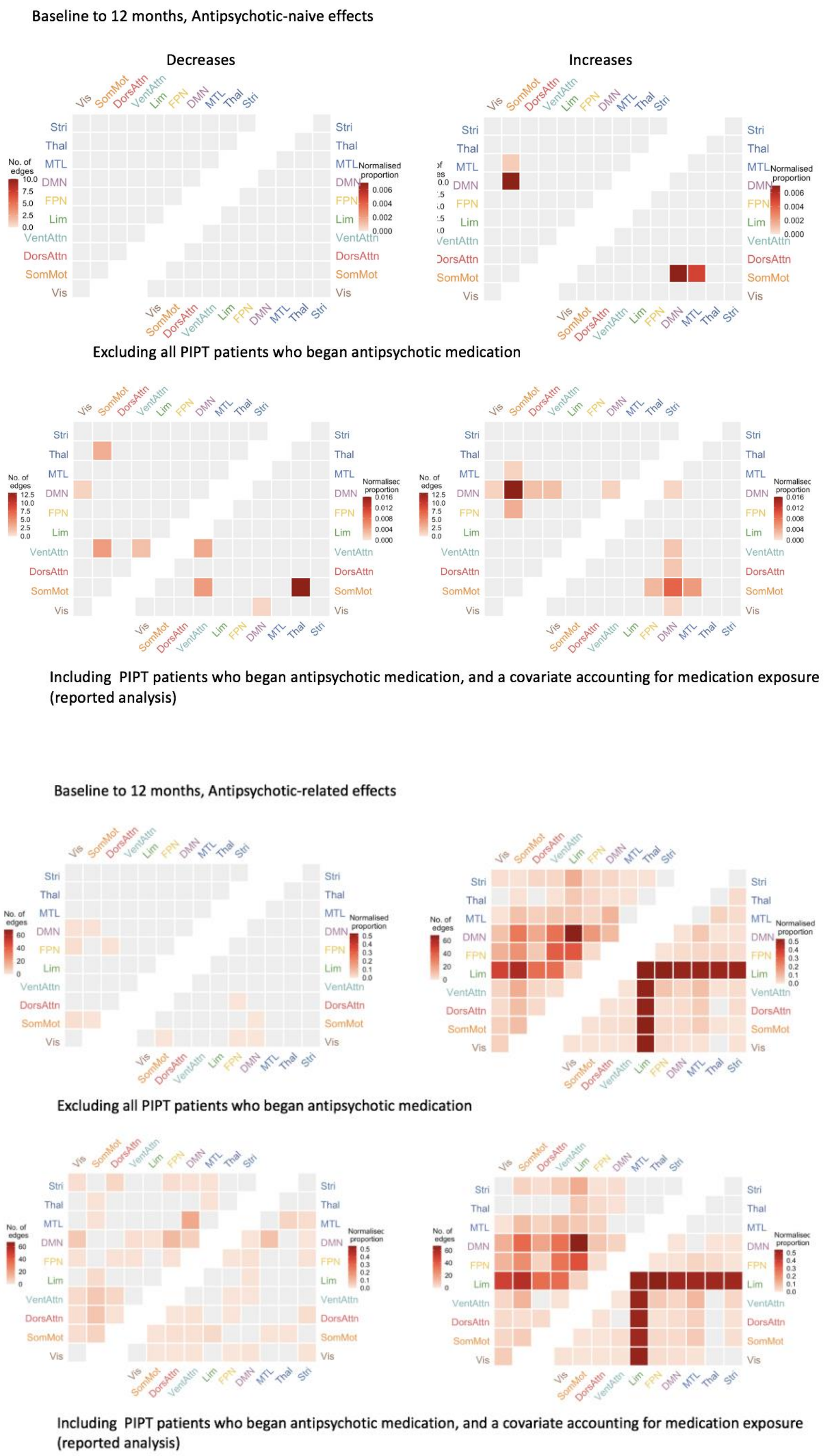

eFigure 5. Baseline to 12 Months Antipsychotic-Naïve and Antipsychotic-Related Effects After Removing the 9 Subjects in the PIPT Group Who Were Exposed to Antipsychotics

© 2021 Chopra S et al. *JAMA Psychiatry*.

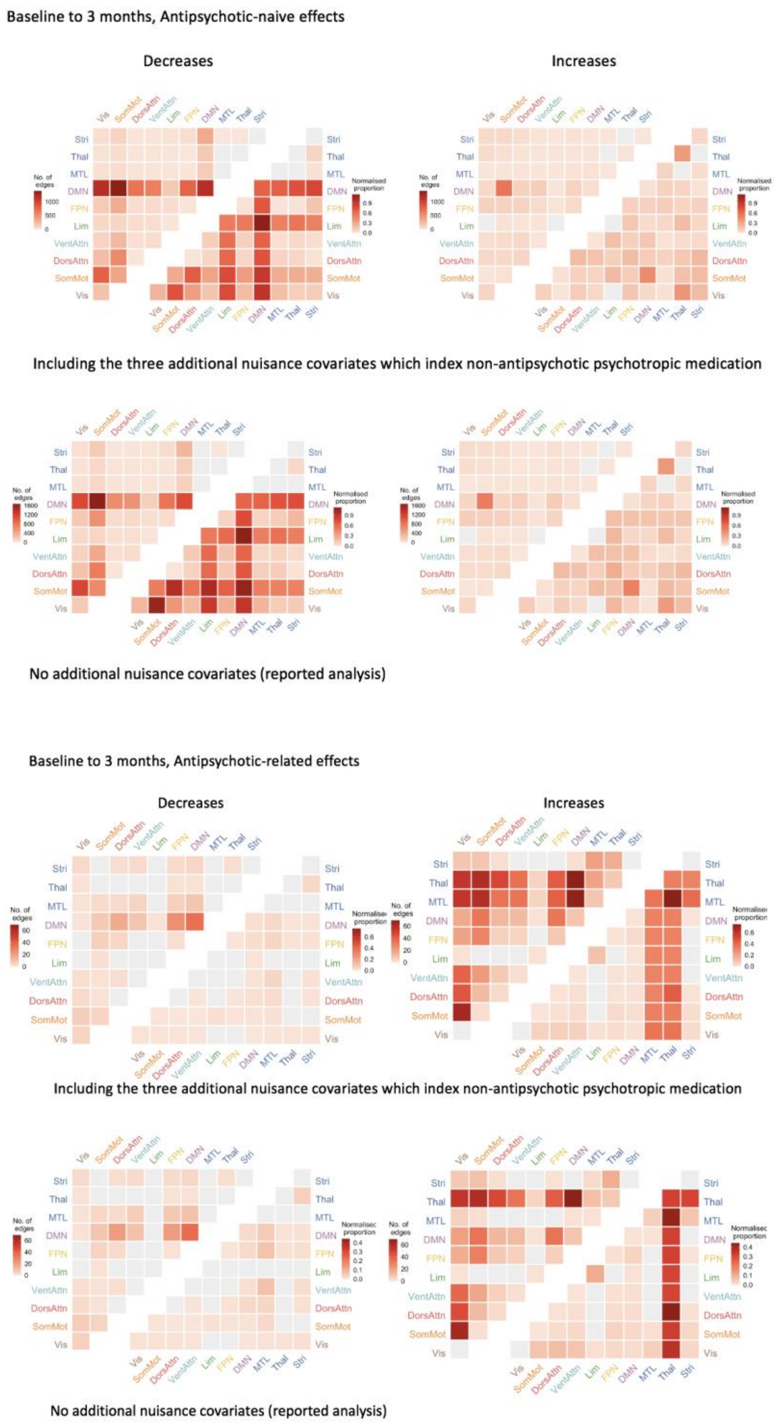

eFigure 6. Baseline to 3 Months Antipsychotic-Naïve and Antipsychotic-Related Effects After Adjusting for Nonantipsychotic Psychotropic Medication Use

© 2021 Chopra S et al. *JAMA Psychiatry.*

eReferences

1 Francey, S. M. *et al.* Psychosocial intervention with or without antipsychotic medication for first-episode psychosis: a randomized noninferiority clinical trial. *Schizophrenia Bulletin Open* **1**, sgaa015 (2020).

2 O'Donoghue, B. *et al.* Staged treatment and acceptability guidelines in early psychosis study (STAGES): A randomized placebo controlled trial of intensive psychosocial treatment plus or minus antipsychotic medication for first-episode psychosis with low-risk of self-harm or aggression. Study protocol and baseline characteristics of participants. *Early intervention in psychiatry* **13**, 953-960 (2019).

3 Esteban, O. *et al.* MRIQC: Advancing the automatic prediction of image quality in MRI from unseen sites. *PLoS One* **12**, e0184661, doi:10.1371/journal.pone.0184661 (2017).

4 Esteban, O. *et al.* fMRIPrep: a robust preprocessing pipeline for functional MRI. *Nature methods* **16**, 111-116 (2019).

5 Parkes, L., Fulcher, B., Yücel, M. & Fornito, A. An evaluation of the efficacy, reliability, and sensitivity of motion correction strategies for resting-state functional MRI. *Neuroimage* **171**, 415-436 (2018).

6 Pruim, R. H. *et al.* ICA-AROMA: A robust ICA-based strategy for removing motion artifacts from fMRI data. *Neuroimage* **112**, 267-277 (2015).

7 Schaefer, A. *et al.* Local-Global Parcellation of the Human Cerebral Cortex from Intrinsic Functional Connectivity MRI. *Cereb Cortex* **28**, 3095-3114, doi:10.1093/cercor/bhx179 (2018).

8 Tian, Y., Margulies, D. S., Breakspear, M. & Zalesky, A. Topographic organization of the human subcortex unveiled with functional connectivity gradients. *Nature neuroscience* **23**, 1421-1432 (2020).

9 Guillaume, B. *et al.* Fast and accurate modelling of longitudinal and repeated measures neuroimaging data. *Neuroimage* **94**, 287-302, doi:10.1016/j.neuroimage.2014.03.029 (2014).

10 Guillaume, B. & Nichols, T. Non-parametric Inference for Longitudinal and Repeated-Measures Neuroimaging Data with the Wild Bootstrap. *Poster presented at the Organization for Human Brain Mapping (OHBM) in Hawaii* (2015).

11 Zalesky, A., Fornito, A. & Bullmore, E. T. Network-based statistic: identifying differences in brain networks. *Neuroimage* **53**, 1197-1207 (2010).

12 Winkler, A. M., Renaud, O., Smith, S. M. & Nichols, T. E. Permutation inference for canonical correlation analysis. *Neuroimage* **220**, 117065, doi:10.1016/j.neuroimage.2020.117065 (2020).

13 Zimmermann, J., Griffiths, J. D. & McIntosh, A. R. Unique Mapping of Structural and Functional Connectivity on Cognition. *J Neurosci* **38**, 9658-9667, doi:10.1523/JNEUROSCI.0900-18.2018 (2018).

14 Chopra, S. *et al.* Differentiating the Effect of Medication and Illness on Brain Volume Reductions in First-Episode Psychosis: A Longitudinal, Randomized, Triple-blind, Placebo-controlled MRI study. *medRxiv* (2020).

© 2021 Chopra S et al. *JAMA Psychiatry.*
