# Supplementary material for: Functional Connectivity in Antipsychotic-Treated and Antipsychotic-Naive Patients With First-Episode Psychosis and Low Risk of Self-harm or Aggression: A Secondary Analysis of a Randomized Clinical Trial
Source: JAMA Psychiatry. 2021 Jun 23;78(9):1–11. doi: 10.1001/jamapsychiatry.2021.1422 (PMC8223142; doi:10.1001/jamapsychiatry.2021.1422)
Supplement: Supplement 4. — Data Sharing Statement [file jamapsychiatry-e211422-s004.pdf]

Data Sharing Statement

Chopra. Functional Connectivity in Antipsychotic-Treated and Antipsychotic-Naive Patients With First-Episode Psychosis and Low Risk of Self-harm or Aggression. *JAMA Psychiatry*. Published June 23, 2021. doi:10.1001/jamapsychiatry.2021.1422

Data
Data available: No

Additional Information
Explanation for why data not available: Data will be made available upon request and review by the clinical trial team.
